# Influence of alcohol provocation on medical professionals in Taiwan: A qualitative study

**DOI:** 10.1371/journal.pone.0264071

**Published:** 2022-02-16

**Authors:** Ching-Yi Lee, Ching-Hsin Lee, Hung-Yi Lai, Mi-Mi Chen

**Affiliations:** 1 Department of Neurosurgery, Chang Gung Memorial Hospital at Linkou, Taoyuan, Taiwan; 2 College of Medicine, Chang Gung University, Taoyuan, Taiwan; 3 Department of Radiation Oncology, Proton and Radiation Therapy Center, Chang Gung Memorial Hospital at Linkou, Taoyuan, Taiwan; Imam Abdulrahman Bin Faisal University, SAUDI ARABIA

## Abstract

There is a paucity of research on the issue of alcohol provocation in the medical field. While studies have been performed concerning alcohol abuse among students, no studies have concentrated on alcohol provocation among medical professionals. Therefore, it is essential to look at the underlying factors that may influence alcohol use by medical professionals. A qualitative study using focus groups was conducted to construct themes depicting medical professionals’ experiences of alcohol provocation. Physicians (n = 32) and residents (n = 29) were recruited from a large teaching hospital in Taiwan. The volunteers included both subjects and instigators of alcohol provocation (individuals being pressured to drink and those who exert such pressure on others). A questionnaire on their alcohol use was used to quantitatively assess the prevalence of alcohol consumption and inebriation. The participants were then interviewed separately in groups. All interview data were recorded, transcribed and analysed thematically. A notable prevalence of recent alcohol consumption was observed in both the physicians (n = 18, 56%) and residents (n = 17, 59%). Three prominent themes were identified and summarized: (1) Social drinking in the Taiwanese medical profession (2) Workplace hierarchy and changes in drinking culture, and (3) Influence on the medical profession. The behaviour of alcohol provocation among these medical professionals was revealed with its underlying factors of specific cultural norms, workplace hierarchy and social expectations. An understanding of alcohol provocation helps increase the awareness of adverse consequences associated with alcohol provocation, encourage medical professionals to avoid inappropriate drinking behaviors, and reduce the risk of compromising medical professionalism.

## Introduction

### Alcohol use in the medical profession

Medical professionalism is an area of importance for all health care practitioners [1]. Alcohol use in the medical profession has been frequently reported for decades [2–6]. Not only is this behavior of intoxication inflicting a damaging impression on medical professionalism, but most notably it is a major threat to patient safety [7]. Most health care professionals maintain the same habits as the general populace, which includes an occasional drink while relaxing after work and heavier drinking associated with social functions [8–10]. Drinking in excess to the point of inebriation, however, is not generally associated with professional medical behavior. While this general statement is true, there are still instances where medical professionals were reported taking alcohol at work or being inebriated upon reporting for duty [4, 6, 11–13]. Both of these activities are concerning as alcohol use impairs judgment and impacts the ability of medical professionals to provide accurate and competent health care.

The majority of current research consists of quantitative studies on alcohol abuse in medical professionals [4, 6, 8–13]. These studies concentrate on the prevalence and impact of alcohol abuse in medical professionals or identification of alcohol impaired medical professionals [4, 6, 8–13]. The studies have reported that medical professionals may examine patients and make medical decisions while inebriated and that the medical professional in question may not feel that inebriation impacts their ability to make accurate and correct medical judgments [4, 6, 8–13].

### Drinking behaviours and provocation among medical students

Articles concerning alcohol use among university students consistently relate their drinking behaviours to gender, religion, culture and social norms such as peer influences [14–17]. A majority of studies report how students may be pressured into drinking excessive alcohol by their peers [1, 14–17]. One qualitative study describes medical students engaging in acts of forced drinking that could cause distress to their peers [1]. This study conceptualizes a drinking behaviour as “alcohol-related provocation”, which refers to any action that can make others feel coerced or compelled to consume alcohol or drink more alcohol than intended [1].

### Drinking culture

Western drinking practices may take place frequently both in everyday social occasions or in private households for various purposes at all scales of alcohol consumption [18]. In Taiwan, like in many other Chinese societies, alcohol consumption is typically associated with drinking occasions during celebrations for holidays such as the Lunar New Year, or is specifically recognized as an important facilitator for business and work settings [19–21]. It is a tradition for Japanese employees to attend “Bonenkai” events which are drinking parties commonly held by workplaces at the end of each year to give thanks to others [22]. Similar to this custom, the “Weiya” events known as annual workplace banquets are also observed in Chinese societies (including Taiwan) [23]. Most hospitals in Taiwan also organise annual banquets (Weiya drinking parties) which are unquestionably a part of this specific workplace drinking culture. Another unique drinking etiquette identified with workplace banquets in Chinese societies is “quanjiu”, which describes the custom of urging others to drink [24]. This drinking habit could be considered a type of alcohol provocation behaviour [20, 24]. In Taiwanese hospitals where alcohol consumption is inevitable at social functions such as department gatherings or year-end parties, medical professionals are often required to attend these social events and the occurrence of alcohol provocation can be commonplace. While studies have provided insights into alcohol use and alcohol provocation among medical students, no studies have addressed experiences of alcohol provocation among medical professionals in the Asian context.

### The present study

The purpose of this study is to interview medical professionals to gain a better understanding of how alcohol provocation occurs and the impact of alcohol provocation on medical professionals. The use of a qualitative method allows for a more open format to identify hypotheses and common themes that can enhance our understanding of alcohol provocation in medical professionals from multiple perspectives and cultural backgrounds. It is anticipated that understanding of alcohol provocation would allow the development of effective interventions targeted at reducing the risk of alcohol abuse in medical professionals, and hence lowering the likelihood of compromising medical professionalism and patient safety.

## Methods

### Setting

This qualitative study involving focus groups was undertaken between December 2015 and January 2016 in a large teaching hospital in Taiwan. In accordance with the standards for reporting qualitative research and ensuring rigor in this study, the framework of this methodology involves the following steps: sampling of participants, data collection and data analysis [25, 26]. This study was approved by the Chang Gung Medical Foundation Institutional Review Board on July 7, 2014 (Ref.: 103-7771B).

### Sampling of participants

The participants comprised residents and physicians recruited from the local hospital using targeted emails, memos, and flyers. All participants were selected using purposive sampling technique [27]. The study recruited a total of 61 participants comprising 32 physicians (mean age of 43 years; 27 males) and 29 residents (mean age of 27 years; 18 males). The most common specialty found among the group of physicians was neurosurgery (n = 16) while the rest were general surgery (n = 1), colon and rectal surgery (n = 1), cardiac surgery (n = 1), thoracic surgery (n = 1), physical medicine and rehabilitation (n = 1), orthopedics (n = 1), thoracic medicine (n = 1), nephrology (n = 2), emergency medicine (n = 2), internal medicine (n = 1), pediatrics (n = 1), neonatal–perinatal medicine (n = 1), dermatology (n = 1) and radiology (n = 1). In the group of residents, there were 19 participants in their first year of post graduate rotational training, 8 neurosurgical trainees and 2 general surgical trainees. No participants withdrew from either group. All participants were informed about the purpose and voluntary nature of the study. Written consent forms were obtained from all individual participants included in the study.

### Data collection

All recruited participants (n = 61) volunteered to take part in focus groups. A questionnaire regarding prevalence of alcohol consumption was given to each participant prior to their interview. Alcohol consumption or alcohol inebriation were assessed respectively by asking the participants “Have you consumed any alcohol in the last year?” and “Have you gotten drunk in the last year?” Those who answered yes to each question were further asked respectively about the frequency of their alcohol consumption or inebriation. Participants were also asked to self-report if they have ever taken alcohol or been drunk while on duty. These questions were reviewed by the current research team before administration. The number of participants included in each focus group ranged from 4 to 6. Residents and physicians were sometimes interviewed in separate focus groups due to circumstances where residents might have to comment on their superiors. A total of 12 focus groups followed a topic guide developed by all members in the research team (S1 File). Interview questions were reviewed and approved by the Institutional Review Board (Ref.: 103-7771B). The duration for every interview held in a private meeting room was between 50 and 70 minutes. Interviews were audio-recorded and conducted in Mandarin (participants’ native language) by a researcher (MMC, a member of our research team) trained in qualitative studies. The interviewer guided the process with the goal of encouraging group discussions in an open-ended manner and ensured that leading questions were kept to a minimum to prevent interviewer bias. Narratives of personal experiences were also encouraged to ground the data in actual experiences rather than merely opinions. Notes were taken during the interviews by CHL, another researcher from our team. Both researchers, MMC and CHL are not members of the departments from which the participants were recruited.

### Data analysis

The audio recordings were transcribed verbatim (see S2 File) and to ensure anonymity during data analysis, participants were assigned a unique identifier. All transcripts and notes were analyzed thematically to identify, analyze and report patterns within the data [28]. The software ATLAS.ti was utilized for data management and coding. All transcripts and results were translated into English by a paid translator after thematic analysis. Two researchers from our team, CYL and CHL, who familiarized themselves with the transcripts through repetitive reading completed the first coding round of four initial transcripts. A third researcher from our team, HYL coded the interview data of four focus groups independently. A coding scheme was developed by CYL and CHL and reviewed by HYL. A second coding round was then performed and all interview transcripts were coded separately by CYL and CHL. Both versions of coding results were compared to reveal differences in coding. CYL, CHL and HYL discussed discrepancies to resolve them and reach an agreement on the labeling and definitions of codes. There was no need for further data collection once no new ideas or themes emerged from the data. All researchers reached consensus on the final analytical framework of common themes across all interviews. The quantitative data collected from questionnaires were analyzed using Statistical Product and Service Solutions (SPSS 24) software.

### Ethics approval

Written informed consent was obtained from all individual participants included in the study. This study was carried out in accordance with relevant guidelines and regulations, and approved by the Chang Gung Medical Foundation Institutional Review Board on July 7, 2014 (Ref.: 103-7771B).

## Results

The response rate was 100%, and that all participants answered all questions without any refusal or hesitation. We begin by presenting self-reports of alcohol consumption by participants, following this, we report our thematic findings. The self-reports were questionnaire results obtained from the interviewed physicians and residents in the Taiwanese hospital. When asked to report on the prevalence of alcohol consumption on a daily, weekly, monthly or yearly basis, 56% of physician participants (n = 18) and 59% of the residents (n = 17) self-reported having used alcohol recently. The resident participants showed a significantly higher prevalence of monthly consumption of alcohol than the physician participants (χ2 = 5.306, p < 0.05). With regard to the prevalence of alcohol inebriation, 6.3% of the physicians (n = 2) and 17.2% of the residents (n = 5) had recently been inebriated. Details are shown in **Table 1.** When asked, “Have you ever taken alcohol or been drunk while on duty?”, only one resident out of all participants claimed to have consumed alcohol and been inebriated while on duty.

**Table 1:** Prevalence of alcohol consumption and inebriation among physicians and residents.

Use of Alcohol	Physician (n = 32)	Resident (n = 29)	χ^2^ and p-value
	n (%)	n (%)	
Daily Consumption			
Yes	1 (3.1)	0 (0)	Fisher’s Exact
No	31 (96.9)	29 (100)	Test p = 1.000
Weekly Consumption			
Yes	8 (25)	5 (17.2)	**χ**^**2**^ = 0.546
No	24 (75)	24 (82.8)	p = 0.460
Monthly Consumption			
Yes	4 (12.5)	11 (37.9)	**χ**^**2**^ = 5.306
No	28 (87.5)	18 (62.1)	**p = 0.021** ^*^
Yearly Consumption			
Yes	5 (15.6)	1 (3.4)	Fisher’s Exact
No	27 (84.4)	28 (96.6)	Test p = 0.198
Daily Inebriation			
Yes	0 (0)	0 (0)	
No	32 (100)	29 (100)	Not Applicable
Weekly Inebriation			
Yes	0 (0)	1 (3.4)	Fisher’s Exact
No	32 (100)	28 (96.6)	Test p = 0.475
Monthly Inebriation			
Yes	1 (3.1)	2 (6.9)	Fisher’s Exact
No	31 (96.9)	27 (93.1)	Test p = 0.600
Yearly Inebriation			
Yes	1 (3.1)	2 (6.9)	Fisher’s Exact
No	31 (96.9)	27 (93.1)	Test p = 0.600

*p < 0.05.

In this study, the qualitative data obtained from the focus group approach was analyzed and coded to generate 3 major themes based on the narratives of physicians and residents. The themes were: (1) Social drinking in the Taiwanese medical profession (2) Workplace hierarchy and changes in drinking culture, and (3) Influence on the medical profession. A summary of the themes is provided in **Table 2.** Illustrative quotes (with contextual additions shown in square brackets) from participants are given after the explanation of each theme below. The participant unique identifier is denoted by letters representing either a physician (P) or resident (R) and numbers showing the focus group s/he was from in round brackets, as follows: (physician/resident; focus group number; participant number).

**Table 2:** Three major themes identified with explanations and example quotations.

**Theme 1. Social drinking in the Taiwanese medical profession**	
Explanation	It is common for physicians or residents to participate in several drinking events organized by hospital departments. The way medical professionals behave during a drinking gathering has been influenced by Japanese drinking culture. In both Taiwanese and Japanese traditions, drinking at social functions is inevitable and considered essential to demonstrate adherence to social etiquette. The drinking behavior of making a toast to teachers is often encouraged to show respect or gratitude. However, engaging in such social drinking traditions is also associated with dissatisfaction.
Quotations	I think [our] drinking [culture] comes from Japan and Korea. . . In Taiwan, urging others to drink is seen as common as what you see in Japan and Korea where the younger ones have to make a toast to elders for showing a respect to someone, this is something that I reckon is pointless. (R6-3)
	But then the [drinking] custom [has] gone bad. Sometimes you don’t like to drink or make a toast, but you get the feeling of kinda being forced that you have to, showing your manners, or just to drink out of courtesy [to teachers]. (P8-3)
**Theme 2. Workplace hierarchy and changes in drinking culture**	
Explanation	It is noted that senior physicians or superiors have the tendency to compel others to drink, with the possible intention of demonstrating their superiority. Most residents and physicians have perceived pressure to drink at gatherings as a result of differences in workplace hierarchy. A drinking behavior related to alcohol provocation was frequently reported as the act of forcing others to drink, make a toast or even drink excessively. Alcohol provocation in Taiwanese medical profession does not occur only among the junior and senior physicians but also between peer colleagues. It seems the behavior of urging others to drink has been passed on through generations until recently, when there has been a gradual transformation in such drinking culture. Alcohol provocation at present is not as prevailing as before because some participants believe everyone has free will to decide whether or not to follow this drinking tradition.
Quotations	…You can’t help (avoid) it [drinking] if you happen to drink with someone in a higher position than you, pressure from positional rank. …We are from a lower rank and drinking with those with a senior rank who will always urge us to drink. (R1-1)
	There is a senior attending physician making a rule of drinking the number of glasses with respect to the year of the residency of the person you are drinking with, meaning that if you are a second-year resident while I am a fifth-year resident, you have to take 5 glasses and I only have to drink 2 glasses. (R12-2)
	Back in the time, physicians [of higher ranks] would come to ask you to drink with them or you had to make a toast to your teachers. It was hard to avoid. This is different comparing to the present [time], where more consideration [for others] is seen among colleagues. Different century represents people with different perspectives. (P10-3)
**Theme 3. Influence on the medical profession**	
Explanation	Participants pointed out how alcohol provocation encountered at these drinking events often leads to situations where they are pressured into binge drinking, which, in turn, places them at risk of losing consciousness, hospitalization, absence from work, or even compromised work performance. Moreover, the behavior of alcohol provocation can leave a bad impression on colleagues.
Quotations	… drinking way too much till I dropped on the day before [work]. I was definitely late for the next day. I was feeling dizzy without knowing what to do at the operating room…(R1-6)
	…after coming back from drinking at a gathering, one senior chief physician went to [visit] the wrong patient while making ward rounds. (R3-3)
	For those who don’t favor this particular social drinking behavior, like me, if I happen to meet someone [from work] who keeps urging me to drink or make a toast, I would definitely find this person annoying (P5-2)

**Quotations:** (physician/resident; focus group number; participant number).

### Theme 1. Social drinking in the Taiwanese medical profession

Most participants provided their experience with work-related gatherings where alcohol consumption was always involved. By toasting drinks to meet with others, drinking alcohol at these events was believed a way of showing respect and adherence to social etiquette.

*We have this tradition where the younger doctors have to line up to make a toast to the elders for showing respect and gratitude for taking care of them throughout the year*. *We only do it for the sake of this tradition*. *(P9-3)*

*[Drinking or toasting is] a social behavior or to maintain a close relationship*, *to put it in a nice way*. *Formally speaking*, *it’s also a type of etiquette*. *(P3-2)*

*It’s also to show your manners*. *It wouldn’t look nice to take out the juice*. *Drinking wine and proposing a toast [at the dinner event]is not only being polite but also showing your manners*. *(R12-1)*

Some participants stated that the regular year-end drinking party is a typical drinking culture originating from Japan, which shares similar characteristics of workplace social drinking with Taiwan. The year-end party or department gatherings were frequently mentioned by both physicians and residents throughout the transcripts as regular drinking occasions organized by the hospital. Specifically for most residents, other workplace drinking occasions also include welcome parties for freshmen or mentor support gatherings.

*The year-end party which everyone goes to is meant for “forgetting about [anything unpleasant happened during] the year*” *and a tradition from Japan*. *Juniors make a [drinking] toast to the seniors at the year-end party*. *(P5-5)*

*It’s more like a Japanese [drinking] culture*, *you have to make a toast if eating with elders and teachers*, *and when there is alcohol provided on the table*. *(P4-3)*

*…we have this mentor support gatherings (an organized drinking event where a mentoring physician meets their students) but the worst case I had witnessed was at the welcome parties*, *some were forced drinking and got so drunk till they passed out*. *(R12-4)*

The tradition of expressing gratitude by toasting drinks to teachers was considered by some participants to be beneficial for their medical career. Some participants felt this drinking behavior was likely to be associated with specialties where an apprenticeship is valued.

*I think attending these gatherings always helps your career in some way by getting to know more people to enhance your interpersonal relationships [with other doctors or teachers]… It’s hard to avoid [making toasts to teachers] if you are from the surgery (department)*. *(R6-2)*

*You felt like you had to drink because you were from the surgery department. Experienced specialists who taught me things about operations*, *I wanted to make a toast to show my gratitude to them and other seniors. (P4-5)*

*There is this apprenticeship system [commonly seen] at the surgery division*. *The relationship between a mentor and an apprentice*. *And they [the teachers and apprentices] become closer to each other without feeling a wall [barrier] between them when they get together to drink after work*. *(R6-1)*

On the other hand, it was regarded as pressure for some participants to be present at these drinking events. There were concerns raised about their career if failing to follow this drinking tradition in the workplace.

*I think there is pressure when some supervisor is present or for some particular occasion where it makes you that you have to [drink and make a toast]*. *(P8-2)*

*Some people might think it’s not polite [to skip] making a toast to your teachers*. *Would this have an effect on your job*? *There might be*. *I think there is*. *The custom of this society leads to this particular etiquette*. *(P3-4)*

### Theme 2. Workplace hierarchy and changes in drinking culture

The difference in hierarchy in the workplace means the presence of various ranking of positions, status and power, leading to inequality among employees. On many drinking occasions, physicians who were more inclined to display superiority would often lead the residents to propose a toast not only to their teachers but also to many others. Young doctors or those with a junior rank might be manipulated by any authority figure to take alcohol unwillingly or in excess. Participants pointed out a regular scene from workplace drinking events where an unequal amount of alcohol consumption occurs. In this case, a junior might have to drink in response to several drinking toasts made by teachers or colleagues while the teachers would only have to take one glass of drink in response to all of them. There is obvious unfair treatment due to rank distinction observed at drinking events where doctors of junior rank were more likely to become inebriated. Most of the participants expressed a feeling of compulsory drinking or requirement to “out-drink” due to workplace hierarchy:

*People drink alcohol and make toasts to one another and it is a must*! *I remember myself at a younger age preparing [alcoholic] drinks for making toasts to teachers*. *Unfairness in [the amount of] drinking alcohol happened frequently*. *The situation where you had to drink a whole glass or more while your teacher only took a sip was pretty common*. *(P9-2)*

*…A teacher [supervisor or superior] would come to me with a bottle of wine asking how many glasses [of drinks] I would drink in response to his drinking toast*. *He would say it’s a must to out-drink your teachers*. *Some people drank until they threw up…*. *Once at one gathering for the surgery department*, *the superintendent came to make a toast to you*, *you had to drink 5 glasses while he only had to finish one glass*. *(P2-4)*

Several physicians reported the term “quanjiu”, which describes the behavior of urging others to take alcohol or making others drink excessively. The situation where doctors forcing others to drink or being pressured into drinking at gatherings fits into the concept of “alcohol provocation”. Some participants who might have had no intention to drink could have been forced to drink in excess under the influence of provocation from superiors.

*It depends on what your chief physician is like*. *Our former chief physician enjoyed drinking quite a lot and he forced us to drink until we all*, *even the female (colleagues) got drunk*. *(P9-1)*

*Students made drinking toasts to the teachers while teachers “quanjiu” to the students (made the students drink)… It was a mess as everyone was making a toast and urging others to drink*. *We would choose to hide in the back unless if someone comes to your table and says* “*Come on*, *let’s drink more tonight and get drunk a bit more”*, *we would pretend to drink but spit it out later*. *(R6-1)*

Similar provocation occurred when colleagues or bystanders also played a role in urging others who were then obliged to drink, as described by one of the physicians:

*As I recall*, *it is usually those bystanders (who may not necessarily be superiors) or colleagues who happen to urge you to drink 3 glasses of alcohol instead of one glass as originally demanded by the teacher*. *…Those bystanders (staff from other departments)*, *colleagues or superiors are those held responsible for making this kinda demand*. *(P4-3)*

Drinking provocation found in the Taiwanese medical profession, either from superiors or peers, was once believed to be passed on as a tradition through generations. It is now viewed as an optional convention which is seldom followed or practiced by the residents or physicians of current generations.

*… All the doctors had been through the same kind of ordeal (provocation) before when they were juniors*. *Once they turned to be senior doctors*, *they would expect the young fresh doctors to greet them with drinking toasts… The fact of being his [the junior’s] teacher is the biggest reason good enough to make the juniors drink… [It’s like] I was being mistreated in the same way before and now it is finally my turn to be in the reverse position [of making others drink]*. *(P2-5)*

*There would be differences as times have changed*. *The time when I was a resident [but comparing to] the present day where the spirit of democracy encourages people to value personal rights and protect interests of others*. *Not many residents [nowadays] will be willing to drink*,*…*. *I might get kicked out [if I didn’t follow to drink] in the past time*. *It’s changed now*. *You could hardly see anyone being forced to drink [at any current drinking event] nowadays*, *no such thing would occur*, *whether it [drinking] be between peers or [among a group of] junior and senior doctors*. *(P11-3)*

### Theme 3. Influence on the medical profession

At workplace drinking events where alcohol provocation was common, some participants were often unaware of how much alcohol they could tolerate until they were made to drink excessively to the point of losing consciousness. In addition to the anticipated adverse effects of drinking on health, participants who experienced any form of alcohol provocation suffered severe consequences including hospitalization, absence from work, or compromised work performance.

*There is always impact from [alcohol] provocation*. *It leads to binge drinking*. *[What’s worse]is the next day you have to show up at work and feeling all dizzy and sleepy as a result from excessive drinking*, *as well as the headache [you get] from hangover*. *My work performance was slightly affected but luckily no serious accident occurred*. *(P3-2)*

..*it is always to make the first year residents to drink till they drop at the welcome parties*. *The affected freshmen residents always ended up in the emergency room*. *(R6-5)*

*I managed to show up at work for most of the time after the year-end drinking parties I attended (the day before)*. *Occasionally*, *I really did fail to show up (because of forced binge drinking on the previous day*, *then [my] supervisor or colleagues would have to take over my work at the emergency room*. *(P5-1)*

Participants expressed a feeling of disapproval of persistent persuasion from colleagues, even if they tried to resist alcohol provocation, and how this leads to a negative impact on the relationship with colleagues as some may regarded it as an act of bullying.

*I personally disapprove of such behavior where you are being pressured consistently into drinking despite the effort of a polite decline is made*. *Eventually*, *you end up in having a bad impression on the person doing so*. *In my opinion*, *it is not a decent behavior to some extent*. *(P8-3)*

*Don’t overdo it [provocation]*, *try to control yourself*. *What you behave at the drinking event may influence on how your colleagues see you*. *It has some effect on your professional image or the relationship you have with your colleagues*. *(P10-1)*

*The bad thing about it [alcohol provocation]*, *some people reckon it is more fun to deliberately force those who gets drunk easily to drink*. *It’s perceived an act of bullying (from colleagues) by those people who can’t really drink*. *(P5-3)*

## Discussion

This study reveals that the incidents of alcohol provocation, a behavior of forced drinking by others, are encountered by medical professionals in Taiwan and are often associated with drinking occasions organized by the workplace. Some studies provide insights into how cultural norms may shape an individual’s drinking behavior [29, 30]. The convention of year-end parties initiated by workplaces in Taiwan is in fact a tradition resembling the social functions observed in workplaces in Japan and Korea [31, 32]. A drinking behavior typically embedded in this Asian culture is driven by the idea of practicing the good virtue of showing gratitude to teachers or superiors by drinking or making a toast [33]. In our study, medical professionals who normalized this tradition in an attempt to ensure social etiquette would eventually adopt a behavior of urging others to drink. Researches investigating workplace drinking gatherings in Taiwanese or Japanese societies also provide explanations for forced drinking behavior based on the similar influence of social contexts [31, 34]. What may have started out as a good intention to display courtesy, toasting behavior has gradually become a social requirement leading to forced drinking. This is how alcohol provocation subject to social etiquette occurred when medical professionals were obliged to drink due to cultural conformity rather than a voluntary intention to drink.

Another factor to consider that contributes to alcohol provocation is linked to workplace hierarchy. In most studies investigating correlates of alcohol consumption among Western workers, their general focus is on work stress, age or gender of participants and workplace norms [35, 36]. Whether workplaces presenting an environment supportive of drinking, the frequency of drinking gatherings after work and the availability of alcohol at the workplace are all considered workplace norms which can affect the occurrence of alcohol use among workers [35, 36]. In contrast to these studies, workplace hierarchy is specifically mentioned as one workplace norm predicting alcohol use and therefore causing alcohol provocation among workers in Asian societies [37–39]. One study on Korean drinking culture illustrates how the employees might feel compelled to “fit in” at drinking occasions in an attempt to please their managers [32]. Medical professionals in Taiwan are often required to attend social events sponsored by their supervisors. This poses a challenge for them to resist alcohol provocation, as such an attitude might be perceived as an offense to their supervisors. A competitive but unfair drinking rule encouraged by alcohol provocation also reflects the impact of workplace hierarchies. One study pointed out a common situation in Chinese drinking culture where it is not acceptable for subordinates to drink less alcohol than their supervisors [33]. This was also demonstrated in our study where residents involved in unfair drinking under the influence of hierarchy were compelled to out-drink their supervisors. The behavior of alcohol provocation associated with superiors is related to a manifestation of power or superiority by forcing subordinates to drink [34, 37–39]. Consistent with the findings from Asian workplaces [34, 37–39], our study also showed medical professionals with a higher position in the hierarchy are prone to force those in inferior positions to drink more. Workplace hierarchy is also apparent in specialties where a relationship of mentor-apprentice is reinforced, such as surgery and orthopedics [40]. This may explain why a few participants in our study had reported a more frequent behavior of alcohol provocation particularly associated with specialties where the existing superior-subordinate relationship is more prominent.

Distress is likely to be caused in participants experiencing alcohol provocation either from colleagues or superiors. The pressure comes from conformity to cultural and workplace norms or fear of jeopardizing their career if failing to comply. In fact, findings from two studies have revealed that workplace-related drinking events are viewed by some workers more as a job obligation than a social pleasure [31, 34]. Although reluctance to attend to these events wasn’t obviously observed in our study, perceived pressure from alcohol provocation was definitely expressed by the participants in our study. Moreover, our participants mentioned the experience of alcohol provocation may cause them to develop a negative impression of those who engage in such behavior. This implies that there might be a deleterious effect on interpersonal relationships with colleagues who consider such behavior unacceptable and a potential threat to their workplace wellbeing. One study examining problem drinking among physicians also suggested that interpersonal relationships characterised by abuse or harassment may be an additional source of work stress to the victims [41]. Unquestionably, medical professionals being pressured into drinking at gatherings may have an impact on their medical practice and workplace wellbeing. In accordance with a previous study on alcohol provocation among students [1], a similar influence of alcohol provocation on medical professionals including hospitalization and loss of consciousness, was also revealed in our study. Multiple articles have addressed issues of impairment at work as a result from voluntary alcohol use among physicians [42, 43]. In our study, with involuntary alcohol consumption initiated by provocation, our participants were often unprepared to endure the consequences of forced drinking. Although compromised work performance is also described in our study, some medical professionals had never expected to deal with absence from work or low punctuality. In the long run, these medical professionals will have to face potential adverse outcomes such as failure to maintain professional identities or provide proper patient care.

Our findings suggest that the adverse situation associated with the drinking behavior of medical professionals at gatherings is improving accordingly as the drinking culture in Taiwan is gradually transformed. Those medical professionals of the younger generation who disapprove of alcohol provocation are more able to cease participating in this behavior than those of the previous generation. These professionals from different generations present distinct perspectives on alcohol drinking and alcohol-related provocation. Aspects of drinking culture which may evolve throughout an organization or be limited to a particular generation are possibly attributed to social transition, as described in another study [44]. The prevailing views of personal rights and self-awareness in the current society might also act as a driving force for lowering the incidence of alcohol provocation.

The cultural belief of drinking traditions as part of workplace culture, such as dominant–subordinate professional relations, can be tied up with the role of social control [19], which may encourage the behavior of alcohol provocation associated with medical professionals. In addition to year-end parties which are regular annual events for both physicians and residents at workplaces, residents also have to participate in other drinking events such as welcoming ceremonies and mentor-support gatherings. This may increase the likelihood of experiencing alcohol provocation and provide an explanation for the residents having a significantly higher monthly alcohol consumption than the physicians in our study. This suggests that alcohol provocation still exists in the workplace and is an important issue to be acknowledged. While the issue of drinking behaviour similar to alcohol provocation was discussed in previous studies, it was found only in fraternities and sororities in universities [45, 46], and the cause of forced drinking reported in these studies was mainly attributed to peer pressure [1, 45, 46]. Currently, no other study specifically addresses alcohol provocation among medical professionals participating in compulsory social events, in particular, such behavior that is subject to cultural differences and social expectations.

## Limitations

The participants we recruited came from a single hospital only. It is expected that regional differences in drinking culture might exist in hospitals in other locations. However, this limitation could be possibly minimized, as all the participants graduated and received training from schools in various locations across the county. Further studies with future participants of diverse backgrounds may provide a better insight into alcohol provocation in Taiwanese health professions.

## Conclusions

We discussed the relatively understudied topic of alcohol provocation that may coexist with underlying factors of specific cultural norms, workplace hierarchy and social expectations. It is important to understand how alcohol provocation occurs in the medical field for early prevention of alcohol abuse among medical professionals in Taiwan. Our findings can help increase the awareness of adverse consequences associated with alcohol provocation, encourage medical professionals to avoid inappropriate drinking behaviors, and reduce the risk of compromising medical professionalism.

## Supporting information

S1 File A topic guide for focus groups. (PDF)

S2 File Transcripts for a total of 12 focus groups involving residents and physicians. (PDF)
