# Correction: Influence of alcohol provocation on medical professionals in Taiwan: A qualitative study

**DOI:** 10.1371/journal.pone.0300940

**Published:** 2024-03-14

**Authors:** Ching-Yi Lee, Ching-Hsin Lee, Hung-Yi Lai, Mi-Mi Chen

The second affiliation of the third author is not indicated. Hung-Yi Lai is also affiliated with: College of Medicine, Chang Gung University, Taoyuan, Taiwan.
