# Supplementary material for: Influence of alcohol provocation on medical professionals in Taiwan: A qualitative study
Source: PLoS One. 2022 Feb 16;17(2):e0264071. doi: 10.1371/journal.pone.0264071 (PMC8849514; doi:10.1371/journal.pone.0264071)
Supplement: S1 File — (PDF) [file pone.0264071.s001.pdf]

S1. SEMI-STRUCTURED INTERVIEW QUESTIONS

1. Can you list examples of circumstances where you would have meals with alcoholic beverages?
2. Have you attended any Weiya (year-end drinking parties) or dinner gathering held by the department? If yes, describe your alcohol-drinking experience at these events and how does it affect you? If no, describe any similar drinking occasions at your workplace you have experienced? (prompt eg. Drinking together with the supervisor and other colleagues and the purpose for such event is mainly work-related.)
3. How does the work-related purpose for such drinking events make you feel? (prompt eg. Do you enjoy or dislike the drinking atmosphere at these events?)
4. What is it like to drink with your supervisor or colleagues at these events? Alternatively, is there someone you know from the workplace whose drinking behaviour has affected you or others in any way?
5. Would you organize a Weiya event without any alcohol involved? Why or why not?
