# Supplementary material for: Influence of alcohol provocation on medical professionals in Taiwan: A qualitative study
Source: PLoS One. 2022 Feb 16;17(2):e0264071. doi: 10.1371/journal.pone.0264071 (PMC8849514; doi:10.1371/journal.pone.0264071)
Supplement: S2 File — (PDF) [file pone.0264071.s002.pdf]

Group 1

Residents (n=6)

Group 1

Interviewer: Please introduce yourself including your position title, hobbies and favorite food.

1: A resident. I like reading novels, outdoor activities and wouldn't say no to any sport. I prefer street food and eat all kinds of food found in the night market, whatever I feel like eating and not particularly limited to a certain cuisine.

2: I am currently a resident. Travelling is my hobby. I love traditional Taiwanese food and noodles or anything similar from the northern cuisine. I enjoy trying all kinds of food.

3: I am also studying my residency. I love board games or reading novels, movies and prefer outdoor. I like all sorts of ball games. I also like to travel. My favorite food are noodles and meat.

4: Hi, everyone. I am also a residency student. My hobby is reading poetry and prose. Watching some short films is also what I like and makes me think about the possibility of creating my own films, films about activities. About the food, I prefer light food to meat, and that's all from me.

5: I am [in] residency too. I like gardening and aromatherapy. My favorite food are sashimi and cakes.

6: I am also a residency student. My favorite food are noodles, steam buns and anything similar to these. I have an interest in learning all types of alcohol such as Belgium beer of all kinds, red wine and its place of origin, and all the different flavors of whiskey.

Interviewer: It seems everyone likes noodle-based food. I would like to know if any of you has a habit of drinking alcohol with your meals?

1: It depends. Yeah.

Interviewer: Would you please tell us about the frequency and occasion?

1: Such as Friday nights, with your close friends. The atmosphere of the restaurant such as Le Ble D'or, American-style restaurants or others with a better [drinking] atmosphere that is suitable to have a cup of beer or something.

Interviewer: Do you go every Friday then?

1: About once a month.

4: Is this about the way of having a dinner gathering?

Interviewer: It's about whether or not you drink normally with your meals and how often you do that.

4: Not very much unless I am in a bad mood when I need to loosen up. Or when [eating] with elders, eating with them during Chinese New Year or special holidays, everyone would open some bottles of alcohol, wine or beer. It's also likely to drink with friends whom we only meet up once in a while at a restaurant to have some pizza or American food. This however doesn't happen that often.

Interviewer: Would this be about once a month?

4: Sometimes not even once a month, probably once every 2 months.

6: I used to have wine every time with my meal when I was in Europe before. I stopped once I found out I gained weight too much because of it. I don't drink if I am eating alone as it's not so convenient to go out and get wine. I only

buy alcohol to drink if I am in a good mood eating with my friends. I have made some drinking friends recently.

Interviewer: On what occasion?

6: There is a winery, Macallan who has been offering free beer recently. Lots of free [beer]. Drinking brings some good mood and makes it easy to meet new people and make some friends. I would take wine to meals.

Interviewer: It seems to happen frequently.

6: It has been quite frequent these days, yeah. I had 4 days of drinking in one week. It was 2 days [of drinking] last week. I guess it will be 2 days this week.

2: I drink about 1-2 times a month. I drink when my mom buys the [Taiwanese] fried chicken, or sometimes when I eat with my friends, or when my friends who are in a bad mood would take alcohol to me. This is about 1-2 times a month.

5: We have opened bottles of wines on the table at our home. I will take some whenever I see them. When I eat out with my friends, it depends on the people I eat out with and there is always this one or two groups of people I go out with.

Interviewer: Do you drink a lot at home?

5: It's only to have a taste. I don't consider it as drinking.

Interviewer: What about eating out with your friends?

5: There is always this one or two groups of people I usually eat out with.

Interviewer: Where is that and how often do you drink?

5: About once every month or 2 months. The place would be KTV or a restaurant.

Interviewer: Would anyone have any experience of a drinking event at a divisional gathering, year-end parties or spring receptions?

2: I did once go with the teachers and other invited students from one division before. We would go to a bar or any place where you could eat and drink alcohol.

Interviewer: There were teachers and a group of students.

2: Yes, teachers and a group of students.

1: There was a year-end party every year held at a cram school I used to go to during high school. All the superintendents from the ministry of health and welfare were invited to eat with us. The superintendents of hospitals. It's a strange organization but it's still there. There were lots of tables of the students of the supervisors form the hospitals, and 2-3 tables of all the supervisors. There were occasions where we had to propose a toast with red wine.

Interviewer: Did you say a cram school?

1: It was a cram school I went to during high school. I know it sounds strange to have all the superintendents from the ministry of health and welfare and supervisors from all hospitals.

Interviewer: Any other experience from eating at a divisional gathering?

3: When I was in the alternative military service in Hualien, we had drinking events with other soldiers who were also from other towns [like me] and our supervisors during Chinese New Year or holidays.
Interviewer: How often would you drink?
3: I think [it's] about once every two months.

Interviewer: Shall we share stories about work-related drinking occasions and what the situation was like?
5: Doctors from mainland China came for a visit. The ward chief physician of the division who happened to be my supervisor arranged a dinner gathering with them and I was there too. I was being the lowest rank at that time. The chief physician kept pushing me to drink. I did keep on drinking with few others of the lowest rank as to avoid the situation turning awkward. In the end, we filled the room with an entire row of empty bottles at Shang Jie Qing the restaurant that serves stir-fried dishes.
Interviewer: With the chief physician you said? And where was that? An entire row of alcohol? Were there any others except for the chief physician?
5: There were 2 tables at that time. One table was for the chief physician, seniors and mainland China doctors while another was for the nurses.
Interviewer: There were nurses, chief physician and seniors.
Interviewer: Would you care to describe what the situation was like at that time?
5: The nurses were shy. One or two nurses had come [to our table] to make the dinner gathering more interactive. The China guests there were shy and the chief physician would ask us to add more excitement to the situation. We helped do so by drinking.
Interviewer: How would you bring that excitement?
5: The chief physician would say "bottoms up" to you. He drank throughout the entire dinner.
Interviewer: It's the doctors from mainland China?
5: Yeah. The chief physician kept drinking. When we only drank half, he [the chief physician] would mutter "hey.." or anything in disapproval, but say something like "this is how you do it" once if we would finish our drink.
Interviewer: I see. There was no turning down.
5: Yup! It wouldn't be good if we also turned down the drinking offer like the guests did consistently.
Interviewer: Anything related to work, work at hospitals or during internship.
1: When I was a clerk doing an easy pediatric rotation and it was good enough as long as you were present there. The senior interns who were close to you would invite you to a dinner gathering. Once I had a dinner gathering with the senior interns, one senior made it clear that it's not ok to leave without drinking any alcohol. At that time, one of my colleagues and I had to be on duty while those seniors already got off work. It's not important if a clerk had to be on duty. The seniors would open bottles of wine. We would say that we had to be on duty when they were about to open the bottles. They would say something like " Geez, what on freaking duty?! Who is your senior in charge? I will call them up." So they did make the phone call and we could get to stay

there and drink. We didn't have to get back early as the attendance check wouldn't start until 9. We finished dinner around 8 and our seniors caught us drinking too little when we were about to leave. They would demand us to finish a whole bottle before leaving and we did so before they would allow us to leave.

Interviewer: This happened while you were on duty?

1: Yes. We had nothing to do while on duty. We weren't responsible for anything while on duty.

Interviewer: Was this in the hospital?

1: It was a nearby restaurant, Han Zi Wei. We were able to drink because everyone had no responsibility to bear. Yeah, it might not be a good thing but it's funny.

Interviewer: Would you tell us more about what it was like at that time?

1: It was a great atmosphere. People would have to rotate and leave the round after the internship. It was nice to have this internship dinner gathering with our seniors who were also our friends. The seniors would invite us to this farewell gathering and they could get very hyper. We were friends with them and we had to drink with them if they bought alcohol. They would ask you to drink for reasons of doing anything. Say if you are good-looking then you must drink to that. Drink to any circumstance. Everyone was getting all excited. Once you had drunk to a certain amount you would drink to any reason. This is how the situation was like.

Interviewer: The seniors would invite you juniors.

1: We didn't know we would have to drink at the start. We only thought about having Korean BBQ at Han Zi Wei. To our surprise, the senior bought lots of alcohol and the expense was all on him. He told us to drink and said it was no fun without drinking. The senior seemed to enjoy drinking alcohol.

Interviewer: You would think you were invited by your senior only to a dinner gathering, only to find out it wasn't so when you got there.

1: It was possible that the senior didn't intend to drink at the start until he saw the alcohol.

6: I hadn't been to any hospital work-related occasion where I had to drink. Neither did I have the drinking experience with colleagues, seniors or teachers. I only had drinks with my colleagues and boss from tutoring work.

Interviewer: A tutoring work currently?

6: On one day when it was a mid-autumn festival where a celebration was needed. A bottle of wine was brought to us, we then started drinking and doing a lesson together at the same time. Half way through the lesson, he told me that he couldn't continue the lesson as his brain couldn't work anymore. I did make less money on that day. Then he started working and I would take him to a bar if he wasn't in the mood for a lesson because of having a bad day. It was like an out-door learning experience. I was teaching Chinese and he would be able to learn speaking [in Chinese]. He would order vodka for himself while whisky for me. We had few lessons or lessons pretending to be

an out-door learning experience. He would even invite me to the farewell party for his colleagues.

Interviewer: You would invite your students to drink with you at a bar?

6: Once or twice. It’s because he was in a bad mood.

Interviewer: Any others had drinking experience with your seniors or elders?

2: It was also a gathering during internship. An intern’s place where few clerks were invited to cook and have some chat, there was cocktail provided.

Interviewer: It was at the place of a senior intern?

2: There were senior and junior interns.

Interviewer: About how many times a month?

2: About once every month or 2 months. They would invite me occasionally.

Interviewer: Would you think differently about a person after you see them drinking at a gathering?

1: Yeah, I would. There is this senior who is usually quiet but turns into a terrible person after drinking. He would grab your shoulder in any way he could. I think alcohol has the power to change [a person]. One senior who usually appears upright and clean shows a desperate desire for drinking after taking alcohol. I would see him normally as a shy and upright person and he would seem pretty different after he has taken alcohol. There is a big difference.

Interviewer: You would view him differently from the way you normally see him.

1: I would then realize he is a person shy on the outside but wild on the inside because I have seen him drinking.

Interviewer: It’s like a contrast.

1: Yeah. I think alcohol can reveal the true personality of a person.

5: I have a drinking buddy who is a nurse. Her roommate would kiss everyone around her when she gets drunk. It means that she would sleep on the laps of many young hunks. She would show some strange behavior and I find it terrible after hearing things about her.

Interviewer: You would change your views about them?

5: I am a little surprised by the story about the person who is usually decent. So there is a different side of him but he is ok at work.

Interviewer: Do you know him as well?

2: Drinking makes you become closer to others. What he said earlier about how you would open up more. You would also get along more naturally with friends who have taken alcohol. You would be closer to them and avoid the feeling of being uneasy at the start.

Interviewer: Would you say you think positively about the person who drinks?

1&6 agree with what 2 said.

4: You have to know them well enough to be closer to them. If you drink with a person who you are not close to you might find it awkward seeing them the next time. One time after the morning outpatient work finished at 1pm, I was asked to eat out by 2 ladies from the pharmaceutical companies, we went together to a brewpub in an alley. There was a distinct atmosphere there. Every customer seemed to know the owner well. Everyone was leaning

against the bar with a pot of sake in front of them. There was a cup for everyone who had to keep on drinking. We were drinking non-stop with the people from the pharmaceutical companies, teachers and seniors. Alcohol seems to be a connector for all relationships and languages. It makes you talk differently and boldly. Without the effect of alcohol, you would think it’s not appropriate to call a teacher without addressing them, even if the teacher themselves find it weird if we do address them as teachers. You would find it hard to address or talk to a teacher whom you are not close to. A distant relationship.

Interviewer: Will you change the way you see a person whom you have witnessed them drinking?

1: Don’t drink with those whom you are not close to. Yeah. Don’t go with [people of] different ranks and never drink with them [people of different ranks]. It’s a respect.

4: It’s not likely to drink with someone with a different rank. Once I did drink with such person, I had to control my speed of drinking and avoid drinking too much. He got a call as he was on duty that day. He returned to the hospital and then came back to the dinner gathering afterwards. I still think it was not appropriate [to drink with such person].

Interviewer: It was not appropriate because he was like an elder to you.

1: I feel that it would be hard to make them drink with you because of them being in a higher rank. It depends on the difference in the position rank at work. It’s easy to make friends with interns. There will be pressure to drink with visiting staff and supervisors and you would avoid drinking with them.

Interviewer: What do you think about the drinking behavior? What’s the meaning behind it?

6: It depends on the type of alcohol. You can get hyper and noisy while drinking beer at a restaurant that serves fried dishes. You feel like being in a upper class while drinking red wine, whiskey and anything similar that is usually more expensive at an event like the clinical mentor gathering.

3: People will loosen up and be less vigilant after drinking. Alcohol makes you feel easier to be around with a certain person whom you normally might be careful to show a lot of respect to. It also makes you talk easily to a friend whom you normally wouldn’t dare talk to. It is like a catalyst helps build a closer friendship. (4 nods to show agreement)

Interviewer: You reckon it is a good behavior which helps a closer bond with friends.

3: [There is]No behavior change but with a clear mind. I think it’s a positive thing.

1: I had been to one [event] but not in the hospital. I was on a visit to China, it was an academic exchange for students. China and Taiwan were opposing each other at that time. We drank carefully to defend [ourselves] and tried to prevent our supervisors from being pushed to drink till drunk. This drinking behavior is also related to high rank status. We drank white wine. You would try to outdrink others at the table in order to make some deal. It was like to have a gambling game at the table. You had to make your move, defend and

negotiate. They said drinking makes you promise something that you wouldn’t normally say yes to when you are sober. You are more likely to give in when you are drunk. You feel happy in a great atmosphere like that and you would promise things easily. People from pharmaceutical companies will buy doctors drink not only for building a closer relationship but also hoping to get some feedback or benefits.

Interviewer: Drinking behavior is thought to have some negotiation involved as well.

1: Negotiation might be involved but the main purpose is to build a closer bond. Drinking makes it easy to make a promise in a deal.

Interviewer: The part of making a deal is involved.

Interviewer: What does it mean to you personally?

5: I think it does harm to your body. A glass of gaoliang has to be finished upon entering a dinner gathering with the senior nurses.

Interviewer: As for you, it would do harm to your body.

2: I drink occasionally but I don’t fancy drinking alcohol, neither do I feel relaxed after drinking. Lots of people choose to drink when they feel down. I wouldn’t do so when I am in a bad mood. I only drink for adding more excitement or social purposes. It’s no harm to drink occasionally for social purposes. I wouldn’t drink frequently though. I would control myself and avoid getting drunk when I drink.

Interviewer: For you it’s for social purposes.

Interviewer: Do you arrange a drinking event if you are to hold some gathering activities?

1: It depends. Back in the time when people were more conservative, they would prepare alcohol for a certain doctor, if knowing this doctor who liked to drink. On the contrary, it wouldn’t be appropriate to do so if people are only there for something to eat or not in the mood. It depends on the situation. If everyone agrees to have no drink, no bottle of wine would be opened when everyone gathers happily to eat.

4: It’s not necessary. There is always an option. You may drink when you feel like it.

1: It depends on the person you are drinking with. 6 drinks if you invite 6.

6: I drink depending on the event.

4: Yes, I would prepare alcohol. I would do so, say if I am holding a gathering or something today, after work or a weekend gathering of some sort. I definitely won’t force people to drink, [there will be]no behavior of urging others to drink unless they want to drink.

5: It depends on your guests if you are the host of the event. Some would demand to have some alcohol to drink and some wouldn’t necessarily drink. You have to make them sit separately to avoid awkward moments.

6: It is to see if anyone is up for it[drinking]. You wouldn’t drink if you don’t usually drink.

1: I know I will have to prepare some alcohol [points to 6] if I get to invite you.

6: It depends on the people I am eating with, see if they drink.

Interviewer: Would drinking at an event influence medical practice?

1: It’s unlikely most of the time if you only drink on the day before you have a day off the next day.
4: When I was a clerk, one of my seniors had a hangover from drinking the day before the previous day and he couldn’t go to work at all the next day. I felt that everyone was talking about him in the morning on that day at the operating room. You should drink moderately on the evening before the day when you have some schedule to run. Everyone would know what’s going on.
Interviewer: You think it has an influence on the senior who was supposed to be on duty.
4: Definitely.
6: I think there is a huge influence. Drinking too much till I drop on the day before [work] and being late for the next day. I was feeling dizzy without knowing what to do at the operating room. It greatly affected my brain, learning ability or anything I did so it is necessary to control what you drink the day before [work].
Interviewer: Dr. Lin, you think you would be personally affected.
2: I have never been drinking and gotten so drunk even the next day. I do agree with what everyone said. You would limit the amount you drink if you have a schedule to run the next day.
Interviewer: You personally think this won’t have an effect on your behavior?

Interviewer: If one day you have become a supervisor would you keep it the same way? to have something similar to what they did in the past for the drinking gathering?
2: I think I will still drink with everyone but I won’t force others to drink. For others, it will be a stress. I won’t force them to drink and everyone is free to make a choice.
Interviewer: You won’t do it in the same way as what others did before.
2: I won’t force my juniors to finish their drinks.
3: If I have ever become a supervisor I would still invite them[juniors] to drink at an event similar to what was mentioned earlier. It’s important to have a good observation being a visiting staff. If someone has been unwell after drinking you wouldn’t encourage them to drink. If a person is a bit unhappy or only drinks a little you wouldn’t invite them or urge them to drink more. Alcohol makes everyone closer to each other but never force others to drink if you have observed them being sick or having any discomfort.
Interviewer: You also wouldn’t force others.
1: I might arrange [this kind of drinking event]. I think this is nothing to do with forcing others or not. It’s more like depending on what the situation is like. The visiting staff wouldn’t force him… but he….
3: It doesn’t matter. This is also considered as forcing others or urging others to drink.
5: The chief physician is pretty active and he would urge others a lot to drink while everyone is quietly having their meal and this makes a big contrast in the scene. He [the chief physician] seems to be one of those who is very good at socializing.
Interviewer: The chief physician would keep urging others to drink.

5: I guess he is one of the special ones. For me, it depends on the occasion. I drink sweet wine, which is similar to what girls drink, at divisional gathering. I still drink. A mild alcohol helps bring excitement but you don’t have to drink if you don’t want to.

Interviewer: Will you urge others to drink if you have become a supervisor?

5: No, I won’t. I don’t like doing so.

6: I think it depends on the type of restaurant you go to. There is always beer provided at the restaurant serving fried dishes. I never urge others to drink. You pour yourself some alcohol if you feel like it or else you could always grab some juice. However, it might be a different story if I get too hyper in drinking and become hard to control.

Interviewer: What else do you think?

1: The atmosphere would make you drink. Even there is no one forcing you to drink, it’s hard to be the only one sitting there without drinking when everyone else is drinking. It depends on what the situation is like. It’s hard to avoid drinking when there might be a pressure from knowing everyone but you is drinking.

3: There should always be other drinks than alcohol provided at a drinking event. Other drinks can be an option to avoid awkward moments.

6: Apple cider is a good choice.

1: It has got something to do with manners if an optional drink is provided. You can’t secretly replace your drinks. There are still occasions where drinking alcohol is a must.

5: It also depends on the intentions. If we are some business people making a deal with customers I would take people who drink to the event. We will definitely drink for doing the business. Otherwise there is a risk of losing your deal without taking people who drink with you.

Interviewer: Has anyone heard or been in this kind of situation?

5: I have this one friend who is doing some trading business. He would say something like he has a hangover at 10 or 11pm and complains about him having a hangover the next day. He has to go to all kinds of drinking events related to work or for social purposes. He said he can’t do anything about it. It’s for the job. If you eat with him you will find him being capable of drinking a lot. One drink after another and from morning to night, he would drink beer like water at every event he goes to. His boss likes to take him to any business dealing occasion.

Interviewer: Anything missing or you would like to add?

1: I think it’s still acceptable for hospital staff or doctors to drink provided that what they do will not affect a patient’s safety. You may turn to your colleagues for help but never carry out medical practice [under the influence of alcohol]. From a hospital’s point of view, drinking brings relaxation to people and should have no effect on your work. Drinking is a personal behavior and has got nothing to do with others.

Interviewer: That is to say never force yourself to drink at a gathering, but what if you are being forced by others?

1: I agree with never force yourself to drink. You can’t help it if you happen to drink with someone in a higher position than you. There is a pressure from positional rank. You may try to control [drinking]. One drink for you and one for me. We are those of a lower rank drinking with someone who urges us to drink and if we are able to make this person drunk, it means this person has shown us some respect. We should never be affected by others saying to finish the whole glass. There is a lot of us being in the low rank. A person who urges you to drink is made drunk by you. They respect you if they drink a glass with you.

5: If you don’t want to drink, one of my seniors would drink tea instead of alcohol or make up some excuses. Saying something like she has to drive so she would drink tea instead of alcohol, otherwise it’s risky [to drive].

4: Those who urge others to drink would drink heaps and they can get really hyper. It is awkward to ask them to go easy on drinking. It is [considered] to spoil the moment or considered to be shy if you don’t drink. They will still push you to drink. It’s not easy to turn down [the drinking offer]. The person who urges you to drink is not acting in a way you used to know and they can be difficult to communicate with [under the influence of alcohol].

2: I think it is ok for a doctor who usually drinks a little. A way to train your alcohol tolerance as long as your work is not influenced by alcohol. It is necessary to drink occasionally.

Interviewer: You meant to have a better alcohol tolerance for coping with situations when being urged to drink a lot.

5: It’s hard to avoid people making a toast to you.

3: For me, if I know I would be on duty to work the next day I will figure out how much I should drink, knowing that drinking too much does no good to my body and I will only drink up to the amount which still enables me to work normally the next day.

Interviewer: So what happens if you have to work the next day?

3: Limit the amount we take and understand our own physical wellness.

Group 2

Physicians (n=2) & Residents (n=3)

Group 2

Interviewer: Please introduce yourself including your job position, interests and favorite food.
3: A resident. I like red bean tangyuan soup and my hobby is playing video games.
2: I am a resident. My interests include reading novels and playing video games. No particular type of food that I like, but probably the hot pot or anything similar.
1: A resident and I like to watch drama series. Anything sweet is my favorite.
5: Neurosurgery. My only hobby currently is sleeping. About the food I like, I have been fond of strawberries recently.
4: I am in neurosurgery. My hobbies were used to be novels and movies but I found them inconvenient. I think it is more convenient to play mobile games or I would sleep in my free time. No food in particular I like. There is none. I only like anything that is tasty. I decide if they are tasty or not.

Interviewer: Does everyone usually drink alcohol with your meals?
5: It depends on the people you are going out with.
3: Mostly no, only occasionally.
1: Yeah, most of the time.
2: Not so much, hardly any unless it’s for some special occasion such as occasionally having a small glass of alcohol at a restaurant that serves fried dishes.
Interviewer: At a restaurant that serves fried dishes, how often is that?
2: About once every several months.
4: You would drink whenever you go to a restaurant that serves fried dishes or drink only when there is someone drinking with you.
2: I wouldn’t drink on my own initiative. I might possibly drink if someone brings alcohol to me. It depends on my mood of the day. I might choose to drink juice on that day.
4: I usually don’t drink on my own initiative. I drink only when I eat with my wife who would ask for alcohol. In this case I am more than happy to drink.
Interviewer: How often is this?
4: It’s not that frequent, only once every several months. It’s more likely to drink in a dinner gathering with friends who also show an interest in drinking. It depends on whether or not you are driving on that day. I will never ask to drink on my own initiative.
Interviewer: It is once every several months.
4: Mostly. It’s more likely at dinner gatherings. I have been too busy to go to any gathering recently. It is more like to drink once every 2 to 3 months to be exact.
5: I hadn’t been drinking for quite a long time before. I couldn’t drink because I was expecting a child. There was hardly any drinking gathering compared to now. I eat out and drink with my friends possibly every month now. I have started to drink a little with friends recently.

Group 2

Interviewer: Do you have any drinking experience from like divisional gatherings, year-end parties or spring receptions. Would you share with us what it was like?
4: Yes, every year.
2: I only had once so far.
1: I would drink at a gathering where there is alcohol provided otherwise I wouldn’t.
Interviewer: Could you tell us more about what the situation was like?
1: We would drink and chat and possibly proposed a toast to each other. Everyone had to drink if we were making a toast to the elders.
Interviewer: Did this happen at your divisional gathering?
1: No, but the spring receptions for interns and there were some teachers present.
Interviewer: Would you make a toast to your colleagues?
1: The teachers would go table by table to make a toast to you.
5: Did you drink first before or when they came to you.
4: Was there alcohol for every table?
1: Yeah, we would drink first and normally we would drink first at an occasion like that but mostly it was because the teachers were also drinking. Some teachers were really capable of drinking a lot.

Interviewer: Who were those people you were drinking with?
1: It could be anyone, colleagues and teachers.
5: Talk about what happened then?
1: A table of students started drinking a little first and drinking continues again as the teachers showed up halfway through the event.
1: We wouldn’t drink too much.
5: You didn’t have too much alcohol. It was ok.
1: Because there wasn’t much [alcohol] provided.
Interviewer: Could you describe what the atmosphere was like?
1: Atmosphere. A group of people with alcohol showed up at our table and we would all stand up to congratulate [them].
5: What sort of alcohol did you drink at that time?
1: Red wine.
5: No wonder the alcohol wasn’t supplied consistently.
4: There was a limit for each table. People were not likely to get drunk. Only one bottle for each table. Everyone would only have a little to drink.
Interviewer: Would other colleagues..?
1: Would they drink when on duty?
Interviewer: Yes.
1: Yeah they would.
Interviewer: You couldn’t have a lot to drink because alcohol provided was limited.
1: Yes, it was limited.
5: You may talk about the events you had been to previously.
3: At those ones I had been to, it was usually the people who came to our table first to make a toast and I normally wouldn’t have any to drink, or just few sips to the most. I had the non-alcoholic drink when people came to make a toast to me. My face turns red very easily [after drinking] so I seldom drink.

I used to attend the events on behalf of someone else. Mostly the people who came to make a toast to me were not my colleagues at work. I took other non-alcoholic drinks instead if I had to make a toast.

Interviewer: The colleagues would make a toast to each other all the time?

3: Not all the time. They would only do it once for each gathering and showed their etiquette. They would also make a toast at other non-work related occasions.

2: It’s more likely to drink a toast when going out with colleagues or coworkers but hardly any with the elders. It’s like going to a wedding event, similar to what 1 had described. Everyone would chat and drink a little at their table, go to other tables to make a toast or people from other tables would come. It was a similar experience to what I had before in the club and society. I wouldn’t drink much but only one or two glasses. (Touches head)

Interviewer: Was this the school club? It’s ok to share the experience with us.

2: Just like what 1 had said. Let’s be realistic, you wouldn’t meet a senior who is really considered to be the eldest at a school and they are not old enough to make you drink. An occasion held by the choir club is usually quite peaceful. People would just have some chat. Some people who liked to drink would take one or two bottles. One to two cans of beer for each table and people were free to take it if they wanted. There was also barley tea or juice provided for those who didn’t drink alcohol. It was free and no one was forced into drinking. About making a toast at that event, it didn’t have to be alcohol, people could just say hello or greet in general.

Interviewer: There was nothing like urging others to drink?

2: There wasn’t anything like that at the events I used to go to.

Interviewer: Has the rest of you two been to anything like that?

4: I had been to several big events where I would take alcohol most of the time as there was always alcohol provided. The drinking situation depends on the participants of that division. Some host of a major surgery division. There is always a dinner gathering held after the national conference of neurosurgery society, some host of a higher positional rank would drink alcohol in an extreme way. Everyone would take alcohol extremely at the event and force the lower ranks to drink. Everyone took a round, one glass for you and one glass for me, everyone could end up drinking more than they should have. It depends on whether the doctors are willing to drink or not. Basically, I would keep drinking my tea and ignoring each of the teachers coming from the same table to make a toast to me. I then would refuse to attend any of those events. If I had to be there I would just drink tea and they had to leave me be. People around you might find you weird but you only had to insist that you don’t drink. We had some occasions where alcohol was provided and you could drink as much as you like. Nobody would have any problem of you drinking to get drunk nor would they force you to drink. It’s common in Taiwan to make a toast to each other and you will always end up having a little to drink. You were free to drink juice if you wanted at those events and no one would force you. In this way, not many people were likely to drink too much and get drunk. Most people were not into drinking too much anyway. It was not

common to see lots of people getting drunk at those events, but it was more likely to see people getting drunk or even throwing up at the event I mentioned at the beginning.

Interviewer: It was the teachers you were drinking with?

4: It depends on the occasion. For the occasion mentioned earlier, it has got to be the teachers we have to drink with.

Interviewer: Tell us about an unforgettable event.

4: I hadn’t been to many and there wasn’t any unforgettable one as I recall. A teacher would come to me with a bottle of wine asking how many drinks I would drink in response to his drinking toast in one glass. He would say it’s a must to outdrink your teachers. Some people drank until they threw up. I didn’t take it seriously as this teacher didn’t know me at all. He wouldn’t remember me once if he got drunk so I kept drinking my tea. The color of tea looked like that of alcohol anyway. I had been to the society gathering only once and I had seen some extreme drinking going on there. Once at one gathering for the surgery society, the superintendent came to make a toast to you, you had to drink 5 glasses while he only had to drink one. You would rather choose to stay away from those events and you could make your own decision without being forced to attend. Don’t go if you don’t feel like it. You are more likely to drink more with best friends and it is more interesting as everyone would prepare some better wine to drink. Alcohol provided at those occasions is usually not so tasty and you wouldn’t want to drink it.

5: Once there was a society gathering where everyone had to go to the stage to make a self-introduction. Everyone was required to drink 5 glasses before getting off the stage after the introduction. Most people weren’t aware of this rule until they were told. I managed to skip it as I told them I was pregnant. Those elders and supervisors would demand a person to drink 5 glasses and this person would have to do so.

5: I remember when I was younger attending a gathering held by neurosurgery, I heard afterwards about people ending up in emergency room to have an IV drip. It’s now rare to see people drinking too much or throwing up as compared to before when people would drink until they couldn’t even make it to the toilet to throw up.

3: I remember he said something about a person who was so drunk and he fell on his back while kicking to show people that he knows some Taekwondo moves.

Interviewer: What occasion was that?

5: At the year-end party or the gathering after society conference.

Interviewer: What was the atmosphere like?

5: There were 2 kinds of people. The kind of those who love to drink and these people would pretend to be drunk and act ridiculously. They would voluntarily invite you to have one drink while they have 3. You have one drink and they will have 5 or they will say something similar to the teacher too. They who enjoy drinking would purposely make some complaints about something but in fact it’s only an excuse for them to drink. In addition, this

kind of event is always work-related, I find it weird and also hate a situation like that.

4: It could be disgusting sometimes. It’s weird to be bros with and hug someone who is normally your sworn enemy after drinking. You know those people who don’t get along well with others in the division but it’s awkward they seem to be ok with each other while drinking. Say if he is your teacher who is usually serious and very strict to you but shows a different side of him after drinking. They seem strange when drunk. They would control the amount they drink in the public and only drink a little out of politeness. I think it’s also weird to make a toast to someone whom you never want to talk to. It’s strange if you so. What is more embarrassing is that you have to lead the residents to make a toast when you are a senior CR. I don’t like this [drinking] culture and it’s weird if I have to take the juniors to drink even when I hate it. I am more willing to attend the divisional gathering where the custom has been changed to allow people to drink whatever they want. It’s more natural and you are free to choose juice instead of alcohol.

5: We also drink a lot at our division. It’s weird for sure to lead the juniors.

4: It’s also free for us to drink whatever we like and we won’t be forced to drink. People are willing to go purely for drinking if there is good alcohol provided and the drinking behavior doesn’t matter that much.

Interviewer: Have you seen VS binge drinking at the spring receptions for interns?

1: They actually didn’t drink that much.

4: There wasn’t too much alcohol.

5: You didn’t find anything unusual?

1: It was ok.

5: They really didn’t drink that much. I remember we used to drink quite a lot during the year of our internship. We drank until the second or third dish was served then we would start singing.

Interviewer: Any work-related drinking experience after work either with your colleagues or seniors?

3: NO.

2: We usually drink when we eat out with friends and there is no pressure to drink alcohol. We are free to drink whatever we like. It’s ok even if you don’t drink alcohol.

Interviewer: Would you care to tell us more about what it was like?

2: We would sit together to have some chat at any place possible including the dormitory. Some would prepare alcohol while some get some fried chicken. They are all colleagues and classmates.

1: Peers.

Interviewer: Would drinking at a gathering affect the way you see a person whom you usually spend time with?

1: It should be ok. You know when he is drunk.

5: You wouldn’t change your views about peers but elders. Let’s say a superintendent who is mean to you during the day time, or someone known to be more strict than others, they tend to talk dirty after drinking, when I see

people like them, I would be like "wow". Sometimes I feel they did it on purpose but it seemed not to be the case.

1: It's like pretending to be drunk and acting ridiculously.

5: Some are like that for sure. He (points to 4) mentioned that how 2 people would hardly talk to each other during daytime, but once they start drinking, they would go differently like "Come on, let's forget about whatever that is between us and let's do something together!".

Interviewer: You think there was a huge difference.

5: For some people but not all. It's always that certain group of people who would act like that if you always go with the same people to a gathering. What do you think? (points to 3).

5: Would you think differently about him? Wondering why he became like this or thinking that he is so different when he drinks.

3: Getting drunk. I don't often get to see what he is like at work.

5: We're talking about now.

3: There's nothing like that here. I don't have any unforgettable experience from here.

Interviewer: Would you please tell us about what he is like usually and when he is drunk?

3: A regular elder person, sometimes childish, sometimes like one of your friendly uncles and not so serious. If he is drunk, he acts more naturally and claims to be capable of doing all sorts of things, things like showing his karate skills and all that which makes it obvious that he is drunk.

Interviewer: 4, would you view someone differently if you have seen them drinking at a gathering? Would they appear different to you?

4: It depends on whether he has done anything weird.

Interviewer: Have you ever met anything like it?

4: I can't remember. As I recall, most people who drink too much would also become quite talkative. They were unable to speak clearly, keep repeating or say things about their past for trying to build a closer relationship with you. It is pretty boring to be honest and I hate seeing things like that. If he really makes me feel he has completely turned into a different person it might indeed change the way I used to be respectful to him. I might change views about him whom I used to admire. However, I guess there ain't many people like this out there. I wouldn't have much opportunity to drink with a person like this. The chance to see people binge drinking at those occasions is rare. Binge drinking is more likely to happen at society functions where you are more likely to see some superintendent or supervisor whom you would never meet from a certain hospital.

Interviewer: Would you think differently about them after you see them drinking at a gathering?

4: Not so much. I didn't look up to him that much. I wouldn't know because I hadn't been around with him. It's common to see all sorts of drinking behavior around and I wouldn't be surprised by it. Behavior like this can also be seen in our elders, friends and relatives so it is no big deal. As long as this behavior is not associated with their profession. You view about their professionalism might be affected if they happen to be your teacher at the division.

Interviewer: Would you arrange such drinking activities if once you have become a supervisor.

1: I will provide alcohol but won’t force anyone to drink. Some doctor had told us once that drinking is supposed to be something you do with joy.

5: Does this doctor drink a lot or a little?

1: He drinks a lot and he thinks drinking is something you do with joy, you never should force others or yourself to drink. The gathering would be fun when your supervisor has provided moderately some alcohol, all sorts of tasty alcohol.

4: Have you and other doctors ever held some activities where lots of tasty and various kinds of alcohol are provided, have you?

1: Doctors who particularly know much about red wine would teach us drinking etiquette, like the origins of grapes used for making different red wine is something I never quite understand; introduction to some winery, characteristics [of wine] and how it [some wine] tastes like the flavor of orange or something. You would have to personally experience it [the taste]. You might feel no difference when you drink. This is some gathering where you will never see one person forcing another to keep drinking.

5: It’s a wine-tasting event.

1: Yeah, something like that.

4: The main theme of the event is wine-tasting.

1: Well, his main purpose was to have a gathering.

5: What did you go as?

1: I was also a student at that time. There were many students there.

1: I met this doctor from a medical volunteer group and he would invite students on different rounds from all divisions.

5: How many bottles of wine would you have every gathering? Say one type [of wine] is considered as one bottle.

1: Quite a lot, [there was] almost 10 types of them.

5: Wow, would you not get drunk easily by that?

1: I only drank a little but some did drink a lot.

4: Were there people drunk?

1: Yes, there were.

4: Who were they?

1: They were students and sometimes there were elder seniors who were residents at that time.

5: It was ok when they were drunk?

1: They would rush to the toilet and hold onto the [rubbish]bin.

2: My roommates who drink till they drop would go back to their dormitory.

Interviewer: 1, would you arrange drinking activities if you have become a supervisor one day?

1: Sure, why not.

2: Same as me. There will still people who like to drink. It’s no harm to provide some alcohol to add excitement to the event. I think it’s not necessary for an elder to force their subordinates to drink. It’s pointless. It’s meaningless if it

is for seeing some people make a fool of themselves by doing so. Everyone can drink moderately as they want when good alcohol is provided. You can also provide some better wine for everyone to try if there is enough budget. There is no need to urge subordinates to drink till they get drunk.

3: Basically, I will provide alcohol but I won’t drink. Everyone else is free to drink if they wish.

5: One day when you are a CR, it’s awkward to be a CR. You are the supervisor for the lower ranks and subordinate for the upper ranks. Would you lead the juniors to make a toast or not? Would you tell them to drink more in front of a chief physician? Different CRs would do differently. You meant to tell us from which division a CR you are.

2: I would see what the previous CR did in that division.

5: What if what every CR did from each year was different?

4: I will see if this would have anything to do with getting a promotion.

2: I would still do it out of politeness but in a moderate way, perhaps only to drink one glass.

1: Some people drink juice instead.

2: Never push others.

1: Take a group of people with me to show our respect to the elders.

Interviewer: 1 would also never force others to drink. What about you? (points to 3).

3: I think it will never be alcohol in my cup. I will lead the subordinates to greet their teachers. I won’t care what’s in their cups because it will never be alcohol in mine so I will not particularly ask others to do anything.

Interviewer: Would the rest of you prepare alcohol if you are to hold this kind of activity?

4: I shall prepare some alcoholic drinks, some that I personally would drink. It would be a pity if money is already spent on buying drinks for everyone but I haven’t got any to drink. I will choose the alcohol I want to drink and I won’t force others. If you are holding an event you would prepare alcohol more or less.

5: It’s pretty much the same case for me. I prefer certain types of wine to drink and won’t force others to drink. When I was a CR, people who were from the same graduating year as me enjoyed drinking very much. I did lead the juniors to make a toast and ask them to drink more in response to a drinking toast from the chief physician. At work, I have always said that I am allergic to alcohol and haven’t cared much about what the seniors think since I was a resident.

Interviewer: Everyone will prepare alcohol while arranging a gathering when you are a supervisor.

Interviewer: What do you think the meaning behind this work-related drinking behavior is? What is your view on the drinking pattern?

3: I don’t know what the seniors think about alcohol representing a way to show respect. The more you drink the more respectful you are. I don’t know much about this culture and why alcohol has been chosen to provide this function.

2: It think it is like people will think you are perfectly fit if you are able to drink a lot without getting drunk. Something to be admired for some point of view.

One way to look at this is that I might seem better than you if you are drunk but not me when we drink the same amount. Also, it is about showing the respect to the elders. It might be a disrespect to drink only one glass in response to one glass. One glass to 5 or one to 10 are better ways of drinking a toast to your elders. It's like a way of showing whether you are pretty capable of anything.

Interviewer: 2, did you mean it was to outdrink?

2: Yeah, I think so, if it's to drink with your peers but drinking with the elders is more like showing a respect to them as I said earlier.

Interviewer: To show a respect to them?

1: Probably I shouldn't use the word "respect" which is not appropriate here. It was more like to flatter instead of respect. No matter how much you drink, it's not something you do from the bottom of your heart and I don't think it is to show the respect as you really meant it.

5: It's like flattering. You would have to do some flattering as being a resident.

2: I also think it is like flattering.

1: On the contrary, the superiors may think it is ok to force their subordinates to drink in order to show their authority. I guess it's fun for them to watch the drunk subordinates make a fool of themselves. This is to think from an opposite point of view.

Interviewer: What does it mean to you personally? Your personal point of view.

1: About drinking.

1: Alcohol creates a good atmosphere and makes you get closer to your colleagues when you drink with your good friends. It's like a tool. You may think of it as music. People will have a better mood with the music on. It is like a tool for socializing with your friends and colleagues.

Interviewer: 1, you think it[alcohol] adds the excitement when drinking with peers.

1: Yeah, the purpose of this drinking behavior is different from that mentioned earlier. It is not like the flattering kind of behavior of drinking one glass in response to 5 as a way to show respect.

Interviewer: Would everyone agree with what 1 said?

5: It is like the saying of "all sufferings have their reward". All the senior doctors had been through the same kind of ordeal before. Once they had become a teacher, they would expect the subordinates to do the same [drink a toast to them]. This request often comes from the highly experienced senior doctors who have the idea of "all sufferings have their reward" in mind. There are many reasons to take alcohol in this case. The fact of the senior doctor being his teacher is the biggest reason of all. You have to consider things like whether refusing a drink offer [from the teacher] tonight would cause me trouble the next day; do I want a promotion; doing as told by the teachers means I have a better chance of promotion than my colleagues. After all, a promotion doesn't come easy for everyone. It also means there is a likelihood of your teacher to generously share his knowledge of the field with you. Some people who happen to enjoy drinking see it as a great opportunity to drink while some who normally couldn't speak for themselves under pressure take

it as a stress reduction. The senior doctor might think that "I was being mistreated in the same way before and it is finally my turn [to be in the reverse position]." People of lower rank would take this drinking issue seriously because future promotion of their job position can be determined by a physician-in-chief. Unless an individual shows no concern or they think they have a great achievement done during the day, that can exempt them from drinking alcohol, nobody would dare to refuse to take alcohol. Frankly, I think the majority of people would take alcohol no matter what. (P2-5)

Interviewer: You meant to be pushed to drink by the elders.

5: Yes, something like that.

4: It depends on the occasion. For me, drinking is something you do to be happy and relaxed. When there is no pressure there is less physiological and mental inhibition and you will feel a great comfort. It is a natural thing to do if it is for socializing purpose. This is different to drinking in an occasion where there might be outdrinking and superiors drinking to subordinates. Everyone has a different purpose. You are more likely to follow the rest under the influence of being in the same atmosphere. You tend to follow those people who achieve their purpose by drinking. Let's just say there are so many reasons out there. Unless you don't really care about the consequences it is inevitable to drink regardless of whatever career you have in Taiwan. It's the culture of the society such as going to a wedding, in a company where you have to obey your supervisors and drink as they ask. It's tiring to drink as you always have to outdrink others. If you have performed well, your teachers will drink more than you. It's tiring to drink [at these occasions]. You drink more than the teachers who eat more gourmet food than you.

Interviewer: Do you think drinking at a gathering would affect medical practice? What does it do to usual medical practice?

1: [It will] only if it occurs during medical practice.

2: Most of the time, not taking into account of being drunk for the whole day, the duration which the alcohol effect would last is about 2 days, one day being the day when you get drunk while the other day being the next day when you have a hangover. Unless you have some medical practice to do, say if I drink on a Friday night and I am off on the weekends, I don't think drinking has any effect on medical practice. However I have to be on duty all the time and it's always the right time to drink. I think having a hangover or being drunk will definitely have an influence on medical practice.

3: There is no major influence if you only drink at a gathering and as long as you don't drink in excess at work.

Interviewer: 3, for you it [drinking] won't affect medical practice.

5: It depends on when you drink. Mostly you would drink on Friday nights as it is less likely to deal with anything that requires professional judgement on Saturdays. I think you do realise there will be a difference in the precision of your operation the next day when you think you have drunk over a certain limit the previous day. You know when to refuse to drink. Some people will say they can't drink because they will have this major operation the next day.

Have you heard about this? I have. I have heard people saying like “ I am having a big operation tomorrow so that’s all I will drink for this gathering.” I think it [drinking] has some influence more or less. People will make their own judgement about how much drinking they should take to avoid the influence.

4: There is no harm as long as you don’t drink during medical practice, provided that you have considered whether drinking would affect your judgement the next day, whether there is any hangover symptoms and whether your actions are affected. Your professionalism shouldn’t be affected. Another situation to consider is that to say I am a surgeon and I am still an exempt employee type of chief physician, which means I have to take responsibilities even if I drink when I am off work or without being on duty. Turning to others for help will affect professional image or patients’ rights and interests.

5: I have heard before about one year-end party at the division. A patient had bleeding after operation on the same evening when he was off duty. You could smell strong alcohol from him when he was called to the cas2

4: It does sound awkward. If I am ever to be in that situation whether I would show up or not totally depends on whether I am able to do so or not. I will turn to someone who must also be a chief physician for help. I will tell him I have been held up by something and need his help.

5: I think it’s better to turn to someone.

4: I would try to avoid showing up. I will look for a chief physician on duty and ask him to do the explanation.

5: What will the families think if they see this? You [the patient] need the operation to stop the bleeding. Would you [the drunk doctor] be able to do the operation for me, or who else?

4: If you decide to show up it’s better to get someone else to do the operation. You have to explain to your patient that you can’t do the operation because of drinking, as long as you have made it clear. You must be honest in such situation or else you should never show up.

5: I think it [alcohol] has some influence after hearing things like this.

Interviewer: Both of you think drinking at an event can influence medical practice.

4: It’s more likely to see things like this in surgery. The custom at the division of internal medicine makes it [incidence mentioned earlier] unlikely to happen. If anything happens while on duty the residents or chief residents will take care of it.

5: You don’t have to deal with it in person.

4: You feel like it’s nothing serious depending on the division and [patient’s] case. It’s no big deal if you drink after work as long as you won’t be affected the next day. It’s a must to see the chief physician in person if it’s a surgery case.

4: Sometimes it’s about dealing with some easier task at the surgery division and a chief physician is required.

5: [We] talked about complications after surgery.

4: It’s necessary to be careful.

Interviewer: You think whether medical practice will be influenced depending on the division?

4: It’s not supposed to have an influence except for accidents. Accidents do happen and this is when you have to take the divisional factor into account.

Interviewer: So there will be an influence [of drinking] on medical practice.

4: Anyone with a common sense would agree to that. There are doctors who drink everyday, a special group with binge drinking habits. If they drink only for social purposes they won’t have their work affected [by alcohol].

5: They have dinner gathering on Friday nights.

4: Sometimes there happen to be occasions where you must show up in person. It will be a trouble for doctors who have taken alcohol under this circumstance.

5: Unexpected.

4: You must come up with a backup solution for unexpected things like this. Don’t do anything stupid. You should say that you can’t show up because you are caught up with something, this is some excuse that the families might accept. On the other hand, showing up drunk may influence the trust of families or patients on you.

Interviewer: Lastly, does anyone have any feedback or more to share? Feel free to add anything you might miss out.

1: Just remember something, what others had said about going to work the next day while drunk is the same as the extreme tiredness you get from working on duty without sleeping for the whole day.

Interviewer: Any more unforgettable experience from the rest of you? Has it [drinking event] affected the medical system?

4: Considering you and your patient might be affected, you have to pick the right time if you want to drink a lot. You might choose to drink on Saturdays and have the hangover for the whole day on Sundays. Drinking too much has some influence more or less.

Interviewer: 4, what is your personal view on drinking at a gathering?

4: It’s ok when everyone is free to drink happily and there is a good atmosphere. You could refuse to go to occasions where you will be forced to drink. For me, I probably won’t attend the surgery gathering at the division. It’s no fun to go there and some might not be pleased with you.

Interviewer: Anything to add? We will end our interview here.

Group 3

Physicians (n=2) & Residents (n=4)

Group 3

Interviewer: Please introduce yourself including your job position, things like your interests and favorite food.
1: R1. I like marathon and light food such as congee with dishes.
2: A chief physician. I like bread.
3: R1 in neurosurgery. I like to travel and my favorite food is crab.
4: A chief physician in neurosurgery. I love steak.
Interviewer: [Your] interests are….?
4: Driving.
5: I am a resident. I like cycling, movies, singing. My favorite food is meat and I like meat.
6: Residency. I play balls and travel. I love spicy hot pot.
Interviewer: Do you often drink alcohol with your meals? At what occasions and how often?
5: I usually drink at wedding banquets and this is when I get to drink mostly. How often I drink depends on how many wedding banquets I have that month.
Interviewer: How often?
5: About 2-3 times a month. It depends on how many banquets there are in a month. I drink mostly at wedding banquets but hardly any at other times.
Interviewer: It's drinking about 2-3 times a month. What about others?
4: At year-end banquets where there are lots of doctors who like to urge others to drink. These happen about once every 6 months or a year.
Interviewer: Those year-end banquets eh?
4: Any banquets of all sorts of medical societies, about once every 6 months or a year and you would eat and drink there.
4: There is more times to drink without eating, [drinking] without food is once a month.
4: Drinking with meals is once every 6 months. Drinking without food is about once a month.
Interviewer: You drink about once a month on average.
3: I drink at home in winter, red wine about once every 1-2 months. I usually drink at gatherings about once a month.
Interviewer: Once a month. At what occasions?
3: Dinner gatherings with friends.
2: Mostly drinking when eating with friends at dinner gatherings and this is about 2-3 times a month. Sometimes I don't drink for up to 6 months. It depends.
Interviewer: It's also at the dinner gatherings with friends and once a month.
2: It's random. Sometimes it's 2-3 times a month or I don't even drink for 2-3 months.

Interviewer: Do you have divisional gatherings, year-end parties or spring receptions? Would you describe any drinking experience from those events?
5: A battle-like situation?
Interviewer: What was the drinking experience you had there?
4: It was an exciting event for everyone. People would drink a toast to each other. We would drink to show our respect to teachers by making a toast to them.
2: To show respect to the teachers.
4: Was your question about the amount or just drinking [in general]?

Interviewer: Sharing the experience of divisional gatherings, spring receptions or year-end parties, what were they like?

4: A divisional gathering with the purpose to express appreciation to staff. It was a happy and bustling dinner gathering. Red, white wine, beer and Shaoxing wine were provided at the banquet. Everyone including students and teachers, subordinates and supervisors made a toast to each other. Everyone was pretty happy and excited. No one was forced. I haven’t met anything like where you were forced to make a toast at a dinner gathering, taking 10 glasses of alcohol in response to one glass from others. It’s more likely to happen at year-end parties where there will be teachers, colleagues and seniors.

Interviewer: What about the rest of you?

3: I have only been to year-end parties so far. In that evening, we had to make a toast, to the teachers and we were led to meet everyone else. We were asked to finish our drinks.

Interviewer: There were only doctors there?

3: There was probably only me.

4: It was a meeting for all the young residents. There was a banquet after that meeting. There were a group of young residents as well as a group of senior teachers at that banquet. It was for sure the young residents had to accompany the teachers to make a toast as a way to show respect.

Interviewer: 3, it was also the teachers you were drinking with?

3: Yes.

1: I drink only at high school reunions or weddings. Once every 6 months for high school reunions. Once every 2-3 months for weddings. I don’t usually drink. The most recent welcome party for residents was when I had drunk about 2-3 months ago.

Interviewer: The people you were drinking with were colleagues.

1: Teachers and colleagues.

6: I had attended a spring reception held by the obstetrics and gynecology division. It was similar to what others had described, possibly being urged to drink or something but the atmosphere was great and everyone was getting along well.

Interviewer: Any work-related drinking experience and at what occasion? Was it after work with colleagues or during internship? Please briefly describe what it was like.

6: I drink with my colleagues. It was after one tiring course. Everyone gathered to have a drink or so. It doesn’t matter where we would go but we would always grab a drink somewhere after a dinner gathering.

Interviewer: With colleagues or seniors?

6: No seniors, normally there are only colleagues.

Interviewer: Where do you go?

6: It depends on what you want to eat. You can have beer at a restaurant that serves fried dishes. We would drink at someone’s place if we are having some western style cuisine. At a friend’s place and something like that.

Interviewer: Would you describe the atmosphere and what did it feel like at that time?

6: It’s surely a great atmosphere at an occasion like that. Close friends would be able to drink casually and get excited easily.

Interviewer: Others with any work or internship related drinking experience, please share with us what the situation is like.

4: Seeing a patient who got hit by the head after drink-driving. Does it count?

Interviewer: Not during your work but something having to do with your colleagues.

2: A year-end party that is.

1: You meant a private gathering.

4: At work and after work. Personal drinking behavior at private has got nothing to do with [things you do] after work? The people, things, time and places which have anything to do with work-related drinking experience.

4: It’s unlikely to drink with seniors or juniors at places after work.

5: It’s mostly about being with colleagues at year-end parties, spring receptions or weddings. Such as a celebration event at the end of PGY year, things like getting together to drink. For most people, you wouldn’t go to work but [attending those events] after hospital work.

Interviewer: Does everyone have experience from attending year-end parties or spring receptions? Would you see others differently if you see them drinking at an event?

4: Before, after or during the time they drink?

Interviewer: Anything.

2: It was usually a relaxing event where you would get to see a different side of them. People are more relaxed after drinking and tend to talk more freely. It’s nothing like reporting the situation of your patients to your teachers and different from the situation at work. It depends on the event you go to.

4: I think it [drinking] might have affected the way I see them more or less. Like 2 had said, after drinking at social occasions people definitely act differently from what they normally look like at work. They would already seem different while they are making a toast to each other.

Interviewer: You think there is an influence.

4: Yes, more or less.

1: I haven’t got much experience of going to year-end parties. It more or less changes the way I see this doctor if they are usually serious but become completely different after drinking. I don’t have any experience like this to shar at the moment. This doesn’t happen between my colleagues and me. I don’t have much experience drinking with teachers so I wouldn’t know. I don’t get drunk while drinking with colleagues. I would just have few to drink and go home if there is work the next day.

Interviewer: What do the rest of you think? Would you change the way you see this person normally?

3: Like 1 said, I don’t drink that often with teachers and only drink occasionally with friends. Possibly I would [change the way I see them] if drinking with teachers.

Interviewer: You have such experience with the teachers? That is you would change the way you see them?

3: Yes, definitely.
5: I also think it will have an effect on the way you think about your friends or colleagues. You will have slightly different views about them. It is probably because people are always very hard-working during work. Drinking makes it easy to see someone being relaxed and sometimes acting in a way different from what you knew at the first time you met them. This is likely to happen.
Interviewer: There is an influence more or less.
5: Yeah, I think so. Being drunk might make you throw up seriously, pass out, or speak in an offensive way and things like these make you look pretty different. We only drank a little at weddings. I haven't met anything like so at private dinner gatherings with best friends. Only at big celebration events, the annual celebration events where I get to see a drunk situation like that.
6: Similar to what was just said. I think I won't change the way I see them. [To see them] drink or work are two different things. You wouldn't think differently too much about them if you get to see more of what they are like after work.
Interviewer: You think it won't have an effect on you.
6: No, because they are showing a side of them being relaxed.

Interviewer: Would you arrange a drinking event if you are personally to host a dinner gathering?
3: It depends on the type and purpose of the dinner gathering.
2: If the gathering is to make both the host and guests happy everyone is free to drink whatever they want and no one is forced to take alcohol when they don't want it.
Interviewer: You would prepare alcohol at those events.
4: Yes, I would for those events, anything from soft to alcoholic drinks.
1: I would do the same. Teachers might get thirsty and they might want to drink alcohol. But we might just have regular drinks among peers.
Interviewer: Do you mean you won't prepare drinks if it's a peer gathering?
1: We are afraid that teachers might get thirsty.
6: You would know whether it would be a drinking event during the time you are organizing the gathering. It depends on the people who are going [to the event]. It's more casual with the peers, only buying drinks when needed. You would prepare drinks first if there are teachers.

Interviewer: Do you think drinking at a gathering, this type of behavior will affect medical practice? What is your personal view on this?
6: It's ok if you have got some sleep [afterwards]. It's ok if you have got some sleep after a gathering before the next day you will be on duty.
1: There will be an influence if you drink too much. For me, I don't drink that much but it will have an influence on me if I do.
3: It's ok as long as you don't drink too much until a hangover which might affect your work the next day.
Interviewer: You meant there is no influence on medical practice, or is there?

2: It depends on how much you drink. There is an influence if you drink too much otherwise there isn't.

3: It depends on when you drink, things like drinking before the day when you have or don't have to be on duty, or drinking several days before you have to work.

5: For looking at others, it does depend on how much they drink. There is sure an influence if you drink too much to work the next day. You may even fall asleep and have no energy to work the next day. You should drink up to the amount that would still allow you to have the energy to work. I think there is no influence if you still feel ok when you wake up afterwards. For me, if I have to work the next day I will definitely make sure not to drink too much. I think if I drink too much it is likely I will be affected at work the next day. I would try to avoid things like this to happen though there isn't any so far.

Interviewer: Have you seen things like this then or..?

5: No, I haven't but I have seen many drunk patients. It's highly likely to see those who get too drunk to work the next day, or even those who are urged to drink get so seriously drunk that they have to stay in the hospital. I haven't personally met anything like this but it may happen as a result from drinking too much. It hasn't happened to myself or my friends. The problem lies with the amount of alcohol taken.

Interviewer: So you think there will be an influence.

5: Drinking too much will affect your work the next day. You drink to the point of ending up in a hospital and being unable to work, this is a 100% influence, being unable to work that is.

Interviewer: What do you think [of] the idea behind this work-related drinking behavior? Your personal views about drinking behavior and what does it mean to you?

5: Everyone has some fun and gets relaxed. It means, to have some alcohol to drink at a gathering of course. It's like to help a group of people relax to chat and make them to meet each other easily at a wedding gathering. It is to build a closer relationship and the same thing applies to a private gathering among friends. What was the question mostly about again?

Interviewer: What does work-related drinking behavior mean to you. What are your views on this behavior?

4: At our society, if we are to have meetings related to work, you will have this drinking behavior based on social etiquette for reasons of subordinates showing respect and manners to their teachers or superiors at some occasions. Some people might think it's not polite without making a toast to your teachers. Would this have an effect on your job? There might be. I think there is. The customs of this society lead to this particular etiquette. Whether it would have an effect on the job itself, it's a rule of social behavior which you have to comply and your job promotion, other evaluations or evaluations of your superiors about you would be affected if you don't comply. This is how drinking behavior may have an effect on your job.

Interviewer: Social behavior is mostly your personal view [on drinking at a gathering].

2: A social behavior or to enhance a closer relationship to say it in a better way. Formally speaking, it’s also a type of etiquette. The bad side of this depends on how your teachers treat you. This type of behavior has to be caused by those like your superiors instead of your subordinates. It might therefore be weird to ask us this kind of question. It’s not about whether we are willing to or not. There is definitely an influence. We could only comply and drink as told by our supervisors.

Interviewer: 1, what is your view on this? Your personal point of view.

1: Like 2 had said, I think it’s a personal preference. Some like to drink juice while some like to drink alcohol. You might happen to meet a teacher who doesn’t like to drink then you don’t have to drink. A personal preference. You have to comply out of politeness at occasions described earlier. Drinking itself is considered as a personal preference, something like drinking juice.

Interviewer: Personal drinking behavior is based on politeness.

6: At some occasions where there might be people unknown to you, you can get talkative to know them more after drinking. You need something like alcohol to help you get to know these people. Everything else is similar to what was just said.

3: In terms of the job aspect as said earlier, it’s a form of socializing activity.

Interviewer: Would you continue to have this drinking custom once you have become a supervisor?

3: If I am a supervisor people are free to drink whatever they want. They don’t have to if they don’t want to. There is nothing like making a toast in particular. I personally think I wouldn’t be particularly happy if someone drinks a toast to me. Anything is fine as long as I am happy when everyone else is.

Interviewer: Do you think drinking will not particularly make you feel relaxed or happy?

3: It depends on the occasion. It’s a social behavior out of politeness when drinking with teachers. You wouldn’t feel too happy if you have to drink a lot at that kind of occasion.

Interviewer: Would you change or keep this drinking custom once you have become a supervisor?

2: If I am a supervisor to hold an event I would make sure both the host and guests are happy. You can drink if you want to and it’s fine if you don’t. I won’t feel particularly happy if someone does drink a toast to me with alcohol. If both you and I don’t feel like drinking then we won’t drink.

Interviewer: What about the rest of you?

5: I think [it’s] pretty much the same. If I am the host or supervisor from the unit it doesn’t matter to me whether they would drink a toast with alcohol or juice. It’s fine when everyone is happy as long as everyone is happy and enjoys the gathering. Whether I will feel particularly excited or not has got nothing to do with if there is alcohol or not. Everyone is casual and won’t be affected by the fact that I am the supervisor. Every guest can drink to have fun and they can also be happy without alcohol when they don’t feel like drinking. There will not be anything like to show respect with alcohol. There will be nothing like that when I get to be the supervisor.

4: Same here.

Interviewer: Any unforgettable dinner gathering?

5: Misbehavior after drinking or when the event is over?

Interviewer: Any parts to say about work-related drinking behavior?

2: It’s like those said earlier.

Interviewer: Anything you would like to add?

4: I would like to know about the main subject evaluated in a questionnaire like this. Those were open questions being asked. It was like to tell a story. They were not relatively more systemic questions. To be honest, I couldn’t find the real theme from the questions being asked. The questionnaire was clear though. It asks things like whether you get drunk when you drink, yes or no; do you drink while on duty, yes or no. It was hard for me to make an answer specifically to your question. A lot of the things I replied might not be relevant to what you want in your study, the real objective of your study. But this [what was discussed earlier] has got nothing to do with doctor-patient relationship. Drinking and work itself, sharing experience of drinking with your friends and colleagues or others, unforgettable [experience], all of these have nothing to do with doctor-patient relationship. If there is an objective [but] what we have answered didn’t meet the objective of your study, this is how I feel. Those were open questions. We are interviewee helping out the interviewer who could carry out the study. What we have answered seems unrestrained and random, did the reply we made seem helpful to you?

Interviewer: We did mention about medical practice.

5: There was one question on whether work would be affected the next day and I think this is relevant. I think drinking at weddings or year-end parties is not particularly related to doctor-patient relationship. What I shared from my personal experience seems to be irrelevant.

5: There is one on sharing the experience of any misbehavior or a wedding gathering where everyone comes and goes happily having some alcohol to drink. What sort of the story you would like to hear? It seems anything. I have no idea if drinking has anything to do with doctor-patient relationship. Sometimes I really didn’t know how to reply.

4: The questionnaire, hoping the patients… Is it true… guessing you… about your questionnaire… explaining to patients.

1: I would like to share something during PGY before when I was in the internal medicine of a community. There is this cardiologist whom I didn’t see in person has bipolar disorder. He drinks quite often. From what a nurse specialist had said, cases of AMI are common in the division of cardiology, bipolar disorder [causes the doctor] to drink constantly. This doctor would drink while working on the case of a patient having a hematoma. This doctor takes a bottle of wine with him/her and leaves it next to him/her. This is the only case I have heard so far.

Interviewer: You heard this from a friend?

4: “During my rounds at a certain division, from a community hospital there was this cardiologist having bipolar disorder and drinking all the time.” This is where I see the [issue of] medical practice is involved.

4: A teacher from our division giving an account of emergency situations….There was once an experienced senior physician whose patient required an urgent surgery. This physician, who was thought to have taken alcohol possibly from a wedding gathering, showed up with overloaded alcohol breath but inevitably tried explaining the medical conditions of his patient in an incoherent manner. The ability of making judgment of drunk physicians with strong alcohol odor is questioned by patients. We helped to make further clarifications to the patient's family after the physician attempted making his explanations. This is a detrimental influence on the doctor-patient relationship. Physicians taking alcohol would definitely cause patients or other doctors to have doubts in your professionalism. Not to mention that you have to do a brain surgery for my[their] father. This is someone whom I personally witnessed to be in a tipsy state with alcohol odor. (P3-4)

Interviewer: Did the patient refuse to have the operation?

4: No, he would die if he didn't have it. Although his family agreed to the operation they still had doubts about him and felt he was not trustworthy. The operation was not performed by that doctor for sure. He had to explain to the family because that was his patient. The recovery went well afterwards. I don't know if the family had questioned about the whole situation afterwards but it's for sure they had raised doubts right at the moment of the emergency.

Interviewer: You believe it has an influence on the medical system.

4: Definitely, drinking at work regardless of being drunk or not will absolutely have an influence on your medical practice as well as the doctor-patient relationship.

Interviewer: Other people have heard or had any similar experience?

3: From the hospital I used to work for before, it's said that surgeons love to drink at gatherings, and I had heard about one senior chief physician who went to the wrong patient while he was making ward rounds after coming back from drinking at a gathering.

1: Even without drinking I could still visit the wrong ward while doing ward rounds

2: There is sure an influence. Sometimes I drink right before the next day when I will have work to do. I will feel sleepy and dizzy when I drink too much, and even a hangover can affect [me]. For me, I haven't had the experience of drinking during work but the experience of being on duty with a disturbing headache from a hangover. In that case, I was affected but nothing serious happened. This situation doesn't happen usually but occasionally. It's like the feeling of discomfort you get from being on duty while sick. You have to stay on duty despite the sleepiness and discomfort. It's like overworking, the same feeling you get from having performed the operations for the whole day and there is still more to do the next day.

Interviewer: You have had such experience?

6: It's impossible to go to work straightaway after drinking. This kind of situation only happens occasionally. Like 2 had said, occasionally you might drink too much and have a hangover the next day. You will be like it's fine

as long as everything goes well. In this case, it’s not likely to deal with everything to reach a 100% pefection but probably up to 60% or something like that.

Interviewer: Anything to add or missing?

1: If I am the supervisor I might….. It’s unlikely you will be hosting the event alone once you have become the supervisor. If I don’t feel like drinking on the day of the event I will inform the chief resident. The chief resident can help me remind the residents to avoid making a toast with alcohol to their supervisor. If one of the residents doesn’t want to drink I will also inform the chief resident who will also inform other residents. You won’t be the only supervisor [present at that event], some people who take it for granted will still go to you with the alcohol they prepare, even if you don’t want to drink!

Group 4

Physicians (n=6)

Group 4

Interviewer: Please introduce yourself and tell us the type of food you like.
6: I have been in this medical field for nearly 30 years since I started working in the hospital in 1987. I am more disciplined and working normally without having too much of an eventful life. I like light meals or Japanese cuisine.
5: I have been working in the hospital for about 19 years. I spend most of my time in the hospital and sleep mostly at home. My life is boring and I work a lot. I seldom drink. I haven’t had any to drink for six months. I used to be picky about the type of food I have. Now I am ok eating whatever available in the hospital and as long as I can fill up.
4: I don’t mind any type of food as long as there is something to eat. I am said to be a person whom is easy to feed.
Interviewer: Any type of food you like in particular? Like American-, Western- or Japanese- style.
4: I think I am not picky about the type of food. I am only picky about the people I am eating with or the eating atmosphere. I am fine with the food as long as it’s not something I hate so much. When you ask about the food I like I will say it depends on the people you are eating with and the environment.
3: I don’t have any food I like in particular, everything is fine as long as it’s not something I hate. There is not much of the food that I dislike. I can eat pretty much all kinds of food, it doesn’t matter if it’s Chinese, Western or Japanese cuisine. I can accept most varieties of dishes in any certain cuisine.
2: Similar to what others said. I am ok eating with Asian food, Chinese, Japanese, Thai or Vietnamese cuisines. It’s also easy to feed me. Just eat simply. I don’t like eating with people who will make the eating atmosphere bad. It’s the best to eat with your best mates. Same as 5, I haven’t had any alcohol for years, and it was not only 3-4 years. I really haven’t had any alcohol for ages. I don’t drink with meals and this is pretty much all from me.

Interviewer: From what everyone had said, it seems all of you are not picky about the type of food you eat. Do you often drink alcohol with your meals? Or regardless of having a meal or not.
2: A habit of drinking or not?
Interviewer: Yes, such as eating and having alcohol to go with your food.
6: You meant like ordering alcohol first when going out to eat. I think most of us don’t do this.
Interviewer: At what occasion did this happen before? How often?
5: We ordered red wine or white wine first before ordering a meal of the Western cuisine, for Japanese cuisine we would order sake to drink first before ordering the meal.
Interviewer: At a restaurant? How often?
5: Yes, once a week. We would drink with meals. There is still 80% of total amount of whiskey left in the bottle which was opened 2 years ago.
6: Did you use to drink with meals of Chinese or Western cuisine?
5: I didn’t drink when I had meals of Chinese cuisine but I drank white wine with meals of Western cuisine. For Japanese cuisine, I would drink sake with it. This was when I ate with my family or at some high-class restaurant. I

don't drink that much now because I have to drive. That opened bottle of whiskey left there was opened for my dad.

Interviewer: What about the rest of you?

6: I seldom drink, but when I do my face turns red very easily. I have a slower alcohol metabolism. To be honest, I don't drink on my own initiative unless there is a right atmosphere where there are your close friends or long-time classmates. I actually think we don't drink at all now but we had more alcohol when we were still students. I drank more when I was a student because I didn't stay at home but lived on my own. We would have alcohol at a gathering with friends. It was more likely to have alcohol during my school time. Working in the hospital is more regular and always busier so we won't have too much extra time.

Interviewer: Did the rest of you drink?

3: I would after work when I was a resident. Residents who know each other well would go out to eat and drink alcohol.

6: He can hold his liquor well. (laughs)

Interviewer: Also at a restaurant? How often?

3: Yes, we drank when we ate or we would drink while chatting and buy some dishes to eat with alcohol at home. It wasn't often but once a month to the most. As a doctor it's rare for us to drink entirely for alcohol itself but more like for eating with your best friends.

4: If there is a group of best friends or wine-tasting event I will drink whiskey.

Interviewer: At what occasions?

4: I drink at home. My son would drink with me because I am under a lot of pressure. Ha, it took me a year to finish that bottle. I would open many bottles (laughs) and drink once a week.

Interviewer: Would you mind sharing work-related drinking experience such as divisional gatherings, spring receptions or year-end parties.

2: After a conference, there is usually an event like the year-end party, spring receptions as mentioned earlier or welcome party.

3: A welcome party.

Interviewer: Please describe what it was like, the atmosphere at that time?

2: Everyone was friendly (laughs) and had a glass [of alcohol] in front of them. They would save at least one glass of alcohol ready for making a toast later when the supervisor came.

Interviewer: A divisional gathering?

2: What was said sounds like a divisional gathering where everyone was also nice. You could even hardly finish one glass of red wine if you drank it sip by sip. It was only a regular drink and was considered to be a regular drink.

6: You should think of it as a different drinking habit. We don't force others to drink in the environment like we have at our division. You can drink whatever as you feel like it. This is rather different at some other hospitals where there is a different drinking habit. I think the original purpose [of such events] was made for good, it was hoped that everyone who participates could relax and be relaxed as much as possible. We usually have a lot of stress every day.

Everyone should try to relax as much as they could. It's best for a senior to tell everyone to get relaxed and everyone would do so. About how to get relaxation, this is when the young people would be asked to start drinking to get loosen up. Once you are loosen up and stress is relieved sometimes it's hard to control your emotions. This is when you might start to force others to drink. I think this kind of situation is quite common so far in the medical profession. It's ok as long as there is no drink-driving otherwise you will get caught and end up in jail. It's now better as compared to before when the situation was a lot worse. Someone who is not able to hold their liquor well it is inevitable to see them throwing up and being unwell. I totally get used to see things like this.

4: The atmosphere is the key, the atmosphere at that time.

6: Like what 4 said, [You would feel] the atmosphere is great when you drink out of your own will, which is different from that when you are being forced to drink.

4: I drank the most when I was serving in the military.

6: Do you think you were forced to drink during that time?

4: I was serving in the Taiwan Garrison Command and able to hold my liquor quite well at that time.

6: Do you think you were forced to drink or you actually enjoyed it?

4: Drinking in a way like everyone else does.

6: I guess you did enjoy while doing it in that case. Sometimes the situation is quite bad and it's pretty annoying when others forced you to drink.

Interviewer: Could you tell us more about what occasion that was?

4: The most common drinking event in the troops is when someone has got a promotion or completed the military service. They would have this habit of drinking Kinmen Gaoliang (laughs).

Interviewer: Do others of you have the same experience? Any of you might have been [to such event]?

2: I have met something like "1 against 3". The teacher drinks one glass [of alcohol] whereas you have to drink 3 glasses.

4: That is what we call a situation of you being forced.

3: It wasn't really like the teacher trying to force you [to drink], it was more like because of the custom or tradition.

2: It was the early 90's [when alcohol drinking was] taken to its extreme. During the time of GS system, others around you would urge you when the teacher finished their drink. They would say something like, "How could you only drink one glass as well when the teacher took one glass and finished his/her drink." It was a bit like forcing [others]. They didn't ask you to actually drink up but you just felt like you had to do so. It was only ok when you had finished three drinks. It wouldn't be ok if you had one but someone else before you had 3. It would be unbearable if you kept drinking in the way they demanded.

5: This is more likely to happen among residents. I once had a dinner gathering with the people from the neurology division, the residents were there first and XO [cognac] was provided. Nobody was drinking but it was required to make a toast, something like "1 against 3" (drink 3 glasses in response to one). I

ended up finishing half bottle of wine in that evening and sleeping in the car afterwards. Everyone was gone when I woke up. How come [I] didn’t show up on the second day. Another unpleasant experience, it was during R5 or R6 when I had a Mongolian BBQ with Dr. A in Tianmu, the situation was like an endless drinking event and I had 7 glasses of beer. I had to drive on the highway afterwards and felt it was ok to drive. I nearly hit the median strip when I was reaching Wugu. At that time Linkou was still quite remote and I parked my car on the side of the road. I slept [in the car] until dark. I remember it was lunch I had but I woke up at 10 pm wondering why I ended up there.

2: We were seriously responsible for drinking up at the welcome party we had at our division.

5: The residents would drink a lot and the teacher would show you more respect before you become a chief physician.

4: Basically, it depends on the situation where there would be someone making the first move. It all starts when someone begins to take the very first drink. The inhibition is removed.

6: Exactly!

2: It’s hard to sit down again once you have stood up.

Interviewer: Whom were you drinking with? Who were they when you were a chief physician?

5: Generally, the residents would feel pressure to drink with teachers or chief physicians. You would drink and it’s hard to stop under that pressure. As a chief physician, you still have got to drink when you have to. You would drink some when attending some meetings. You would know when you are about to go over the limit and stop drinking when you know you won’t be able to drive afterwards if you don’t. It’s ok to drink a toast to the teachers. You know your limit and would stop before you get too hyper. I take it as a normal social interaction. The way they used to drink before, I think it was a bit….

3: They must drink till they drop.

Interviewer: Could you give us more examples of the past experience, were they the teachers who urged you to drink when you were a chief physician?

5: It was when I was a resident.

Interviewer: How did the teachers make you drink?

5: In that case, you felt like you had to drink because you were from the surgery division. You showed up to drink with everyone. I often drank till I drop when drinking with the CR during my time at the bigger division of general surgery. I wanted to drink a toast to show my gratitude to seniors and experienced specialists who taught me things about operations. This is how endless drinking started. It was a dangerous thing to do when I think about it now. It was ok when I was in the dormitory but I had experience of hitting the median strip for several times when I was outside.

Interviewer: Had the rest of you been urged to drink?

3: I wouldn’t call it as being urged to drink. It’s more like a culture. Like 4 said, a Japanese culture, you have to make a toast if eating with elders and teachers and when there is alcohol provided on the table. As I recall, it is usually those bystanders who happen to urge you to drink 3 glasses of alcohol instead of

one glass as originally demanded by the teacher. My teacher wouldn't make this type of demand of drinking three glasses instead of one. Those bystanders, colleagues and other teachers are those held responsible for making this kind of demand. There is always more than one teacher present on this occasion! (P4-3)

2: Occasionally I have to be ready and drink as "1 against 3" when drinking with a teacher who can hold his/her liquor well. Although I personally think there is no harm intended, it's also likely to meet someone who wouldn't stop asking you to drink even if you said you can't. They even think you are pretending if you are lying there throwing up. This is really something unacceptable. It is thought necessary and normal for people to relax by drinking some alcohol with meals. Eating, drinking alcohol and having relaxation are three things bound together. It seems the goal for your day is not achieved when you only drink alcohol and have meals. It's thought you won't have fun unless you have met the conditions of having your meals and getting drunk to be relaxed. In this case, you will be required to drink more and this is when urging others to drink starts. It's meant to get loosen up on those occasions.

Interviewer: You usually drink with your teachers and colleagues. Will you change the way you see them if you have seen them drinking at gatherings?

3: What way?

Interviewer: The way you see them normally and when they drink.

3: I don't drink with people whom I don't know well.

3: It's ok because I drink with best friends.

1: What was asked before this?

Interviewer: Whether there is a habit of drinking, on what occasions and how often.

6: Talking about different drinking culture, some like to urge or even force others to drink while some whom you know quite well drink at will.

2: If someone does get too drunk from last time we would make them go easy on drinking the next time. If there is anything like an accident of one car bumping into 3 cars, we possibly will not prepare alcohol for the next time.

3: Is it to change the way we see them from the aspect of their professional behavior or their personality?

6: It is the strong effect of alcohol. Alcohol changes a person. Some people become completely different.

Interviewer: It's what you think about them at work or at a gathering.

1: It depends on your purpose of drinking and the occasions, it makes a difference. It's nothing special if you are to drink to enjoy yourself while drinking with a group of people. It will be different if you are to show your relative position in the group you are drinking with. Say there is a young doctor unknown to most people in the group trying to show himself [by drinking] so that the people will get to know who he is and he will gain the group acceptance. It's ok for ordinary gatherings which are different from those at places such as hospitals. There is different hierarchy present in the workplace of medical field, where you have the supervisors, seniors, young and retired personnel, new comers. The recognition [from this workplace] you

think is different from that thought by your supervisors. Your supervisors who like to drink want to add excitement to the event, everyone would try to urge others to drink. As we discussed earlier, how much you will drink depends on the people who participate. It is a must to add excitement [to the event] if people from the division [who join the event] are calm and quiet. What is it about the idea of adding excitement and atmosphere of the division? If the custom of the division is outdrinking, everyone would do so to be in the same atmosphere and show you are part of the team of that division. Drinking is fun but whom you would drink with? It depends on the hierarchy or drinking custom. It's more complicated for [drinking in] groups. The custom for our surgery division here is based on the idea of mentor-to-student. The hierarchy difference between a mentor and student is obvious. As a resident like you is not a friend but student [to someone in a higher position than you]. It is different. A resident will always obey orders from superiors and what they say is generally not taken into consideration. (laughs). This is the hierarchy difference which is usually not so evident but only on occasions like those events.

Interviewer: Any unforgettable experience as we talked about urging to drink just now?

1: One thing is that a person is asked to drink or eat by their supervisor, he would do so to show he is capable of doing things like this. It's a way of showing off by doing something more than others have done. When everyone drinks a toast to others some will drink up while some will drink as "1 against many". Superiors showing their authority or a regulation to someone in this group are reasons for doing this. Another thing is when a young person who intends to show this aspect of his ability. He doesn't necessarily drink or hold his liquor well but he would do this as a way of showing his respect and without being forced by anyone else. He would put a pressure on himself. This is different. He does this under the pressure he put on himself. Is there any pressure on the person who doesn't drink? There is also pressure on them (laughs). When they don't drink and refuse to drink even if told by their supervisor the pressure would be there as they go back to work afterwards. [What they do gives their supervisor the impression that] they will not listen to whatever the supervisor has asked them to do. This is a different issue, not something we talked about as social drinking but another kind of social drinking.

Interviewer: Would you arrange a drinking event if you are personally to host a dinner gathering?

4: It depends on whether there is enough fund(laughs). Drinking beer costs less and beer is [a] more ordinary [drink]. It costs more for preparing wine of higher quality and this may make you spend more on wine than on food. One has to consider the budget first and then the size of the gathering. If it's for an annual event for which there is usually more budget, drinking always brings relaxation [for people]at such event. Alcohol is often a good way to add excitement to an event though it's not entirely true all the time. Sometimes you need to come up with other ways to add excitement, though nothing beats the effect of alcohol. Alcohol brings physiological effects, after all. Everyone

is all the same. Once you drink the inhibition is reduced and you will get hyper. It’s hard to make all the people responsive to an activity such as singing or a magic show but the effect of alcohol is general. Alcohol is usually a better way to add excitement when there is enough budget.

1: If it is you who are to hold such an event?

5: It’s [alcohol] a must. (laughs)

6: You don’t act as rationally as 4 does. (laughs out loud). My intention is to look for and invite people whom I think would be nice and friendly. Alcohol is a good thing depending on how you use it and provides an alternative option. For me, it’s also a must [to provide alcohol at an event]. I will prepare good alcohol to share with nice people.

2: It depends on the type of people [whom you drink with]. You will chat more with those who you haven’t seen for 3-5 years and care less for anything to make everyone hyper. Everyone could drink one or two sips and it’s also fine if they don’t. When there is a different gathering where there will be family people may drink a little more to add excitement. If it’s a gathering for teachers and students you are likely to drink till you drop for showing your respect to the teachers. On a different occasion like this you will definitely hold a bottle in one hand and a cup in the other [for making a toast to your teachers]. There is a difference in intentions [of drinking] depending on the people whom you drink with. There is no doubt that alcohol is a very good catalyst. Different intentions are there for drinking with different people. The way to look at alcohol in different levels. There is of course [different varieties] beer and XO [whiskey] as well as different alcohol content.

Interviewer: It looks like everyone would prepare alcohol when holding your own event.

1: It depends on the participants. I would only drink beer if 4 invites me to eat out. (everyone laughs).

1: The problem is not to prepare alcohol or not. One thing is that I remember when I was younger organizing an event at another hospital where the teachers and seniors were invited. It went well and I got promoted. Once a supervisor invited me to eat out for lunch and it surprised me that it was noon but he/she was ready to drink alcohol. This was not ok for me when taking medical practice into consideration. At the end, I wasn’t invited because he/she was not pleased when I intended to reject him/her. The superintendent attended the lunch gathering instead but it turned out that I didn’t get the promotion in the same year when this happened. (Others burst into laughter) It’s cruel but this is what the culture was like at that time. The supervisor was even in a higher position than that of superintendent. He could make the decisions [on your promotions], what you gonna do? You can do nothing about it. You may also choose to drink [to join the drinking event], there is still pressure [on you], right? You will have to watch how much you drink. You may be called to the hospital if you drink while eating at a dinner gathering and being on duty on the same evening. Things like this do happen and it will be a trouble, isn’t it, a trouble. You do get to see this kind of stuff happening and this also makes you to think twice before making a decision on preparing the alcohol., Once when I was a resident, I was invited to a

dinner gathering near Ximen Ding in Taipei despite the fact that there was a shortage of doctors at that time. A call about an urgent operation came from the ER in the middle of dinner. The traffic condition 20 years ago wasn’t as good as now and I drove back to the hospital in a rush, the lucky thing was that I didn’t drink before driving. Think about it, going from driving in a rush to handling the urgent operation. For eating out, first you have to choose the right companions, second is to have the right time and thirdly it depends on your work. How can you possibly go out to eat when you are on duty? There was a shortage of doctors at that time and I believe it was the same for other hospitals. Would you prepare alcohol to go with meals? At home, would you have alcohol with your meals? I don’t believe you would open a bottle of red wine or beer at night, I don’t think so. But why would you do this when you are inviting guests to your gathering? It’s known by most people that the westerners drink red wine at nights. In Taiwan, drinking with meals at night is considered as something what a drunk would do. There are only few cases where people do drink with meals, right? Whether to prepare alcohol or not depends on the place you will eat. There is usually no alcohol provided when eating street food while there is beer when eating at some better restaurant. You would prepare alcohol depending on the place and atmosphere and there is no certain rule. Say if 5 is preparing a gathering, it’s not necessarily he has to provide red wine, brandy or whiskey. People will not focus on what to go with their meals. For everyone, alcohol is not a regular thing to have. Drinking alcohol is about a drinking atmosphere created in the group. We talked about the drinking behavior, alcohol helps you get hyper and lowers your inhibition, you dare to say things you wouldn’t normally say. However, I don’t think it’s right to get drunk and bad-mouth others as you could in that atmosphere. It’s also likely to see someone getting drunk by beer and bad-mouthing others if you drink stronger alcohol with meals. Alcohol content, your mood and what you want to express are all closely related. Back to our subject, whether to prepare alcohol or not depends on the occasion. We [Taiwanese] don’t drink regularly. I am not sure about how westerners drink. There is not many people who would drink now. You may have beer for dinner but hardly any alcohol for lunch.

Interviewer: You talked about medical practice. Would drinking at a gathering affect your medical practice?

6: It does only when you binge drink till alcohol poisoning. There used to be more drinking at gatherings while on duty as compared to now. I think this is a subject worth discussing. In the medical field, the relationship between supervisors and students, like the way as we guide the young doctors, is really considered the same as the relationship of mentor and apprentice. It is something different from a direct management relationship between someone and their supervisors. The relationship we have here is closer. There is complicated issue involved when it comes to wine-tasting of doctors or things like that. It’s true for sure. Like the way we drink a toast to someone as “1 against 2”, [the relationship between us] is not the one between a supervisor

and a subordinate, sometimes it’s like the one between peers or more like the one between a mentor and an apprentice. on how much you drink. There is an influence if you drink too much otherwise there isn’t.

1: If [we think of it as] it’s between you and your kids, you won’t say something like “ You drink one glass while I drink two” to your son. You wouldn’t say so to your son, would you? But this happens when drinking with residents. How’s your relationship with your son?

6: Well, the teacher definitely doesn’t say things like that, but the students do.

1: Will you accept if your son says that to you?

6: Definitely not. (both laughs)

1: Somehow, you have to abuse the resident, don’t you? When abused, don’t you expect them to tell you they are fine instead of the truth? But if this happens to your child, would you stop them [drinking] when they come to you? You would definitely stop them, wouldn’t you? It’s pretty obvious. The relationship you have with your students, the relationship with you. What relationship this is, it is all about authority for sure. This is hierarchy. You will not need to show the authority of a father to your kid under that circumstance. Have you ever seen a father attend a drinking event at the kindergarten or elementary his kid goes to, have you?

6: Not for me but probably for a drunk.

1: From my division, there are doctors who do that. I totally don’t get it. By law, it says clearly kids of certain age are not allowed to drink. How ridiculous it is for that doctor to insist on a kid from elementary or kindergarten drinking beer. This is some senior doctor I have seen in our division. I think it is an exception for that particular drinking behavior and what they think. How to ask or stop, how do you ask or stop your kid….

6: It sounds like a custom to me. This is something what the teachers used to do anyway and you would just do the same. You won’t do nothing but abuse the students, everyone follows and no one would mention anything about making a change.

1: Why do you have to abuse?

5: As discussed earlier, it’s ok for me to be in that atmosphere. Once I become a senior chief physician I won’t force my subordinates to drink. People can drink a toast at will. Drinking is a fun thing to do. You drink more when you can and it’s ok to only raise your glass [without drinking] when you can’t drink. What happened in the past sounds like an abuse to me. As I recall after all these years, like 1 had said, you were scared of going to those events where unpleasant things such as extensive drinking and throwing up might happen. I would try to come up with excuses to avoid going to those events.

1: Would you stop a foreigner who drinks red, white wine and generally drinks moderately when they drink a toast to you as a resident?

5: I will drink up and they are free to do whatever.

1: [There is] Pressure! You drink up whereas the resident can do whatever at will, in fact, this actually means the resident also has to drink up.

6: This is the culture, something special we have.

Interviewer: We talked about whether the medical practice would be affected, also things about professional image or professionalism.

4: Speaking of medical practice [affected by alcohol], there are two things concerning this issue. One thing is that whether or not [drinking] would cause shaking of the surgeon's hands or problems in making accurate judgment. The second is that whether [drinking]it leads to problems of interacting with patients. Would you allow a physician, who explains your medical condition with a noticeable alcohol flush on the face and strong alcohol breath, to perform your surgery? In a case where a surgery fails as a result from such medical malpractice, it is surely closely related to the [doctor-patient] interaction. (P4-1) This interaction was different and things like this were rare in the past when the patients didn't have too much choice or the [their] relationship [with doctors] wasn't so intense back then. Now it's different. We are not allowed to drink while on duty or get back to hospital to work after drinking. Patients can tell [if you have been drinking or not]. Medical practice is related to the interaction between both sides. I believe there is nothing like that happening nowadays. In the past, it was often that the [people from the] whole division went out thinking they would return to the hospital when called and no one was left in the hospital. This was likely to happen before but not now. Now it's considered to be an unacceptable behavior as it's likely to cause problems easily with patients. The influence on the medical practice as you talked about, I think firstly it is related to whether this part has an influence on the person who does this [drink while on duty]. Secondly, it depends on whether if there is an influence on the interaction with patients, or a restriction on doctors doing this. It will make the headlines if a patient finds out a doctor who has been out drinking while on duty.

1: About drinking and doctor-patient relationship as we mentioned. Who hasn't received the wine as a gift from the patient at an outpatient department? I think everyone here has. Why? Giving [you] wine as a gift is a way of the patient showing their gratitude regardless of whether you drink or not or how much you drink. Would the patient expect [you] to drink? Of course they won't. It's only an option for them [patients] to express their gratitude by giving you wine. Speaking of hand tremor in professionalism, sometimes alcohol prevents hands from shaking. (Everyone laughs out loud). This [hand tremor] is not how we look at this situation. It depends more on how the patients sees you. You feel nothing when you only drink a little but your patient may not accept you in this way. For several times, I wasn't on duty but still returned to the hospital when something was up or I needed to provide my opinions [for some cases], though this is something that wouldn't affect much. Would it be an effect [on medical practice] whether I could make it to the hospital? It surely would if I could barely walk. It's fine for everyone who knows how to control themselves. Talking about the part on professional image, the condition you are in seen by your patient, or the one with your colleagues and subordinates are all about [your] professional image. You will be humiliated [by the patient] right away if you get seriously wasted. It's not acceptable for a young doctor like you getting drunk and having an operation

to perform the next day. This is something you have to be realistic about, isn't it?

Unlike being a surgeon, there will be problems for you if you have drunk the previous day. Doing well on the previous day is what a surgeon like me does, and this is a compliment, isn't it? (Everyone laughs out loud). It is a professional image to your peers. What, in fact, your peers have in mind? Are they wondering about how you would walk home if you have got yourself completely wasted? On the surface, they might call you a legend, right? Is there anyone who drinks like this? Yes, there is. Anyone who drinks to end up in the ICU? Yes, there is. Instead of being forced to drink, this is someone who drinks on his own initiative. For a surgeon, alcohol poisoning is transient. Those, who refuse to drink concerning about their professional image, will get rejected and influenced by others unless they have good excuses. It is worse when your supervisor loves to drink. You and everyone can be more casual if no one is into alcohol that much. It's a trouble when you have a supervisor who would drink whisky even at lunch time, what you gonna do about it? It will be a tough time for you having a supervisor like that. From a patient's point of view on professional image, the patient wouldn't expect a doctor to get so drunk like that. It's ok to drink moderately. You have to try hard to maintain a good professional image among your peers. It's different.

Interviewer: What do you think the meaning behind work-related drinking behavior is? What is your personal view about this? This behavior of drinking is related to work.

6: To show yourself.

1: The difference for me is that there is nothing like this I have seen at various overseas occasions I have been to. We have more behavior of ourdrinking, drinking till you drop or throw up and things like these are considered to be our tradition or culture. Another thing is pressure, I think these are the reasons for work-related drinking behavior.

Interviewer: What do you think personally?

2: I think [alcohol] was used as a tool. A tool used to define an equal or a relative relationship. Showing respect to the teachers. I think I expect my subordinates to show me respect and I will use alcohol as a tool to test. Whether this person is open-minded, casual or fussy. A person might get very concerned about performing an operation the next day and refuses to take few drinks as told. What sounds like an innocent excuse may seem something like he takes a dislike to his supervisor. It [drinking] has become a custom and a tool. It's hard to say if there is no misuse.

6: When we talked about drinking red wine, I think it is something a relative normal drinking behavior. For one thing, you can definitely learn something new from it, about wine-tasting. It's for social, making friends and socializing, rather than a tool. You can learn something as well as make friends with no intention of doing anything in particular.

Interviewer: Make friends.

5: There are three ways of looking at this. First, it's related to the authority within the relationship between a superior and subordinate. Second, it's a tool for making friends. Sometimes we had the chance of drinking with the doctors or patient's family from other hospitals or in other countries. This happens in Asian society. Once after a meeting I had a dinner gathering with doctors from Hong Kong and China, it was amazing to see how they drink. The doctors from China took off their shirts leaving only undershirts on them halfway through dinner while drinking Gaoliang. Getting nearly naked eating grilled meat while drinking red wine. A relationship with the patient's family I had seen in Xiamen shows me how strange their culture is. The patient's family would invite you to have a meal and drink every time on the day before you would perform a major operation for the patient. There is no such custom in Taiwan. I insisted on rejecting them. Every local doctor we asked there is used to this. That is something I am not ok with. I would definitely get a good rest [before the day I will perform a surgery]. Drinking too much will make your hands shake or in a bad condition. They [the patient's family] seemed to accept the excuse I had come up with. They probably thought I rejected their invitation because I didn't take the operation seriously.

Interviewer: This is a culture of Xiamen?

5: Not sure whether it's that of China or Xiamen.

2: It's closely related to the difference in ethnic groups. Unlike Europeans and Americans, Japanese will drink to an extent that a group of Japanese would stand in a row singing happily the military song while taking their pants off to take a piss on the side of the road. They have a strong sense of subjectivity and tend to show more solidarity focusing on interactions among group members like those found in ants. In Western countries where individualization is reinforced, subordinates are free to express their thoughts. Medical profession in Taiwan is greatly influenced by that of Japan especially during the time of 70's or 80's. However recent changes are found in the drinking culture of our medical profession under the influence of Western individualization over the years. It is not guaranteed that the subordinates will take alcohol as told but instead one may be asking for embarrassment by pressuring them to drink. You wouldn't dare to refuse to drink when you were told to do so in the past time. At that time, the situation where the chief residents would stand up and take a lead to start drinking is completely different from now. It is impossible to deny the fact that drinking culture in medical profession is closely related to the difference in ethnic groups. Times are changing and a wide impact on many aspects could be anticipated. China hadn't held a great international status over past hundred years but this is all going to change. They have their own culture and we were used to be strongly affected by Japanese culture. (P4-2)

Interviewer: This is talking about the influence of culture.

2: The difference between East and West contributes to a big difference in influence

1: No, it's not as simple as that. Speaking of drinking culture, have you ever heard about or seen anything in the legal or industrial professions where one

person drinks a toast of 3 glasses against 1 or 9 glasses against 3, as it is commonly seen in our medical profession. This type of phenomenon can be repeatedly seen in the medical as well as the legal profession. The drinking tradition in our legal profession used to be similar to that of Japan. However, it [the drinking situation in the medical profession] is usually worse than that in the legal profession. The medical profession itself has been deeply affected by the drinking culture. (P4-1) There is a lot of things involved and responsibilities to take in medical profession. Teaching a young physician means I am responsible for him/her. When it comes to them taking care of patients, I will treat them in a way stricter than the way I treat my kids. If you want to do this part in this way. A tool, as we talked about earlier, there are many aspects to it. First, drinking ability is one thing. Second is that when we judge how a person drinks, you may talk about it in a different way and from a different angle. The way how it[tool] is used depends on how the superior does it. If this superior has bad opinions about you, he uses it [tool] as an excuse to say you are not good enough when you can or cannot drink. It's not a good thing to do when judging you directly at work. Everyone faces the same problem, yeah, so what is actually the purpose of drinking? Ok, if you look back, it's a way, an agreement reached among the peers. A way that everyone accepts and has agreed on. I am not the only one but also everyone else who knows about it[this way] when I have to do things like this.

Interviewer: It[alcohol] is a medium.

1: Everyone tries to outdrink others when drinking together. If it's an invitation from 6 and 6 shows up with the supervisor instead of a majority of other supervisors, the one with the highest authority has the right to do this. The lower the rank you are the less the power you have [to do it]. We have to drink even with the new residents. This is indeed a tool. It's not a problem you can simply solve if you only look at it from one aspect.

Interviewer: Those of higher rank have more authority.

1: No, it's more like those of higher rank are entitled to use this tool, but not for those of lower rank. Anyone in the supervisor position or above have the right to do so. It's ok for the bride and groom if you drink till you drop at a wedding you attend (laughs) It's ok for having this behavior at your table or at 2-3 other tables. But it is impossible under those conditions [drinking at a hospital gathering]. It's a group behavior, isn't it. There is an effect like the check and balance [of powers]. It makes you seem eccentric when I do it but you don't. This is different from [attending] a wedding banquet.

Interviewer: Is there anything to add or missing?

Group 5

Physicians (n=5)

Group 5

Interviewer: Please introduce yourself, any interests or type of food you like?

1: I love to eat. My interests include making friends, and lots of others. [I am from the] emergency department. I can’t really eat too much fried [food] which I enjoy a lot. I have been gaining weight these days and this worries me. I like [any food] with sauce or being spicy.

2: I am an attending physician in surgery and love to cook and eat. Any rice-related food is my favorite as long as it’s not meshed up like congee. How to cook it[rice-related food] well is also one of my hobbies.

3: I like sweet ice cream quite a lot but I am on a diet now. I enjoy indoor activities such as listening to the music or reading some books.

4: I am an attending physician in nephrology. I love teppanyaki because I like it when the dish is hot and you get to see the process of others cooking for you. My hobby is to play table tennis.

5: Hi, everyone. I am a nephrologist. I eat all varieties of food. I am glad to join this wine-tasting like group. Mostly I cook for others at home. I have been doing this on my own initiative since I was a child and interested in helping out my parent. I prepare most of the dishes [at home]. I seldom eat out but cook at home very often. Everyone is welcome to come and cook with me so we can learn from each other. 2 is a great cook but he often prepares complicated dishes. I tend to prepare simple dishes which don’t require a lot of cooking [procedure].

Interviewer: Do you often drink alcohol with your meals? Do you drink at all or any habit of drinking?

1: I drink wine when having steak in a restaurant. We drink white or red wine when eating out with the assistant superintendent.

Interviewer: At a restaurant.

1: Not even once for red or white wine [in a week]. [Once every]3-5 days for beer.

Interviewer: Where was this? At a restaurant or home?

2: I remember when I was young during the Chinese New Year Holiday or three major holidays of the year, there was Japanese red wine at home. It was like a ritual. We don’t normally drink at home but we do get wines as gifts from patients. Although our storage room is full of wine and we don’t know what to do about it, I never open any bottle to drink. There are many reasons, for one, my wife doesn’t like people who drink and I don’t have a good liver function for the second reason. My sister-in-law is a doctor in the US and she loves beer. My wife buys beer occasionally for her when she comes to Taiwan. It depends when eating out. We add alcohol [to dishes] while cooking.

3: For me, I drink alcohol very often and it’s got something to do with my family. [The relation between] Drinking and family. You are less likely to drink if you are from a family where there is not much drinking going on. My mom and uncle from my family love to drink alcohol, rice wine, a regular wine. I had more chances of trying wine when I was a kid. My uncle used to drink wine of high alcohol content. Whisky or brandy had been a trend for a while at that time. I drink a variety of alcohol, beer or wine of high alcohol content.

Interviewer: On what occasions?

3: I don't like to drink in public. I sometimes drink beer on the hot days. I find it weird that how many patients love to give doctors wine as gifts. I seldom open [those bottles of wine as gifts to drink] but I do drink occasionally or drink on the cold days. I was into beer a while ago but now I am into whiskey as well as red wine.

Interviewer: How often? [You] do drink a variety of alcohol, many kinds.

3: Once a week but [I drink only] a little every time, not much. I drink to make myself sleep easily and drinking helps my sleep, but I can't drink too much. I drink only a little because I have poor liver metabolism [of alcohol]. In public, I have to drive. It's hard to control drinking in public.

2: I have heard some doctor friends who have insomnia will drink to help them sleep better. Everyone like us being a doctor knows to drink moderately. Drinking a little helps sleep better but it doesn't work with beer. Beer helps cool down. At other times, we drink during dinner gatherings where we would open bottles of wine and there are always people who suggest having a drink together.

Interviewer: Does the rest of you also drink?

4: My family and I don't drink. It's inevitable to drink at social occasions for doctors. I would try to avoid drinking because I think it looks bad as my face turns red whenever I drink. Once every 1-2 months [is how often I drink.]

2: I would like to ask 4. I felt the same after hearing what 3 said. Sometimes [drinking] is related to your elderly or the custom form your family. Like my mom and relatives, they never drink but my father and relatives from his side drink a lot. I am closer to my mom but I lived closer to the relatives from my father's side and got more chance of trying alcohol.

4: It depends on different periods of time. In the early days, my mom loved to drink a lot when I was quite young and I wouldn't have the chance to experience [drinking]. They don't drink right when I am all grown up now.

5: I don't drink that often, probably drink once or twice a year. I only drink when asked by the elderly at important social occasions. I have a different background growing up. 30 years ago during junior or senior high school, we had a lot of alcohol given from others at home. There was a lot [of alcohol] and even a room specially for storage [of these alcohol], such as commonly seen whisky, about 30 bottles of black-labeled Johnnie Walker, red wine of 1-20 bottles, cognac, brandy of special product of origins including all brands and 4 different designations: REMY, 3STAR, VSOP and XO. There were lots of them and even more than those sold in the liquor stores. We didn't have any problem with alcohol supply. I drank a lot at that time too and even though I was underage my dad would ask me to drink anyway. We had alcohol of all brands, everything, you name it.

2: You don't drink now but why did you drink at that time?

5: I don't drink now because…. Why is there a big change… It has been on news recently about processed red meat, red meat [considered] 2A [carcinogen] regardless of how it is cooked. In fact, alcoholic beverages are considered class 1 carcinogen. For many years, I have stopped drinking that much for

health reasons and this was my primary reason. We have a small room filled with alcohol which is mostly sent away as gifts. It is really magnificent to see the amount of alcohol we have despite the regular brands. I immediately know the level of wine once I take a sip of it!

Interviewer: You do know a lot about wine.

5: There was a fixed tax [on alcohol sold by shops] of monopoly bureau. You knew the price right away once you saw the box. The price for a bottle of XO was from $6,100 to $6,300 and very expensive. The one [XO] of crystal bottle cost about 10 grand and more.

2: Do you have one bottle opened currently at your home?

1: I don’t often drink strong alcohol on my own.

5: I am scared of being told to drink when visiting the elderly. There might be whisky worth 20 grand or something. I find this kind of alcohol troubling. I don’t enjoy drinking Shaoxing.

Interviewer: Everyone has an experience of drinking. Could you share work-related drinking experience, what is it like?

3: It depends on the division. People drink till they drop at welcome parties. But everyone is getting older these years.

5: Once at a year-end party, Dr. X was so drunk and nearly peed in public!

3: I used to drink a lot when I was at the thoracic society of Veterans General Hospital but not so much at National Taiwan University Hospital.

1: Different [drinking]custom exists in different hospitals. When I was in the emergency department at Veterans General Hospital, about once a month, our supervisor or seniors would hold a birthday event where people would gather to drink. You raise the glass with ease and drink down the alcohol without frowning!

3: The division of surgery at Chang Gung Memorial Hospital has been quite conservative these years.

2: Chang Gung where there isn’t much drinking is the odd one out. I know [they drink at]the division of orthopedics, we invited the CR from the division of orthopedics to drink at the dinner gathering with us this week. There is a dinner gathering once every month. There is a rule of drinking 3 glasses of alcohol for being late [for the gathering]. CR is always the first one to drink few glasses and this has become a tradition at our workplace. I think there is a certain meaning to it[drinking] in the culture of Taiwan. Drinking helps build a closer relationship. It is in the same sense of feeling to be in the same group. There was no or hardly any alcohol on the table [at the gathering] for our cardiac surgery division, but only tea or drinks for kids were provided. We still made ourselves in charge of drinking alcohol because we were like the odd ones out. Doctors from cardiac surgery division hardly drink at work. For me, I rarely drink at work except for drinking with pharmaceutical sales.

1: As being a doctor at the emergency department, I would have some rough idea of how many would be coming in today if it’s the day for year-end parties. Giving them intravenous drips to provide them a plenty of water to help them urinate is something that happens all the time at year-end parties.

Interviewer: Please describe in details about what exactly happened there and anything unforgettable?

1: It was a very exciting event. You can drink to every reason, reason of being late, being popular or loving your chief physician. You drink to show your respect to others. Anything basically, it’s a hyper moment, you drink to all kinds of reasons!

Interviewer: Who were you drinking with?

1: Anyone, it could be your supervisor or colleagues.

2: We have nurses and assistants from the operating rooms. Staff from the outpatient department also drink. They don’t drink strong alcohol too much, but mostly red wine or beer. It’s strange as most doctors or medical staff know it’s no good to drink alcohol, especially in excess but they… I think the reason they drink a toast is not because the alcohol is a good thing. Don’t over drink as you know it’s easy to get uncomfortable if you do so. It’s ok to drink moderately. It’s a little harmful. To drink for us.

1: The person you are drinking with is possibly your best mate. We would never do harm to each other. We would go easy on drinking. Sometimes there is a special exception, I would want to drink a toast to 4 if drinking with 4.

2: To gain a recognition from each other through a ritual or something.

Interviewer: May others share your year-end parties, spring receptions or divisional gatherings?

4: Year-end parties and spring receptions [I had] are similar to what 2 had said. Drinking a toast to others and having a closer friendship is what you have in mind. Recently, our supervisor had invited us to a small group of gathering which one table would fit. What I feel is that, at that time, it was like drinking to drown your sorrows, reduce the stress and comfort yourself. Everyone could forget about anything unpleasant or the process of doing all the hard work, this is what I feel.

Interviewer: It was the teachers whom you drank with?

4: Yeah!

2: Sometimes it’s mostly the residents, PGY or their friends instead of the teachers, people of lower ranks.

Interviewer: Any unforgettable experience?

5: I think this kind of culture has been changing slowly. You might feel kind of being forced at that time with your teachers. This custom hasn’t been observed that often these years. Young doctors don’t really follow this kind of tradition. In about 10 or 20 years, I guess, it’s as less likely to see this custom as seeing smoking behavior now. One unforgettable experience is when I was newly promoted to be an attending physician. One attending physician went to drink a toast to the superiors and chief physicians, he went to a table where all the superiors and supervisors sat. He meant to make a toast to the chief physician only but other superiors [at that table] gave him a hard time. He who was in a deep trouble tried to drink a toast to flatter the chief physician. He felt weak and was about to collapse after drinking 8-9 glasses of alcohol. Surely there were people who told him earlier not to start making

a toast without taking your limit for drinking into consideration. Every single person from that table asked him to finish making a toast to them.

2: There is always someone keen on urging others into drinking on almost every occasion. He/She who possibly holds their liquor well drinks on their own or invites others to drink with them. For me, if the chief physician is not present and I am the only superior there, I would sit there and have my food without thinking about going around to make a toast to nobody. It[making a toast] is only to show respect and I never really want to make a toast.

Interviewer: Did you say the superiors made you drink? The drinking gathering mentioned earlier.

2: Some would walk around to make a toast everywhere and some would encourage others to drink. You wouldn't dare to urge your elderly into drinking. The elderly drink on their own initiative. You can only urge your subordinates or colleagues into drinking.

1: Making a toast is only a form. You make a toast with tea. Making a toast is not purely for drinking anymore, it's only a form. Making a toast and drinking alcohol are separate now. Unlike in the past, you had to make someone drink till they drop or try to outdrink to show off. This custom has been changed otherwise there would be more problems of drink-driving. It doesn't matter how well you can hold your liquor you will still get caught [in the alcohol test]. You might only get drunk from drinking 10 glasses but one glass is enough to make you get a positive alcohol test result. Unlike that of drinking to show off in the past, the [drinking]culture has changed these years.

Interviewer: A change in culture.

2: I will drive [to an event] if I don't feel like drinking.

Interviewer: Have you been urged into drinking?

2: Yes, surely I have.

1: Sometimes there is no urging needed. There is pressure. A pressure of all the people having their eyes on you. It's hard to turn down when a senior would say to a junior "You are ok with 3 glasses, ain't you?" You only have to drink to be at ease. Under the circumstances, there is pressure from colleagues.

Interviewer: [being told]by your seniors?

1: Seniors or colleagues. It will be my turn and hard to refuse after they take the drink. Pressure from peers. If 4 has drunk 5 glasses I feel like I must do so too. Others' expectations of us at that time were a pressure to us.

Interviewer: Any others with a similar experience?

2: There were lots of people who would urge others [into drinking] when I was young. The colleagues did it and the teachers did the same too. There are not many who can do that to me as I am in a higher rank position now.

Interviewer: Did you urge your colleagues or juniors into drinking?

2: Occasionally, if there were people who started drinking.

4: Sometimes [drinking] it is to add excitement, if [the event] is dull and awkward, and I am in the role of being a senior inviting the juniors.

2: I joined the wedding of my colleague who worked at the same division as me. If you are closer to [this particular person], you can use the drink provided by

the host to start a conversation. Something like, “come, let’s drink”. You will also try to do something to make the juniors talk.

1: It’s not entirely negative about it [drinking]. Sometimes alcohol provides a great opportunity or creates an atmosphere. It is sometimes also for showing your gratitude for others. Having one glass to drink is also a concrete way of showing your respect to doctors.

2: Alcohol is like a medium, something slightly more expensive. It doesn’t necessarily mean it’s good for your health. With [the effect of] different amount [of alcohol taken], it’s amazing. If you ask your junior to drink, looking at the positive side, it is his opportunity to show himself. There are many aspects here to discuss. It is therefore not entirely wrong to make someone drink. There are too many meanings associated with this situation.

Interviewer: Does the rest of you have the experience of making a toast or being urged into drinking with your juniors?

2: I usually drank it down regardless of being urged into drinking or urging others into drinking. At first you don’t see many people drinking but it all starts when 3-4 people out of a table for 10 begin to drink. It gets awkward if people don’t really drink. When people start drinking to some point others start urging them into drinking gradually.

1: It came to my mind that pouring somebody a drink is also considered urging them into drinking. I think in some way pouring the drink immediately to the empty cup is also a behavior of urging others into drinking.

Interviewer: Any unforgettable experience or experience of interactions with teachers and colleagues?

5: Many people have similar experience. Say if there is a wedding of our classmate, we would mix and make a special flavor type of drink containing not only wine but also 2-3 other stuff. The [groom] has to finish the drink. I think he [groom] plays the role of entertaining others and has the task of entertaining others. [He has to] drink up the “cocktail” containing black vinegar, soy sauce and 3 different types of wine mixed together.

2: There is definitely an effect of entertaining. We have to perform at the welcome party at our surgery division. We have to drink 2 glasses of red wine once we get onto the stage and 2 more glasses if we can’t answer the questions. This is a game in which you are kind being forced into drinking. It is basically an entertainment related to the rank of the job position. The amount you drink depends on how many years of working in the hospital. For us, the rank still exists in doctors, nurses, interns and medical staff. What sort of things you are allowed to do in the certain year and there is no “grade skipping,” which is only allowed for attending physicians. For others, you have to go by the rules year by year. It’s certainly more obvious at a drinking event of dinner gathering.

1: It’s 3 against 1 (drinking 3 glasses in response to 1 glass) for drinking with chief physicians and 2 against 1 with seniors. Something like this does exist and you can see it from the drinking culture.

2: Rank plays a major part. A difference of 5 years [of working in the hospital] means a difference of 5 glasses [to drink] between you and me. This is common at XX hospital and nothing like this has been seen at Chang Gung.

1: There is also a difference in drinking in terms of gender difference. They will go easy on the girls.

2: Girls who drink get the same compliment as the guys do.

Interviewer: Would you change the way you see the person normally if you see them drinking at a social gathering? If you are to drink with your teachers or colleagues, what do you think about these people you drink with?

1: I don’t have any example to talk about. If I imagine it and say one junior comes to make a toast to me and drinks 5 glasses, I think he is being very respectful to me (laughs). I don’t have any actual experience. I imagine if someone has done that to me I would personally have a good impression of them and find them easy going.

2: For this matter, whether you feel like drinking or not.. whatever he does… Someone keeps making a toast to me when I don’t feel like drinking. This is something different from what I feel when I drink on my own. It’s better to drink moderately. I have seen a new R1 who likes to drink wildly and he makes a toast everywhere with a glass in his hand. I don’t like anyone like this and this kid’s behavior can be corrected. There is definitely a change in the way we see them. Unless I don’t care, once I see whatever you do, I think I will change my view about you more or less.

3: There is sure an influence [on our view about the person who drinks]. Some drinking behavior, some will get relaxed after drinking. The behavior without a disguise changes back to be the original behavior of that person. Sometimes from drinking…personality and attitude, is there an influence? I think there is. Some [people] seem to be more innocent and pure after drinking. It’s better to drink moderately. You can tell if a person has some discipline or not from seeing them drinking. If what he does goes out of my expectation I will form a negative impression of him.

Interviewer: There is an influence, both good and bad.

5: I personally don’t have such an experience. For reading from the literature, everyone goes to a year-end party which is meant for “forgetting about the age” and a tradition that Japanese follows. There is an obvious difference [in people] with different ranks. Less experienced staff are not allowed to drink a toast to experienced seniors. The only exception is a year-end party where you are allowed to make a toast. After the year-end party, the seniors would know if the new staff is being respectful or aggressive depending on the [drinking]interactions between each other. For me, I don’t drink much and have much experience. This is all I know about how it is like to be at a year-end party.

Interviewer: What about [your view about]the people you spend most time with [at work]?

5: There is an influence more or less. It’s related to the culture. A senior can invite you to drink but you can’t do so to a senior. The only exception is when there is a year-end party, forgetting about your age. This is a tradition where

people gather to celebrate the end of the year and it’s assumed that you are allowed to drink in whatever way you like.

Interviewer: You will be affected and slightly change the way you think about this person whom you spend most time with.

1: See if this person has done anything unacceptable, if he doesn’t, the odds are that I might still have a good impression of them.

2: You know what a person is like if you get to spend time long enough with them at the same division. For a bigger group, it’s hard to find out what a new comer is like when drinking at private or whether they even drink or not. It’s not about the change [of the behavior]. It [a drinking event] can be a good opportunity for people or a supervisor to know what this person is like for the first time. It’s a good way of making friends among peers and getting to know your friends better. It seems there are quite different views about the behavior of drinking between East and West. We have a similar view to what they have in Japan. I am not sure about their view in China, but it seems like a similar situation.

Interviewer: Under the influence of Japanese culture. Does each of you hold your own gathering activities and will you prepare alcohol for some good events like this?

4: We have some wine tasting[events]. There is a group of people with whom we would have gourmet food and drink good wine together. No one will feel the competitiveness when everyone is there for having some wine tasting experience. This is like what we do at the division of internal medicine, we [go to the event] purely for having an experience in wine tasting. We learn about wine, [whether it has a] metallic or mineral taste, this seems to be something that all wine learners have in common and it is different from socializing.

Interviewer: Will the rest of you prepare alcohol for the events you hold?

1: It depends on the participants and intention. Knowing that it’s not easy to get drunk from drinking beer, I will choose a restaurant that sells beer if eating with interns, so that we can avoid anything bad happening if we choose to drink strong alcohol instead. Depending on the people [we drink with] and whether alcohol is needed… There are all adults at a mentor gathering and the teacher has to take some responsibility. Whether [you] drive or not…

Interviewer: It depends on the participants. Do others have a different point of view?

3: I think you are freer to do whatever you like at a dinner gathering nowadays. Frankly speaking, there are fewer and fewer people who would drink to get so wasted. It’s mostly about wine tasting instead of specifically drinking alcohol in particular. Like in the Western countries, red wine and white wine are popular, mainly red wine has become part of a meal. Don’t force others to drink, take me as an example, I don’t necessarily prepare alcohol when holding a mentor gathering, and [people] are allowed to drink juice.

Interviewer: You won’t necessarily prepare alcohol.

3: Yeah, it depends

5: I have an experience similar to that of 3. I don’t drink on occasions where there are my family and friends. If drinking at a restaurant of Xinyi Place where there is beer provided I would remind them to drink moderately. It’s necessary to watch out the amount of drinking, unless it’s a wine tasting where you have an intention to drink when you have to taste the alcohol. For other event, you won’t specifically ask for alcohol to drink.

Interviewer: You won’t necessarily prepare alcohol but other drinks instead.

5: It[alcohol] is only one of the options.

2: The gatherings which I personally arrange are usually held at lunch time within the hospital. I didn’t prepare alcohol once for a gathering held at my place. We will order alcohol but not strong alcohol if we know the friend whom we eat with drinks alcohol. We will never prepare alcohol for our closer friends, we know like few of those from our division who never drink alcohol. We will never prepare alcohol no matter how close we are to them. It’s necessary to prepare alcohol for things like year-end parties as an exciting atmosphere is considered a must in an event like this. It depends on how you drink. Some teachers find it weird for a dinner gathering without alcohol. It’s something like a celebration to eat at a work-related dinner gathering.

Interviewer: For adding the excitement to a dinner gathering!

Interviewer: Will drinking at a dinner gathering affect medical practice?

2: Everyone here has studied medicine and knows how alcohol can affect behavior. You know you can only drink after work. It’s impossible to drink at lunch time or before making rounds in the evening being on duty. Even at night when you are off you will also consider if drinking would have affected me the next day. Everyone will keep an eye on each other. Take our doctor’s assistant as an example, he enjoys drinking a lot. I would say [to him] I won’t have any problem of you drinking on a Friday night when there is no work tomorrow but drinking will definitely affect you the next day if you have to work the next day. People will know how to control themselves. Some older generation [physicians] don’t really care. [Physicians] of this generation would take it [the effect of alcohol] into consideration.

Interviewer: What does the rest of you think?

1: I was always able to show up at work for most of the time after the year-end parties I used to attend. When I really did fail to show up occasionally, superiors would help me to take over my work at the emergency room. [There is also] juniors and colleagues [who can help you out], so it’s highly unlikely your work will be affected.

2: For those who study management, without speaking of its immediate effect, under the long term influence of culture, has the custom of drinking at a dinner gathering improved a group [in any way].. management.

4: Whether it is good or not about drinking in terms of management. I personally think, like previously said, [alcohol] is a catalyst. I think for the airline industry where they are strict with the pilot who is not allowed to drink within a certain number of hours before their flight the next day and is required to have enough sleep. Our medical industry is somewhat similar to it with a

different nature. If we have to perform a surgery, do an ultrasound, make rounds the work intensity may differ. If I have to perform a surgery I might stop drinking after a certain time. It's essential to stay awake. [You will] get affected. Judgement can be affected by alcohol. Job can be affected. Unlike in the airline industry there is no such thing as restricting access to alcohol for few hours [in medical industry]. It depends on how we make our own judgement [on the amount of alcohol we drink] and we learn to control ourselves in the medical industry. Say if I am performing a surgery tomorrow, there is certainly some restrictions [on drinking alcohol] but there is no particular standard according to my experience. It is not like the strict rule and a fixed code of conduct they have in other industries where high intensity of concentration is often required.

1: I am asking 2. Ever heard anything from your surgery division about being too drunk to perform a surgery the next day?

2: Everyone knows if you go and ask them. Have I ever heard? Yes. Showing up in the morning with face red and smell of alcohol, nobody would dare say anything to him probably because he was some major professor at that time. He didn't cause any medical malpractice. Now everyone can make a complaint about this and things are starting to be different. In the past, I heard people would gossip about who is being the one coming in with the smell of alcohol in the morning, how this person had a gathering the previous night and wound up in showing up like this, they would try to put up with this person. You have to control yourself if it's your own addictive drinking behavior. It's not ok if you always have gatherings to attend and drink all the time. It's ok to take a day off occasionally but eventually if you have always been too drunk to work the next day for too many times, they won't excuse you for doing this.

3: It sounds like it depends on how you define drinking. For binge drinking, there is definitely a 100% influence but not always for dinner gathering as it depends on the amount you drink. Some may drink too much at once while some may only drink 10ml. It has something to do possibly with the way you define how much alcohol you can drink.

Interviewer: The amount of alcohol everyone drinks is different. Do you think [drinking] will affect your professional image or professionalism?

3: It depends on how drunk you are. The smell of alcohol, red face or red nose.. professionalism is definitely affected. It doesn't count if people can't tell you have been drinking, say from some wine tasting. People won't find out if some people happen to drink moderately.

2: It's really strange. Lots of doctors would eat with their patients who have already become their best friends. They also drink at the dinner gathering. Compare what the patient thinks about [doctors] drinking to what doctors think about their colleagues drinking, there are many thoughts. In the society of Taiwan, it's not a bad thing to drink moderately. It is hoped that [drinking] won't affect [us] if we have to perform a surgery. In other words, it's fine when you have a successful surgery. Speaking of drinking alone regardless of

the point of view either from your peers or patients, it's not ok if you get caught being wasted during the day.

1: I have an impression of how people from the surgery division can hold their liquor well. [alcohol] is like a medium [of showing a compliment] and respect to you!

1: I think in the same way as she does. It is allowed to have this drinking culture at New Year's Eve parties or year-end parties and it is not against the social expectations. It's not allowed if you get so wasted in the workplace or during rounds and people would be surprised seeing you like this.

Interviewer: [You] think [alcohol] has an effect on medical practice.

5: Professionalism as you just talked about, it's not about the effect on medical practice and it doesn't matter whatever you do, whether being so drunk has gone against the general public's expectations of physicians. Whatever your sickness is I have to keep it confidential. I can't say anything to the press. And reliability. Expressing my ability, I have to show this ability. Speaking of professionalism, it doesn't matter whatever you do. I think overdrinking can affect professionalism. It's about whether you have shown up on time or even the interaction between doctors and patients. You [a patient] rely on me [a doctor]to provide you the treatment but now it becomes the opposite. [Meaning doctors have to do something in return for the patient who helps cover the fact of doctors being drunk]. Apart from drinking, things that go against professionalism like making your operation a priority or a custom service made specifically for you, it shows how different it is to define professionalism.

Interviewer: The doctor-patient relationship you just talked about….

5: I meant in a doctor-patient relationship, it's like mission accomplished. There is a red line within the law for a pure medical practice. Say if my patient and I are good friends, we eat out and drink after work, the patient lends me money and anything like this. There are actually many people slowly reaching a common ground. There are some unspoken rules such as you can't take your family as your patients, something like this can do harm to your professionalism. It's not something based on your professionalism because it will be irrational and subjective. Drinking and professionalism, doctor-patient relationship that involves[drinking together] is surely affected. Could you make my operation the first to perform as I am going overseas next week? Sure, I can, we drink together everyday. Technically, this behavior is not allowed according to the doctor's guidelines. Apart from drinking, there is also rules saying that you can't have sexual intercourse with your patients. This is a violation. You might wonder why not, if it's my wife then why not? You can't be the doctor of your wife and she has to be referred to another doctor. You have to be serious about controlling the way you drink to avoid violating a regulation.

2: What the patient sees you might be different if your patient sees you drinking and you drink till you drop when eating out at a dinner gathering. All Taiwanese doctors except for the ones from ER are on duty 24 hours. Being your patient's doctor, what [you think] your patient's family would feel if you

drink till you drop at a certain period of time, even if it’s at night. It also occurs to me that what we behave outside might also have an effect on….[how the patient sees us].

Interviewer: You think it will have an effect on professional image.

2: Yeah, it’s like what they show in overseas series, they go for a drink after work, it can have some effects… For Taiwan, it is probably less likely to be …[in this way]

Interviewer: Asia has been influenced by the culture. What’s the meaning behind work-related drinking? What’s your views about it?

1: A lot was talked about. It is for showing respect, creating a friendly atmosphere and making it easy to talk with some doctor.

2: The effects you get from drinking alcohol, get relaxation, one thing is to get some sleep, get addicted is another. For socializing, [the effect] is not really working physiologically but psychologically. Active or passive [drinking]. You do it all because for the group’s sake. There are all sorts of behaviors. Turning down [a drink] is another kind. Refusing to drink [and the consequence of it]is some big aspect worth discussing too. Your supervisor might do something once you refuse [to drink], [you] don’t want to drink for health reasons or being a boss the supervisor can make decisions on everything. Things like these or such behavior and thoughts are all associated with this issue, which were talked about earlier.

5: A socializing tool.

Interviewer: What you others think? About work-related drinking behavior?

3: It’s [mostly] socializing. The year-end parties we talked about, having a communication through each other’s mind, you might be able to get promoted or build a closer relationship with your boss through drinking. [You] take [drinking alcohol] as a certain behavior pattern. Many people who drink and take [alcohol] as a tool are not easily to get addicted.

2: What was mentioned particularly, attending physicians become good friends with their patients. It’s like an opportunity for doctors to expand their social circle and this is a different thought about drinking. It’s a way to enhance interpersonal relationships. A doctor will not be presumptuous at that occasion for the reason of being a doctor. This is another kind of drinking pattern I know. From my experience, there is only alcohol provided at gatherings of year-end parties or welcome parties or when you drink in private. It’s not common to drink till you drop with your best friends, is it?

1: It’s only a medium. There are still some good things about alcohol. It’s not bad for enhancing a friendship or relationship.

2: You will drink till you drop with your best friends only when there is a situation of someone breaking up with someone else or something.

Interviewer: The rest of you, there is a good side, what about the bad side? Apart from the reason of socializing.

2: The bad side is being forced [to drink]or trying to outdrink others because of the rank difference.

1: My wife will get upset!

3: The bad thing about it is a person who can't hold their liquor well can get drunk easily when being forced to drink. If [you] can't hold your liquor well, your face may turn red or asthma might be induced. I think this is something done deliberately to bully someone. Forcing someone to drink is like bullying. People who can hold their liquor well will drink if you intentionally make them drink. It's more interesting to bully those who can't hold their liquor well.

5: The additional fun [is to watch someone] hugging the toilet to throw up!

2: Medical profession is not like the one of a sales person. We drink as to make more "sales" so that the supervisor is more likely to consider a promotion. It doesn't necessarily mean you will surely get promoted if you drink a lot. At least, it's not the case at Linkou [branch], [there will be no difference] whether you drink or not. There might be unspoken rules. Other professions are different. There won't be too many negative effects if you can't drink.

5: Our profession is being the more conservative one. Drinking and its positive or negative influence are related to different professions. People from our profession don't really have to be bold or lower their dignity. I have this friend who does marketing and everyone from their company has to read out a cheesy slogan out on the street of the East district. The first 30 minutes of the event my friend was like there'd better be no one on the road who would know him and it'd be the best if no one could see him at all. It was all different in the afternoon, there was nothing he couldn't do as he thought he had done all the embarrassing stuff after all. This kind of behavior is not required or encouraged in our profession. The bullying act from a group as everyone just talked about is not really happening [in our profession].

Interviewer: You have to control yourself while drinking.

Interviewer: Would you keep the same [drinking] custom if once you have become a supervisor in the future? Would you do the same or change if holding a drinking event?

1: We are hardly being forced[to drink]. Like 2 said, [drinking] helps you say something that you dare not to say normally. Don't mess up the situation.

2: I remember one year of a PGY year-end gathering, there was a prize draw talking about which 2 PGYs had made a donation. It wasn't a good thing that they made alcohol as a donation. They drank half of the bottle of wine at once right at the event and wound up in having a drunk brawl on the shuttle bus. At the end, the passengers lodged a complaint to the superintendent against the PGYs. I think it is sometimes hard [to avoid this kind of thing happening]. For me, I will prepare alcohol and keep the custom, though, I don't enjoy drinking that much. If bullying occurs I probably won't stop it (everyone laughs out loud) as long as it's all within the law and there is nothing depraved!

1: There is a heavy sense of morality within this profession. [You] would take it as a yes if someone doesn't make an obvious attempt to refuse.

5: If I become a supervisor… Having a group was used to be related to gatherings. I heard from my friend, it's necessary to drink once it's over 100, when there is 100 days left. There are many reasons for drinking. You can drink throughout every season. There was a tradition of throwing someone who gets

drunk to a pond in front of the military base entrance, the water there was shallow but this person almost drowned. He was too drunk to get up [from the pond]. This incidence changes the whole [drinking]tradition and no one dares to throw a drunk person to the pond any more. Would you keep this tradition if you were the supervisor? This is a true story from my friend. Got thrown to the pond and almost drowned. You find it impossible, right? The truth is he really couldn’t get up.

Interviewer: Anything to add?

1: I will continue this [drinking] culture!

3: I will, too. It adds the excitement but moderate drinking is a must. Like 2 who would prepare alcohol but never force everyone to drink.

Group 6

Residents (n=6)

Group 6

Interviewer: Please introduce yourself first.
6: 1 is the administrative CR [Chief Resident] for this month. It’s hard to tell about what they like. Then R5 (points to 5), we are R4 the [youngest/ lowest rank] (point to 4 and 6), those two are R3 (point to 3 and 4) and both overseas Chinese from Malaysia, and that’s all.
Interviewer: What are your personal interests and what’s the type of food you like?
6: This is too detailed and I wouldn’t possibly know all about you people.
3: I love to paint and the thing I like to eat is… I like Korean cuisine. (everyone laughs).
6: You don’t sound you meant it.
2: [I]Play basketball or sports. [I like] Eating. I cook occasionally. My interest includes cooking something edible and I think I like Western cuisine.
Interviewer: What about the rest of you?
1: I don’t have any interest in particular, [I am] mostly busy looking after kids. Having [to look after] 2 kids is tiring. My interest includes accompanying the kids! You can’t really have anything [decent] to eat when you have kids to look after, I guess I will just eat the kids.
4: My interests are keeping fish as pets, driving and sports. I have kept a lot of fish. I am keeping sea fish these days. I like spicy food, curry or Korean cuisine is also fine, ha ha. I am ok with any sport, playing basketball, swimming, gym and that’s it.
5: My interests are performing surgery, any sports combined with travelling, skiing, swimming and I cook occasionally.
Interviewer: Any type of food you like?
5: [Foods] with strong taste.
Interviewer: It seems many of you enjoy food with strong taste. Do you drink alcohol when eating out? [Do you have meals] with alcohol? On what occasions?
2: Something like year-end parties or medical societies.
Interviewer: What about during the time when you eat?
1: Eating on my own?
Interviewer: Anything. It doesn’t matter if it’s during the time you usually eat or not.
5: We usually have meals without alcohol.
6: When eating out with my classmates or attending a wedding banquet or something.
Interviewer: [eating out] with friends or [attending] a wedding banquet. A wedding banquet and a restaurant?
6: An outside occasion.
Interviewer: How often.. how many times a month?
1: Hardly once [a month], once every 3 to 4 months. Was your question about how many times a week do we drink?
Interviewer: Or the number of times [you drink] in a month when you have your meals. There is no limit. We have open questions. What about the others?
5: 3 to 4 times a month.
Interviewer: On what occasion?
5: Eating out with friends.
Interviewer: At a restaurant?

5: Yes, a restaurant. (laughs out loud)

3: Did you mean to say some place else like a drinking pub?

5: No, it was Karaoke bar.

Interviewer: What about the rest of you?

4: Not very often, once a month and sometimes less than once.

Interviewer: On what occasion?

4: Eating out. A restaurant.

6: I used to drink more before but not anymore when I have started working, otherwise I won’t be able to work the next day, so I drink once or twice a month at the most.

Interviewer: Once to twice a month, where do you drink?

6: Outside and at home.

Interviewer: Could you share any experience of drinking related to work? What was the situation like?

2: The year-end parties held at places. Say if an invitation is sent out for the year-end party tonight and it’s ok if you don’t stay there to eat as long as you have prepared the red envelope. You are also told that there will be alcohol provided on the table and you know nothing about what goes after. It’s like you have to pay [for the wedding](with the red envelope), get forced to drink and it seems this is all what a wedding is mostly about.

Interviewer: [A gathering held] at the division? Could you give more details about what it was like at that time?

2: The year-end parties held at all sorts of places.

Interviewer: Could you tell us what it was like at that time?

2: I can’t remember. (Everyone laughs out loud). Hand in the red envelope once [you] enter the wedding.

Interviewer: What about the rest of you? Please tell us about any work-related drinking events such as year-end parties or spring receptions.

3: I don’t often go to dinner gatherings now. I don’t drink because there is work. [I drink] occasionally and usually get forced to drink.

Interviewer: Could you tell us more about it?

3: A group of rookies were forced to drink. I can’t drink and I am allergic to alcohol. I was hiding in the back. I would hold the alcohol in my mouth before spitting it out and I never drank down the alcohol.

6: God! A big secret!

Interviewer: Where was this?

3: Like the [gatherings held by] medical societies, spring receptions and year-end parties.

Interviewer: Does the rest of you have any unforgettable experience? Describe what it was like at that time. Every doctor should have attended [this kind of gathering event].

1: Not many people from our division are into drinking. There is no such drinking custom.

6: Exactly!

1: Most of the time I would go to a year-end party which my boss won't attend. I tend to drink more at the dinner gatherings held by other medical societies outside [the hospital].
Interviewer: What was it like to be at a dinner gathering of a medical society?
6: There are elders.
1: After few presentations of cases, people would sit down and get ready to make a toast. People would start to leave their seats when the fifth dish was served. It was a mess as everyone was making a toast and drinking everywhere.
Interviewer: Whom were you drinking with?
1: The neurosurgeons from other hospitals.
Interviewer: Where they [your] peers or teachers?
1: We were not in the same year.
Interviewer: What about the rest of you? Please share more [of your experience] or you may also discuss privately.
1: We are from different years and will never attend the same medical society event.
6: Same as us, that's what a medical society event is like.
Interviewer: Apart from the events of medical societies, the rest of you have also been to such a similar event?
4: Pretty much. Young and lower rank people have to take turns to make a toast and it is to ensure that each of them has taken the alcohol. The event for the medical society is once a year.
Interviewer: Anything else, apart from medical societies and year-end parties?
5: I have never been to other events.
6: [My] life is simple.
Interviewer: Tell us about what it is like at that time, what was the atmosphere like? The interactions.
1: Mostly it was like, we were all students at that time and had to make a toast to the teachers from every hospital. [We] drink a toast to each other. A person whom you drank a toast to would come to you and make a toast to you few minutes later. It's like to make each other drink continuously. We would hide in the back watching them doing so because we didn't like drinking. At the end, everyone was so drunk with their faces appearing red and we left immediately once they got drunk (ha ha ha).
Interviewer: You were…[watching them from behind].
1: Yeah, watching them drink because I don't like it [drinking] personally.
Interviewer: As we talked about being forced to drink… who were the people involved?
1: Students made the teachers drink or teachers made the students drink. This cycle goes on for several times.
Interviewer: Have the others here been in the same situation?
6: It's common for everyone to be in that situation if attending such event like that of the medical society.
Interviewer: What about… what do you think what it was like at that time?
5: It was exciting. (haha)
Interviewer: Tell us more about it, or any other events to share, or any experience you have heard of.

6: If it’s [anything] related to the medical field, some CR seniors of other hospitals were forced to drink and got seriously drunk at a dinner gathering, I heard that the next day, they had been given intravenous drip for 2 consecutive days without being able to do anything!

Interviewer: Were they CR?

5: That is unlikely to be seen at our division. This kind of thing is more likely to happen at the division of ENT or urology. At ENT division, it is always their goal to make the R1 drink till they drop at the welcome parties. Their R1 freshmen always wind up in the emergency room. It has been their tradition for many years. [This made them] get to decide on their specialty early in the emergency room instead on the night of their welcome day.

Interviewer: Others of you who have been to the welcome parties, please tell us more about what it was like at that time.

3: Everyone didn’t know each other well. [Someone] would get on the stage and dance before starting to make a toast. An R1 was expected to drink 5 glasses but I held it [alcohol]in my mouth in the same way I did before, all the alcohol of 5 glasses was in the mouth and spat out [afterwards](Everyone laughs out loud).

Interviewer: You were R1 at that time, who were you [drinking] with?

3: R1, [I] took 5 glasses. A major boss and some VS. Everyone was like a kid after drinking.

Interviewer: Did the rest of you feel having similar or different welcome parties?

5: The welcome party we had on that day was at the Tang spa (a theme park), no one drank because all people were there for the hot springs.

Interviewer: What about the rest of you?

6: We were performers at that time and we did drink after the performance. The stage and the place were small.

5: The year [of the welcome party held] at Song-Chieh-Chin (a seafood restaurant serving fried dishes).

Interviewer: Do you drink at a restaurant that serves fried dishes? Could you tell us more about what it was like at that time at the restaurant serving fried dishes? [Were you] with peers?

3: There were 9 R1 and everyone who showed up was nice and seeing each other for the first time. An R1 had to get on the stage to perform once the teacher finished their speech. After the performance, the R1 made a self-introduction and started to line up on stage to make a toast. Everyone was required to drink 5 glasses. There was not enough budget for getting wines of good quality and the glass used was small. Then they started to eat until the third or fourth dish was served and they would have to make a toast again to every table, but everyone was quite rational at that time.

Interviewer: How was the interaction?

3: It was friendly. Unlike the medical society event we had, no one was forced to drink.

6: The one before..(agrees with 3)

Interviewer: You talked about drinking 5 glasses? You think it was ok?

3: I didn’t do so. (ha ha)

Interviewer: The part about being urged to drink as you just said, anything like being forced to drink?
5: You just had to drink and there was no way of refusing.
3: “Now let’s welcome R1, something something (their full name) to the stage” and a CR would be standing nearby. Every glass out of a total of 5 had to be finished one by one. (Everyone giggles)
6: Your supervisor.
Interviewer: Was there a lot [of alcohol]?
3: A small glass. A tiny small whisky glass of pure whiskey.
6: How did you find the right time to spit it [alcohol]out?
3: [I] just talked with it.
5: It was stored in the cheek pouch. (ha ha)
Interviewer: What about you? It was no drinking for 3 as we just discussed. The rest of you?
4: I did, but [I] can’t remember how many glasses I drank. It was also to make a toast to the teachers and it was ok as no one was forced.
6: As an R1 at the welcome party, [I] made a toast to the teachers from the division of the future specialty that I intended to choose. It was a friendly interaction and no one was forcing others to drink.
Interviewer: Did everyone tend to drink more at the events of medical societies?
4: It was more like being forced to drink. Once at the welcome party and there is this medical society event once a year.
6: The people outside [of our hospital] are more open-minded.
4: [Those people] can hold their liquor well.
Interviewer: You talked about being forced to drink, [were you forced] by an attending physician, a teacher or your peer?
6: It was usually the supervisors who would ask us to toast what superintendent of some hospital. He[the superintendent] is being this…[a superintendent], how could you not to make a toast [to him]?
Interviewer: Do you think it was toasting or being forced to drink?
1&6: It’s the same [thing].
4: It sounds nicer when you call it toasting.

Interviewer: What do you think about the teachers or peers whom you normally work with if you see them drinking at an event? Would you change the way you see them normally?
1: I think we won’t see any difference because the people whom we drink with are the teachers from outside and we don’t know them well, and the teachers from our division don’t drink at dinner gatherings.
Interviewer: Any year-end parties or…
1: [I] go out and drink together with nurses, ward nurses.
Interviewer: They are your colleagues?
1: After drinking, they could get as hyper as they normally would. There is not much difference.
Interviewer: Any unforgettable experience or experience of year-end parties?
1: I’ve only been once.

2: It is only once or twice for me.

Interviewer: What did it feel like and how was the interaction between each other at that time?

2: It's not likely to drink a lot at a year-end party. (Everyone agrees)

6: We sang at the karaoke bar, a tacky karaoke bar.

Interviewer: But you still drink at the year-end parties, it's just that..

6: It's different for everyone. The experience I am going to share is different from that of others, there were lots of outdoor activities at the hospital I used to work at. They often held many dinner gatherings or banquets held even at the hotels, many drinking events. It was exciting at first to be able to know many people through these events but eventually my thoughts about them were changed, I felt these people were doing nothing meaningful and started to take a dislike to those events.

Interviewer: What did you think? It gave you a bad impression.

6: They[the events] were way too often and the drinking situation was turning sour.

Interviewer: Would this affect the way you normally see a person (who drinks with you)?

6: It wouldn't have affected so much, probably only a little. But the thing is that I wasn't close to those people whom I met from those events. It wouldn't be fair to make a judgement based on something so little we had known. Those people were never our colleagues.

Interviewer: What about the rest of you? Would seeing someone drinking at an event changes the way you think about them normally?

2: Probably it wouldn't affect me much? There is no difference. It's only to eat out and have few drinks. There might be some people in a drunken fit after drinking, and you wouldn't want to perform surgery together with this person after realizing what it was like when they were in a drunken fit.

Interviewer: You had a kind bad impression.

2: It's not about being good or bad. It's only like one of his (the person in a drunken fit) characteristics.

Interviewer: You think it's one of his characteristics.

2: There is no good or bad about it. It's not like he would come to work drunk everyday. It's not my freaking business if he takes clothes off and hugs people around when drunk as long as he doesn't hug me.

Interviewer: Does it change the way you see him?

2: Not really, not much. It's common to see someone like this when they are drunk.

Interviewer: You think there is no change [in the way you see him].

2: It's nothing special.

Interviewer: What about you, 1?

1: I have never seen anyone drunk from our division. It's probably not so bad even if I have ever seen one. Public and private matter are separate. It doesn't matter whatever you wanna do in private.

Interviewer: What about the rest of you? Have you ever had or seen anything like it?

3: Like 1 said, public and private matter are separate. Whatever you do outside office hours has nothing to do with me. My father-in-law likes to drink too and often ends up in a drunken fit, but it doesn’t change the way I see him.
Interviewer: The usual time you get to spend with him, or during work.
3: No, it won’t [change]. The relationship with him when he drinks is one particular type of interaction while it is another when he doesn’t.
Interviewer: Other people here?
5: You show different behavior and attitude between work and leisure time. This is why how someone acts after drinking doesn’t affect the way I see them. It won’t make me feel like learning things from a person who has a good drinking behavior but is bad at his job.
Interviewer: 4, you?
4: Drinking is what he chooses to do. What he does normally shouldn’t be affected. [I] have never seen any teacher from our division getting drunk.
Interviewer: What about colleagues?
4: None. Only a long time ago, I guess. [I] drank a lot with the people from other hospitals when I was an intern. Apart from this, there’s no anything like it.
Interviewer: What did you think at that time before?
4: We drank after work. Once you are drunk there is a different way for others to interact with you.
Interviewer: Would the person you drink with urge you to drink? What would it be like?
4: Telling me not to drink or something? No.
2: It means forcing you to drink more.
4: Probably not. If they do [ask you to drink more] you won’t necessarily have to do so.(ha ha) I think it’s ok, [because] mostly likely [they are]more drunk than you, those who ask you to do so.
Interviewer: Would you urge others to drink more or things like that? Probably [doing that to]your juniors, would you?
6: We hate being urged to drink, so, no, it’s not going to happen, unless for other [special] occasions.
Interviewer: What about you two?
1: There’s no being urged to drink. If someone comes to you and say let’s toast, you just drink with them. You drink when they come or you can avoid drinking when they don’t.
Interviewer: There is experience of being urged to drink.
1: It’s not really being urged to drink. You just drink when someone comes to toast you. There is usually many tables [to go] and they won’t stay forever at your table. After drinking and toasting you, they go to the next table instead of staying behind to drink with you [at your table]. It’s not like we are some kind influential people [to drink with]! (laughs)
Interviewer: Ever urged anyone to drink?
1: No. It takes turn for one table after another. No one would say “hey, drink more.” If someone comes to your table and says “Come on, let’s drink more tonight and get drunk a bit more” they don’t really care whether you have drunk or not. They move on [to the next table] after toasting you and that’s what it’s like most of the time.

Interviewer: 2, what about you?
2: Pretty much the same! [We attend] the same events.
Interviewer: Same events but people may have different thoughts about them.
2: He sat next to me and we would get away together if something was wrong.

Interviewer: Would you arrange a drinking event if you are to hold a dinner gathering?
6: Who is this holding a dinner gathering?
Interviewer: Yourself. Let’s talk about the gatherings held at the division.
2: There won’t be alcohol provided at most of the divisional gatherings.
6: That’s true!
2: We eat mostly at the places in the hospital which doesn’t sell alcohol, so we won’t feel like getting any to drink. The dinner gatherings at our division are mostly about the people form our division eating out to have some discussion and there is no time for drinking.
Interviewer: What if it’s to hold a gathering like year-end parties or welcome parties, would you arrange [a drinking event] for some other activities?
1: This kind of drinking arrangement, it is nothing people from our division would think of.
Interviewer: What about the rest of you? What would you do?
5: It’s to eat and have a chat at a dinner gathering, then dismiss [afterwards]! (ha)
Interviewer: Will you want the gathering to be more exciting? What would others of you do?
2: If I am holding such an event I will[prepare alcohol]. I will regardless of whether people drink or not. We will get to that when we find out if there are people who really drink.
Interviewer: You will prepare alcohol. What about the rest?
3: I will too. Probably [I] will prepare red wine, whiskey and beer. It feels classier as the price for red wine is higher. (ha ha ha) Chat and drink some wine.
6: It was usually the red wine, the one from hotels, red wine and white wine, if there was alcohol provided at social activities held by our division.
Interviewer: You’ll prepare alcohol. 4, you?
4: I probably should if I am the one holding such an event! Yeah. It’s necessary as there are still people who have a habit of drinking. Being a host, you have to provide a proper hospitality for your guests. It’s thought necessary to prepare [alcohol].
Interviewer: You will prepare alcohol. Basically, most of you will do so, [doing this] for the guests or….?
4: For me, it is for the guests. I personally don’t know which kind of wine is good to drink or have any particular wine I like. Anything regular will do and it’s medium-priced, not too expensive, and doesn’t look too bad!
Interviewer: For those who prepare alcohol, will you toast someone or urge someone to drink?
6: We will see if anyone has started drinking.

5: If there is no alcohol we will still make a toast with juice. It is a custom, holding a glass and clinking of glasses everywhere [regardless of] whatever in the glass.
3: Probably a bottle for each table.
Interviewer: You will prepare alcohol for whoever wants to drink. Everyone normally doesn’t drink that often apart from dinner gatherings.
3: Occasionally [I] drink with my father-in-law but can’t drink too much. (ha ha)

Interviewer: Will drinking at a gathering, as just mentioned, affect medical practice? What do you think?
6: There should be no one who goes straight to work right after drinking!
1: Dr. Y does. He had showed up drunk for an operation. I forgot whether he had performed that surgery or not at that time. He showed up drunk saying “ this patient needs an operation.”
Interviewer: You think there is an effect on medical practice then.
1: That was the only incidence after so many years, I haven’t seen any others.
Interviewer: What effect, if any, does the rest of you think?
3: Once I was a PGY on duty and ordered lunch delivery together with the nurses, there was alcohol in the sesame oil chicken noodles we ordered (ha ha). My face turned red and I was a little drunk after drinking the soup [of the noodles]. I lay in bed probably for 2 to 3 hours before I could get up. I think never drink while at work(laughs).
Interviewer: You meant you got drunk from sesame oil chicken noodles?
3: Yup!
Interviewer: It had affected your work the next day?
3: It affected my work on that day! The nurses knew it was all their fault!
6: This is really funny.
Interviewer: This was your personal experience, what about the rest of you? Your daily medical practice? Would your professional image be affected?
Interviewer: 6, what do you think?
6: Don’t drink till you start to talk crap. It’s obvious to see you turning red all over. Patients can probably accept if you are able to behave normally. Abnormal behavior… such as becoming extremely talkative or saying weird stuff that others find it hard to understand, or getting red all over the body [is a pretty obvious abnormal appearance]. In this case, the patient will have doubts about your professionalism.
Interviewer: You think this will affect doctor-patient relationship then? What does the rest of you think? Your interaction with the patient will be affected?
1: Imagine the situation when you get drunk in white coat, but I guess there is no one [who would do this].
6: But no one would do such thing!
Interviewer: If you drink the day before….
5: You will just look pretty tired the next day if you do drink the day before. It’s the same as looking pretty tired the next day after the day on duty. (ha ha)
Interviewer: 4, do you think it [medical practice]will be affected?
4: Don’t drink while you are in white coat.

Interviewer: [We’re]talking about the time during drinking events at gatherings.

4: It won’t be affected, I guess. [I still] go to work the next morning, usually [I wouldn’t] be affected the next day. It’s ok if you drink in an ordinary way, unless it’s for someone who drinks till they end up in the emergency room.

Interviewer: You think there is totally no effect [on medical practice] then?

4: I think it’s ok as long as you do get enough sleep on the day before.

Interviewer: What if you have been drinking on a long-term basis, drinking at gatherings.

6: We don’t drink on a long-term basis.

Interviewer: Most of you are not a long-term drinker.

1: Being a doctor, you won’t make yourself become a long-term drinker otherwise you won’t be able to perform surgery. You won’t make yourself like that as a doctor.

Interviewer: Does it only happen when you get drunk? Or you will always get affected regardless of you drink more or less.

5: Some people drink on a long-term basis and their hands will shake without drinking for one day. Doctors are also less likely to make themselves become a person like this.

1: Most doctors won’t drink as much as in binge drinking. The doctors having a habit of drinking a bottle of beer at home after work is probably what you see the most for doctors who drink on a long-term basis. They never get themselves drunk and [there is] no relation between what they do after work and their image at work. Work is affected, possibly when you get too drunk to wake up and can’t make it to make rounds. But it looks no different when you do get a good rest before the next day.

Interviewer: Did you just say being unable to make rounds in time has not too much of an effect?

1: This might leave a bad impression on the visiting staff. Failing to show up to make rounds doesn’t not have too much effect on patients.

6: Like what was just said, [the patient] wouldn’t know [the doctor] had been drinking.

1: It’s most likely the boss would think it is as a bad working attitude but it won’t have any influence on professional image of a doctor nor affect the judgement at work.

Interviewer: Professionalism.

1: There is no effect. The only difference is in what the superior thinks about the [your]working attitude but no difference in your professionalism.

Interviewer: [You think] there is not much influence. Since everyone has some experience of drinking, what does everyone think of the meaning of work-related drinking behavior? What are your views about it? The drinking behavior.

5: It’s generously thought by others that surgeons like to drink no matter whatever the occasion is. Whether to drink or not, it depends on the occasion.

Interviewer: You meant for the drinking behavior? What is the influence of drinking behavior on doctors? What do you personally think? Whether it is for socializing or because of the culture?

1: There is this apprenticeship system [commonly seen] at the division of surgery. The relationship between a mentor and an apprentice. And they [the teachers and apprentices] become closer to each other without feeling a wall between them when they get together to drink after work. I think everyone is able to make a clear separation. You do what you do at work and everyone can get closer when drinking after work, these 2 don’t mix together. You are back to the mentor-apprentice relationship when you return to work. Whatever someone shows to be more intimate to you at night (while drinking) than they used to is never going to happen at work. Everyone can make a clear separation. [Drinking is] a way to reduce stress after work and to feel closer to your teacher.

Interviewer: To build a closer relationship with your teachers.

Interviewer: Do the rest of you think the same too? 1, is it because you normally have a lot of stress when you talked about stress reduction just now?

1: It was ok actually.

Interviewer: The source of your stress?

1: There isn’t a lot of stress actually. People form our division don’t drink that often.

Interviewer: What does the rest of you think? Like what 1 had talked about things like stress reduction and getting closer to each other.

5: I love working the most! (laughs out loud) I love doing reports!

Interviewer: What is the meaning of drinking behavior? What do you think it is?

5: For speaking in terms of [drinking]in the public, it helps make everyone become closer because there is not much inhibition or stress left after drinking, and people will become hyper. Everyone who gets more hyper tends to get closer to each other.

Interviewer: What about the rest of you? What is your personal view on this? What do you think?

2: About making a toast, what it means…. It depends on the occasion. If few visiting staff who know each other well take their students to a Western restaurant it’s considered ok for them to order some alcohol to drink. It’s ok for a boss to treat [them] to dinner and order alcohol for everyone to drink. People attending gatherings held by medical societies don’t actually know each other well! It’s therefore hard for them to talk much to each other under that circumstance. Some people will try to build up a relationship by toasting, it’s like to say hello to meet first then start chatting, and break the first barrier. It’s not like as easy to start chatting with someone you don’t even know as a sales person does. This is how it is. It’s only that some people focus on drinking and they drink till they drop. Some people are only there for a chat and they think alcohol can help them to start a conversation easily. It (the meaning of making a toast) all depends on how you drink.

Interviewer: You talked about building up a relationship by toasting?

2: Let me put it this way, the people from the drinking events we attend are often from the same field of work, some are from the same division while some are from different divisions. I think attending these inter-divisional gatherings

always helps your career in some way by getting to know more people to enhance your interpersonal relationships [with other doctors or teachers].

Interviewer: It helps you become closer with others. You talked about there is also something like being urged to drink?

2: It’s normal to urge others to drink. It will be weird if there isn’t.

Interviewer: Is it the elders or teachers who would do that?

2: It’s true there is often more elders who would attend those events. Those who are younger don’t actually drink.

Interviewer: Senior elders?

2: That’s what I remember, they are usually 5 or 10 years older than us. There are not many doctors who have to stay on duty in the hospital can drink till they drop and skip work the next day. CR is the only person who is qualified to urge others to drink. For visiting staff who have known each other for 5 to 10 years like us, we never urge others to drink.

1: Peers won’t urge each other to drink because we know the supervisor would do this to us and we would help each other to save drinking capacity for later.

Interviewer: 4, what you think?

4: The meaning…It’s inevitable to refuse if it’s the teachers who urge you to drink. It is a courtesy otherwise you would feel bad [for not doing so].

Interviewer: What do you think? What’s the meaning behind it? Do you think it is ok?

Interviewer: To accept something like this?

4: Yeah

Interviewer: Will you refuse to do so?

4: For me, I don’t really say no to it. I don’t find it annoying nor have any special thoughts about it.

Interviewer: 6, What are your thoughts about it?

6: I think it’s ok as long as you can separate this from your work.

Interviewer: Do you mean to prevent drinking at work-related gathering from affecting work?

6: It’s ok if you can manage it well.

Interviewer: You don’t think much about the meaning of it, do you?

6: No, I meant it depends on whether you can manage to handle it well, otherwise it’s bad for others and yourself.

1: What do you mean by handling it well?

6: Let’s say going back on duty after you get drunk, it’s like you go out to drink while on duty and come back to work and yell at your patients after you get drunk. I have seen someone like this from a previous encounter, this gives me a ……. impression of him, but instead if I happened to meet him and see him drinking at a social occasion where drinking is only for socializing, courtesy or making a closer interaction, it will be no big deal and it’s only considered his personal habit.

Interviewer: You think it helps build a closer relationship like what others said just now.

5: It’s like a way to make a start [in conversation] when people don’t know each other well. It’s like a key to initiate interaction by toasting.

Interviewer: You think it helps build up a closer relationship. Will you continue to do this if you become a supervisor one day?

2: It’s hard to avoid if you are from the surgery division.

6: I will surely prepare alcohol. It’s different for everyone though. It’s a courtesy to prepare alcohol but it’s up to everyone to do whatever they want.

Interviewer: 4, what would you do if you are a supervisior?

4: [It depends on] whether there is money or not(haha). Whether you are able to do something you want depends on whether you can get approval from your boss. It is always the rule! Being a supervisor doesn’t mean you have the most power because there is always a supervisor for a supervisor! The second thing [to consider] is the budget if there is no other opposing opinion [from your boss].

Interviewer: 3, what do you think?

3: I think drinking [culture] comes from Japan and Korea (laughs). It’s hard to see this kind of drinking culture overseas apart from Japan and Korea. You may wonder why every medical society held appears solemn and the alcohol provided is also better. In Taiwan, urging others to drink is seen as common as what you see in Japan and Korea where the younger ones have to toast elders for showing a respect to someone, this is something what I think pointless. We can only do as told if this is what the drinking custom is like. I think… in private.. I think the way you drink in private or at social gatherings has not much relation to your work. At work, you are judged by your abilities, drinking is another thing and your supervisor will not diss you or anything if you drink. No, this won’t happen. You have the abilities and can do your job well even if you can’t drink. Talking about the bad side about binge drinking…

Interviewer: If you become the supervisor?

3: I will prepare [alcohol] but people are free to decide whether they want to drink or not. They can either open the bottle [to drink] or leave it for recycle. There won’t be any occasion made specially for toasting. You can drink a toast with whatever, tea or juice. [If I am] the supervisor for holding this dinner banquet, I will go to every table to make a toast. [I] won’t care what drinks others make a toast with because I don’t like to see [anyone]getting super wasted and making the situation turn messy.

Interviewer: 3, You will prepare alcohol for toasting but won’t ask others to drink when you become a supervisor, is this what you meant?

3: Yup!

Interviewer: Lastly, has anyone got anything to add? Anything missing or occurs to you?

Group 7

Residents (n=5)

Group 7

Interviewer: Please tell us about your job position, interests and favorite type of food.
2: We are all residents.
Interviewer: Please tell us your names, interests, job positions and favorite type of food, so we can get to know each other.
2: I like to play video games and shopping. I don’t have any food I like in particular, anything will do, nothing in particular I like, you can say that I am not picky.
3: Interests are sleeping and going out to have fun, anything. About the food I like, I am ok with anything yummy.
5: Interests are novels, movies and the food I like is pasta. I don’t really like desserts. I don’t actually drink that often. Most of the alcohol, like beer, doesn’t taste good to me. I won’t specifically buy alcohol to drink. I only drink when it is needed to drink at a social gathering with someone else and I will only take a sip or two.
4: My interests are playing bridge and listening to music. My favorite food is Japanese cuisine.

Interviewer: It seems everyone is into a variety of food, from Japanese to Western style. Do you all have the habit of drinking with meals? Do you have alcohol to go along with your meals?
5: No.
3: Probably with late night meals or snacks.
2: Most likely fried dishes. There is also alcohol provided with the set served in a Western restaurant, yeah.
Interviewer: At a Western restaurant.
2: Or drinking beer at a restaurant serving fried dishes.
Interviewer: How often do you drink? Once a month or a week?
2: 1-2 times a month.
Interviewer: What about the others?
3: 3-4 times a month if drinking in private is also included.
5: It’s not many if only 3 times a month.
4: Sometimes [I drink] during gatherings.
Interviewer: Where was that?
3: At a restaurant.
Interviewer: 3, you drink 3 times a month, is it at home?
3: I usually drink when there is a dinner gathering at places like a restaurant serving fried dishes, a Japanese-style bar, all-you-can-eat or BBQ restaurants.
Interviewer: Whom do you eat with?
3: Classmates
Interviewer: Workmates?
2: Yes, workmates.
3: Yeah, workmates.

Interviewer: Could you share any work-related drinking situation? What was it like?
2: Does a mentor-gathering count?
Interviewer: Is there any? A gathering within a division.

4: Whom you invite depends on the occasion. You might invite your workmates, say a dinner gathering for the residents and the visiting staff, a bigger one even includes nurses.

Interviewer: Was that the divisional gathering?

5: It's closely related to whom are those invited. For people with a job rank like us attending a gathering held by the neurology division, we can meet some teacher who has invited a doctor to the gathering and whether we will drink or not depends on this teacher. Like 4's teacher who has known a lot about winetasting always prepares some good quality of wine to share with us. They have something like this for sure. For my mentor, s/he is nothing like that.

Interviewer: 4, Could you describe what it was like? It sounds like you have some interesting stuff to share.

4: [Different wine is provided when] Having a variety of meals such as red wine or white wine provided when eating at a Western restaurant, there is beer or Gaoliang when eating at a seafood restaurant or a restaurant of Taiwanese or Chinese cuisine, our teacher will also prepare alcohol if we all go to cook at a friend's place.

3: Alcohol prepared to share with everyone.

Interviewer: Is it a divisional gathering?

4: We don't belong to any division yet so it's not likely for us to join any divisional gathering. We have our own mentors. [We] attend invitations from some particular person and eat out together, there might be alcohol or no alcohol provided.

Interviewer: What about you two?

2: Mostly mentor meetings.

3: Like what 4 said, sometimes the nurses are invited too.

Interviewer: Would you tell us more about it?

3: Like 4 said, there were residents, interns, visiting staff and nurses to eat and drink some alcohol together, something like that.

Interviewer: There were nurses, doctors and mentors.

2: Mostly the mentor meetings[are what we attend]. The mentors take [you] to eat out and probably have some drinks. Mentors and those [students]from year 1 to year 7, and sometimes it's more fun when there are 2 mentors, we usually have some alcohol to drink.

Interviewer: From year 1 to year 7, so there were juniors.

5: I attend even few events than they do because I am always with a group of other students and the teacher in charge of our group. I have never been to [an event] of nurses or residents. It is simply a group of interns or clerks and students still studying at school. We don't drink for most of the time, it is a pure gathering and we don't really drink alcohol. We are usually at the places where no alcohol could be possibly provided, such as a restaurant serving ginger duck, Ikari (a coffee shop). For 90% of the time, our mentor meetings were held at Ikari on the basement level. The two mentors are surgeons and always too busy. They like to go to Ikari where a meeting can be finished rapidly in one hour. It's highly unlikely [for them] to drink alcohol because

[they] will have to work at the outpatient later or things like this. [The only break] at work is probably only the short period at lunch.

Interviewer: Others of you had talked about drinking alcohol?

4: You won’t be forced to drink, it’s totally up to you, you drink only when you want to. The amount they drink is not much, they know clearly how much they can drink up to, but basically, it’s rare to see someone drinking till they get drunk, hardly any.

Interviewer: What about the rest of you? Do you drink a toast? What is it like?

3: We toast if drinking with nurses. [You] have to be precise when asking about drinking a toast, did you mean who makes a toast to whom or what?

Interviewer: Yes, do you make a toast to each other?

3: It’s a must!

Interviewer: Is there a pattern? Say you drink one glass while I drink 3 glasses.

3: Not that far-fetched! It’s usually the visiting staff who will make a toast to a group of residents and it’s most likely he/she will drink one glass in response to every glass from each person.

Interviewer: Is it a table for all the visiting staff or…?

2: There weren’t many people.

3: Interns sat at the tables for all doctors. Tables for doctors sitting together and other tables for nurses.

Interviewer: Anything unforgettable or interesting to mention? Give us an example please.

5: [People from]The events we attended were all pretty reserved. Some silly stuff that might occur since..

2: Does breaking the glasses count? Ha ha.

Interviewer: Things like how you make a toast.

1: The seniors would make a toast before… Sometimes the teachers were more generous and there were lots of others from other divisions, lots of people were invited. The seniors would take the younger ones to make a toast and introduce to us about who is in what year and who is the senior from the certain resident year, and things like this. There wasn’t a lot [of alcohol]to drink though. Just one glass of alcohol was enough to make a toast to all the people. You only had to drink a sip or very little.

Interviewer: Would the seniors take you to drink with other seniors or teachers?

1: The seniors did.

Interviewer: [You] only had to drink one glass and..

1: I only had one glass to drink, others might have taken some refill. I only drank sip by sip.

Interviewer: Did others make a toast to each other?

1: They did. Those who know each other well will do that.

Interviewer: Was it to the colleagues or seniors. Any teachers involved?

1: The teacher was there too and he/she would tell us to drink. He/She was more open-minded before but is more conservative now. He/She doesn’t ask us to drink that often as before.

Interviewer: So the teacher would ask you to drink?

1: Only if there is alcohol to order. We will order the drink if the alcohol we see looks pretty nice at the place we go or something like this.

Interviewer: Does the rest of you have something similar to what 1 said?
3: Any drinking situation or the situation where the visiting staff would encourage us to drink?
Interviewer: Yes! The visiting staff would urge you to drink, wouldn’t they?
1: It’s not urging you to drink but it’s only to drink when you can.
3: Basically, the visiting staff would say drink whatever you can drink up to when there is alcohol provided to each table, they won’t force you to drink but you can keep on drinking and keep on drinking as you feel like it.
5: They don’t necessarily want to have a blast drinking with you. Instead, they are trying to tell you they have treated you to a dinner which they have paid a lot for, such as there is steak or alcohol to order and things like that, or such and such. There is pricey food to choose and they hope everyone can have fun. It’s not like they won’t stop until you get drunk or the drinking custom they have in other industries. We are often tired already before we attend the events. Everyone tries to stay awake to interact with others. It’s different from being urged to drink for building up a closer relationship as you would encounter outside [of the hospital]. It’s more like out of courtesy, a gathering for adults where there should always be alcohol provided. It’s more like this!
2: I feel that you have to ask people from surgery division about this. It’s said a tradition for them to urge the new R1 to drink.
3&5: It’s a joke, isn’t it?
3: I don’t think they will do that. They just love to drink but won’t force you to do anything.
Interviewer: Have you ever been to spring receptions or year-end parties?
2&3: Not yet.
5: We won’t get invited.
Interviewer: You haven’t had any so far.

Interviewer: As we talked about drinking with teachers and seniors, when they drink at a gathering, do you think they look different from the way they are at work? Does this change the way you see them?
3: Like 5 had said, [they] won’t be in a drunken rage or anything. Some doctors are friendly even when they are not at a dinner banquet. Those two mentors I have seem like serious people to others. They are not like so but friendly instead when they are at a dinner gathering. It’s nothing to do with alcohol. They don’t drink and they look more serious at work.
Interviewer: What about the rest of you? Teachers or seniors are more serious at work and will you see them differently if seeing them drink at a dinner gathering?
1: They are what they appear; there shouldn’t be much difference.
2: I think nothing much about it because I haven’t been to any [drinking gathering].
3: They won’t drink until it’s gone out of control. They seem reasonable at work. The patients’ lives depend on them and they won’t act frivolously as if they are drinking.
Interviewer: What about when [drinking] with the seniors?
2: I think it’s ok.

3: They won't be in a drunken rage.
2: I think it's ok as they are no different from us.
3: If they do get too drunk they would only fall asleep or keep on talking and talking, you won't see them misbehaving.
Interviewer: Do you feel they are different?
3: No.
2: Who had ever been in a drunken rage? It makes more sense to interview XXX.
Interviewer: Seen anything ridiculous or heard of any stories?
2: Someone from our class had gone completely naked at our hostel after drinking, XXX.
Interviewer: At a dinner gathering?
5: In private with some friends.

Interviewer: We talked about drinking at a dinner gathering; do you think it will affect your work normally?
2: No. It's ok as long as you know how to keep your private life separate from your work.
4: You don't drink at work!
Interviewer: Before or after work.
4: Drinking helps relax.
1: To reduce work stress, it should be ok.
2: Something happened because of[someone] drinking before. It's ok [to drink]if nothing happens.
Interviewer: What's the influence of drinking at a dinner gathering on your daily medical practice?
2: Nothing at all!
3: It's alright. You won't drink yourself until you are unable to work the next day if knowing you have to work the next day.
Interviewer: Have you ever seen anything like it?
3: No, not really. Everyone is a doctor and we won't let our own private behavior affect our judgment.
2: There might be something like this but it's just that we don't know or haven't seen it.
3: At least we haven't seen it.
Interviewer: Have others of you seen anything like it? -where medical practice is affected and hence the patients?
5: Getting up late and being later for work the next day, though I don't know about how late he was but he was late for sure. We called him but he didn't get our calls possibly of a bad reception, either could the calls be rejected or he didn't hear them, or probably he was asleep. It's kind hard to know the real reason.
4: I have heard about [someone] drinking while on duty. He wasn't on duty in the ward but was in charge of transferring a patient or something. He had been drinking at that time with some smell of alcohol coming off him (ha ha). On the way from Taipei to Linkou, he had worn an N95 mask to hide the smell of alcohol and had some difficulty in breathing.

Interviewer: This is something you have heard? Having the smell of alcohol.. wouldn't that affect what the patient thought about the doctor?
4: The patient's family saw [him] with a slightly red face and having some difficulty in breathing while transferring the patient.
(Everyone laughs out loud)
3: Appreciated.
4: [He] might be thought as extremely tired and he must have looked very tired or like he was sick but still helped out to transfer their sick family to another hospital, that must be how they[the patient's family] felt!
Interviewer: Did you hear this from the patient's family?
4: The person who had alcohol told us about it.
3: The patient's family must have thanked him and this is from what you can guess how the patient felt.
Interviewer: So that happened [while he was] on duty at work.
4: [He] was called at short notice and possibly for helping transfer the patient.
Interviewer: Others of you have heard of or been in this kind of situation?
5: Actually I think if people think it's a serious matter that getting caught with smell of alcohol, they will try to get rid of the smell.
Interviewer: 4 just talked about whether the views about colleagues [will be affected]…? Whether professional image or doctor-patient relationship will be affected? What's your personal view about it?
5: If you can hide it [the smell of alcohol] well, the patient might mistakenly think you are just being tired, unless you are unlucky that they happen to smell the alcohol coming off you, then there is nothing you can say about it if you get caught.
1: It's likely you will get phone calls and it'd be better if you don't drink. But there is definitely no way to avoid it for some people who are on call all the time.
Interviewer: What does the rest of you think about this kind of behavior?
3: I think it[what he did] was impressive.
2: Was that a situation where he was on call everyday? Or he was called at short notice?
4: He wasn't outside either [of the hospital].
2: He was also not on duty.
4: He wasn't on duty in that ward. He was possibly assigned to transfer the patient, he was invited to have some drinks by others.
2: This seems ok. It's totally unacceptable if you still drink alcohol despite you know you are the priority doctor on duty.

Interviewer: What do you think the motive is for work-related drinking behavior? The drinking behavior related to colleagues, seniors or teachers.
5: It helps build up a closer relationship easily and people get along with each other better.
1: It's a way of self-entertainment and getting everyone happy.
Interviewer: [It] helps relax more.

3: I think it's no different from drinking regular beverages. Drinking some alcohol at a dinner gathering can be possibly thought as some junior or senior high students drinking coffee to boost up their energy. [The way] we drink to relax is pretty much the same as drinking some other beverages. I know we might get drunk but we won't make ourselves drink till wasted. In this case, [alcohol] it's considered just a [regular]drink.
1: Let's say that's what some of the seniors used to do before. We always have alcohol to drink for all the gatherings after that.
Interviewer: Is it a tradition?
1: Not really a tradition. It's not bad for an entertainment. It's thought better to have some alcohol to drink [for all the later gatherings] so it's easy for people to talk to each other, this is better.
Interviewer: You think the meaning behind this is entertainment.
1: Yup. It's not as serious as the social drinking in business industry. [What we do] is to drink a little and have some chat.
2: Same as what 5 said, by making a toast, you get to know someone better and there's a better interaction. Making a toast to everyone from the group of overseas visitors at a gathering helps a better interaction and get to know each other well.
Interviewer: Making a toast? Were the teachers or seniors from that group asked to do so?
2: I personally haven't been to any event like that.
Interviewer: Are the visitors urged to drink?
2: I haven't met anything like so, probably because we are too young, peers.
1: [They] will ask you to stop drinking if they know you can't drink anymore.
4: We're more rational.
2: Those from the division of general surgery can hold their liquor well. The R1 and R2 just had their welcome parties, didn't they? It's more likely they would have such experience. [This is something to do with] the reliability of a population sample. We're still too young.

Interviewer: Will you arrange a drinking event if you are to hold a dinner gathering?
2: I will.
3: It depends on the theme for that gathering. There will be alcohol provided if there are participants who can drink but there won't be any for a simple gathering. There will also be [alcohol] provided if going to a restaurant like the one serving fried dishes.
Interviewer: What do you mean by simple?
3: [a gathering of] 2-3 people, at short notice.
Interviewer: What about the rest of you, would you hold a dinner gathering like the one at the division?
3: It's optional, it's not like everyone has to drink.
Interviewer: What if you hold your own drinking activity?
2: I will[provide alcohol]. It's [the event] only an entertainment. You are free instead of being forced to join and there is no pressure. It's your choice to drink or drink with someone till you drop. [It's] a fun atmosphere. It depends on the theme [of the event].

Interviewer: Do you mean there is a lot of themes?
2: It depends on the purpose [for drinking]. Someone can get to talk to someone whom they've never known by drinking. It's unlikely to drink particularly at gatherings on a daily basis. You may drink for celebrating anything special. It depends on the occasion and purpose.
Interviewer: Do others of you possibly have different views about this?
2: I don't think drinking is considered bad, I think it's ok if drinking in private. Taking it as a serious issue doing harm to professionalism is something that will only happen if you drink at work and that's definitely going to cause harm. It's also considered unprofessional and a disrespect to the patients. It's ok as long as the private life is separated from work. This is something similar to, let's say a nephrologist who smokes in private and I am ok with it because this is part of his/her private life.
Interviewer: We just talked about being affected the next day, things like hangover and smell of alcohol.
2: You surely have to know when to stop (drinking). It's not good to have a hangover.
Interviewer: Will you keep doing in the same way if once you become a supervisor?
2: Sure, I will.
Interviewer: Why? What will you do?
3: It [alcohol]provides an option, the colleagues invited by the visiting staff to a dinner gathering will be given an option to drink alcohol. Well, not only alcohol.
1: I think it depends on whom you drink with. It'd better not to drink so often if you a supervisor responsible for students [assigned to you]…What I meant is that…not that… It depends on whether your students are mature enough or something, then it's probably ok [to take them to drink alcohol.] It depends on the person you drink with. It's fine to drink with your colleagues.
Interviewer: You meant you will do the same as what they used to do when you later become a supervisor?
1: Only occasionally.
Interviewer: What about the rest of you?
5: Frankly, it's [what they do]not really officially considered a drinking culture, it's something you do whenever it comes to you, it's not really a drinking culture.
Interviewer: What do you actually do?
3: What do you mean by that?
Interviewer: What I meant is that will you keep this drinking tradition at a gathering or will you change it?
3: Whether to drink or not depends on whether the people who join the dinner gathering want to drink or not. They can drink if they want to and they won't be forced to drink if they don't want to.
Interviewer: That means you will prepare alcohol.
3: Most likely I will. The alcohol will be there regardless of whether people will drink or not.

2: If drinking brings joy then it’s not a bad thing. The people who cannot hold their liquor well come to the event and end up in a drunken rage. This totally depends on the participants [of the event] and their intentions.

Interviewer: Will the colleagues or teachers ask others to drink while making a toast?

2: No, they won’t. No such thing as being forced to drink.

4: We haven’t developed a habit of drinking. As being a supervisor, [people] are welcome to prepare alcohol for a gathering where it’s acceptable for them to drink as long as they drink moderately. It’s also a way of adding the excitement as when someone feels like drinking.

Interviewer: Drinking behavior adds the excitement.

5: Same as 4, I don’t personally drink but will welcome anyone who has provided alcohol. I don’t specifically prepare [alcohol]. For me, [I] don’t really like its taste. I am ok with others drinking but will never take anyone to a lounge bar on my own initiative. I will only choose such place [to drink] when I promise someone who has suggested a drink the next day, otherwise I won’t choose the place for a gathering.

Interviewer: You personally dislike it [drinking].

5: Instead of hating the drinking habit, I purely don’t like the taste of alcohol. I am not against alcohol in any particular way.

Interviewer: Lastly, anything you want to add? So it is to say you won’t force your subordinates [to drink] when you become a supervisor.

5: The people you chose [here] just don’t happen to favor alcohol in particular.

2: Do the current supervisor ask their subordinates to drink? I think it depends.

Interviewer: It depends.

2: We are all easy-going, you can drink whenever you want and it’s fine if you don’t.

5: We have 2 girls right here. On an occasion where there are girls, they are less likely to be forced to drink or anything.

4: I have got something to add, the possible reason for drinking, such as the teacher taking us to a wine-tasting [event], one of the possible reasons, for example, in a paper, it tells you how to do wine-tasting, teaches you how to drink wine, the ways of drinking red wine or what food to go along with which wine and how to describe the taste of wine, this is like to get a deep insight into the drinking culture. The only difference is that alcohol is really provided [at those events].

Interviewer: You’re saying the teacher will guide you to understand the drinking culture, is that it?

4: Wine-tasting. Getting to know what food goes with what type of wine, red, white or sweet wine; the way of holding the drinking glass; the way of drinking or what food to go with the flavor of which wine.

Interviewer: So all the teachers will always teach students like you.

4: Not all but some. They will take you to experience wine tasting if you didn’t use to have such opportunity to experience it before.

Group 8

Physicians (n=4)

Group 8

Interviewer: Please introduce yourself and tell us your interests and the type of food you like.

1: [I am from the] Division of pediatric, neonatology. I like desserts and watching movies.

2: [I work at the] division of emergency. My interests are gourmet food and movies. I don’t drink often but only drink beer occasionally.

4: Movies and gourmet food. I don’t drink at all but sometimes drink at divisional gatherings. I can’t hold my liquor well.

3: Pediatric division. I also like dessert the junk food. I actually don’t drink or drink on my own initiative. I only drink a little for socializing but sometimes I will get urged to drink by [my] boss. My face turns red easily even it’s only a little alcohol.

2: The people you have here for the interview don’t happen to be the ones who can hold their liquor well. Is it on purpose or..

3: Not really, you are one of them too.

2: Am I? No, I can’t hold my liquor well.

Interviewer: It’s not on purpose. Of course, it’d be better if there’s more with experience of drinking. Anyway, it seems everyone likes dessert, do you drink alcohol with it? Do you have the habit of drinking?

1: For me, I don’t have a habit of drinking nor really like drinking alcohol. I hardly drink at all at divisional gatherings. If asking about how well I can hold my liquor, I never had been drunk or got drunk to the point of throwing up. I don’t actually drink that much.

3: How many bottles?

1: [I can] finish a half bottle of Shaoxing in one meal.

3: A half bottle of Shaoxing is supposedly considered a lot.

1: That’s my only record for drinking the most.

Interviewer: Where was that?

1: At a family’s wedding banquet.

Interviewer: Have others of you had anything like it?

2: No.

3: Neither have I. It’s mainly because not many people around me like to drink, another thing is that I don’t find [alcohol] tasty. If I think it’s tasty I will keep drinking it. It’s fine to drink it occasionally but most of the time I don’t really like it. Like the Japanese sake, I actually find it alright [to drink] but it’s still not as tasty as if I need to drink it whenever I am free.

Interviewer: Where do you drink mostly?

3: During meals.

Interviewer: While being at a restaurant.

3: Yes, at a restaurant where there is alcohol provided. You can get to drink some when someone else beside you orders [alcohol], and they will say it’s not ok if you refuse to drink.

Interviewer: How often do you drink?

2: It depends on how often I go to a restaurant.

3: Probably 2 months. It’s common to drink with Dr. Yeh the associate chief physician. I haven’t had dinner together with Dr. Yeh for about 2-3 months.

Interviewer: About 2-3 months and at a restaurant.

3: I won’t drink if I go by myself. We order drinks if eating with colleagues and this is ok, the only thing is that most of the seniors will prepare alcohol for you if it’s, let’s say, a little formal occasion.

Interviewer: Could you please share work-related drinking experience?

1: It’s only twice a year. Let’s say they [the sales people] try to introduce a product and it’s a good opportunity for taking us to a restaurant and ordering alcohol, but generally we won’t drink too much because mostly there is red wine provided and [we] only take one glass [of it].

Interviewer: [Is this] at a restaurant with colleagues?

1: Yes, the occasion where you will get to meet the supervisors and drink the most is mostly an event like the year-end party, it’s not likely to drink a lot at other events.

2: The year-end party, sometimes you have to drive to get there, it’s mostly held at a place where you have to drive to get there.

1: From my own experience, it’s more likely to get urged to drink at events like the year-end parties. For those research conferences held by [pharmaceutical] companies or [medical] societies, it’s likely we will drink in the evening at the conference place where we get to stay for 2 days and 1 night, but we will not get drunk, yeah.

Interviewer: Could you tell me more about the occasion where you get to drink the most at a year-end party?

1: There is [people]toasting, people of the same subspecialty sit at one table, halfway through the meal, the head of each subspecialty would take everyone to walk around, everyone then has to drink and drink. You have to drink when they come to your table.

Interviewer: All the supervisors?

3: 1 what you meant is something like this table is for Chang Gung Hospital and the other table is for Veterans General Hospital, it’s assumed that’s how it is arranged, this is what I heard from him, what I possibly think he said, or all the people of this division would go to some other table to make a toast or things like this.

1: Doctors from the pediatric division also do the same. Say the doctors from the division of allergy take all their doctors to [the table] where the doctors from the gastroenterology [sit]. This is probably what it is like.

3: In that case, you won’t have to drink much unless you have met someone who insists you to finish your drink. For events like this, making a toast is always something done out of courtesy, there is nothing like someone taking the whole bottle with them for making a toast! (Everyone laughs)

Interviewer: So how do you make a toast to each other, you drink a little?

1: Yes, that’s right. You take a sip without finishing the whole glass unless there is [someone] special, say the director from the teaching department who has invited the staff of the teaching department to the dinner gathering. It’s more likely to drink more at an occasion where there are supervisors and subordinates.

2: Not to mention that the supervisors also drink.
1: That depends on whether the supervisors will drink or not.
3: Like Dr. Yeh, he pours you a drink and waits for you after he finishes his drink.
2: [He] prepares his own alcohol.
Interviewer: Is that his supervisor?
1&3: It is.
1: For me, I don’t really drink either at work nor when I am with my colleagues, hardly any.
Interviewer: What about the rest of you? Any work-related drinking experience?
4: Similar to what 1 said, a divisional gathering that is. The year-end party is about once a year. The last time I remember when I had a drink was probably the time when our chief physician retired.
2: The chief physician can hold his liquor well.
3: Ain’t you the same too? (ha ha ha)
4. I don’t drink most of the time. My face turns red and I start to feel dizzy once I drink, and I only take a sip instead of drinking it up at once.
Interviewer: What was the atmosphere like?
4: They were hyper at that time.
1: It’s like waving the flag and shouting….
4: We were like the standby assistants taking care of chores and…because there were usually certain people who would drink with him [the chief physician], we wouldn’t be his drinking companions.
Interviewer: Who were those people?
4: The supervisors from our division.
3: There is a different way of drinking for every division.
4: [I] don’t really drink.
3: Now.. At the time when we were in Xiaomen, after the accreditation of Chang Gang in Xiaomen, there was the dinner gathering where everyone was asked to drink up from a glass of this huge size, everyone had to drink up and no one would dare refuse.
4: Is it impossible to refuse?
3: You would be the only one left without drinking as everyone else had drunk up. They could really hold their liquor well and they didn’t drink less than you did, those were the supervisors [I am talking about].
Interviewer: They were all supervisors?
3: There were supervisors sitting there and they also had to drink.
Interviewer: How did you make a toast?
3: One glass to another.
2: It’s a ritual.
3: Whether [you] have shown your respect or not. Unlike doctors, it’s more likely for those from the business industry to drink for socializing in a terrible way.
3: Say for some research conferences which are considered academic, where we also have to make a toast, those [other than doctors] are often excited to get to drink with doctors and we don’t necessarily have to drink a lot. They think that’s what they show to have every intention of drinking with us by drinking

it up but they won't force us. But for them, you have to drink 3 glasses for being late.

4: To drink up to show respect.

2: There are also some other people who are really into drinking and wine-tasting. I want to mention my wife who works at the surgery division. She and 4 to 5 visiting staff from Koo Foundation Sun Yat-Sen Cancer Center will have a dinner gathering about once every one or two months. They will take their partners and go to a nice restaurant. The chief physicians and seniors will take bottles of wine from their collection at home, [from]a thermostatic chamber of a variety of red wine, white wine or sake. They will take 2-3 bottles on their own and they like to drink. Every time at the gathering, you can hear them saying [what] wine goes with what meat, all the wines taste the same to me but they think it's a way of having a taste of life for them.

3: Saying they can't drink means totally different from saying we can't drink that much.

(2 laughs)

2: I drink occasionally at a gathering like that and it's about once every week or every 2 weeks. I would buy a bottle of beer and Ko-Lo-Ko[snacks] when I watch MOD [TV] with my wife at night.

Interviewer: It is to drink once a week, is it?

2: Once every week or two weeks. [I buy]Beer or root beer sometimes.

1: It's not common for us to drink at home. We have 2 bottles of sake sitting at home for a year and no one would drink it.

2: [I am] not likely to drink strong alcohol at home.

3: But the time I had dinner at his(1's) place, he did take out the red wine, I find it weird.

1: My family and I don't drink. I also didn't drink the one given by the pharmaceutical company to me as a gift, so I offered it to guests at my place when there is enough people for helping me finishing the wine. There is always wine left in that bottle after each meal then I have to gradually finish the remaining half bottle of wine by myself. I don't really like red wine nor fruit wine. I like sake more, [which is]rice wine, sake is made from rice.

4: [Sake has a]A higher alcohol content.

1: I don't like what I get after drinking the fruit wine.

3: There is this ice wine which is not bad.

1: There was no other time except for the time when XX invited me was the only time I had drunk.

4: I had tasted ice wine in Canada, it's sweet. It's cold there and the frozen grapes are used for brewing. Every winter, there is something like a fair selling agricultural products, along with a lot of other stuff. We checked it out and tried the [wine there] out of curiosity.

3: I have to say that my daughter loves drinking alcohol.

2: Hey… [she is] underage.

3: Second year of senior high. Few days ago, last week, I even bought her a bottle of beer. I grabbed a pineapple beer from the supermarket. [The alcohol content] was 3 or 2.5 %, I forgot. Others are not allowed [for her to drink].

2: Sometimes the wine chicken soup is more likely to have a higher alcohol content.

Interviewer: 2, you just mentioned about preparing your own alcohol to a dinner gathering, didn't you?

2: 2 or 3 seniors from their division would sneak in with their own alcohol.

Interviewer: Was it a gathering with colleagues?

2: Colleagues whom they know quite well. There are 4 to 5 visiting staff at their division and they have a dinner gathering every 2 or 3 months. They all know each other well.

1: It is meant to say what division you are from makes a difference.

2: Those from the surgery division are more likely [to drink], it's probably because doctors of the surgery division are generally considered to be those who enjoy drinking and can hold their liquor well.

Interviewer: Have you ever been to a year-end party?

2: Did you mean their year-end parties or what?

Interviewer: Yours.

2: Our emergency department, there are a lot of people who like to drink from the emergency department of other hospital I used to work at, but the supervisor from my current emergency department doesn't like to drink. There is always alcohol provided but many people drink juice instead of alcohol because they have to drive.

Interviewer: So how did you make a toast to each other? You also drank a little?

2: It's ok to drink juice, there is also alcohol for whoever wants it. Making a toast to someone with juice is as fine as alcohol. I think there is only pressure from some supervisor at some occasion where it makes you feel like you have to [drink], or when you don't know the participants well. Like the doctors from our division, we all know each other very well. It's ok to make a toast with juice to someone even like our chief physician.

Interviewer: So the supervisor wouldn't say something… does he urge others to drink?

2: We all know each other quite well. Only if we didn't know each other that well but there is…

3: You should interview someone from ENT next time, the doctors from ENT, I am serious.

2: If you don't think your boss will do anything to you because you don't drink, you think he won't make you feel this way then it should be ok. But some typical Taiwanese bosses think drinking up is a way of showing great capability.

Interviewer: You may talk about your previous experience.

2: I was an intern at the emergency division of Veterans General Hospital at that time. The chief physician I had was from the neurosurgery division and he could really hold his drink well.

Interviewer: Did he ask you to drink or something?

2: There was this atmosphere at that time. It was those of lower ranks or interns who made a toast to visiting staff and the chief physician with a whole glass of something possibly with a higher alcohol content, [there was nothing you could do] but followed the crowd.

1: Did he also have a military background? [Those people are also considered] to be able to hold their liquor well.
2: Possibly.
3: Try asking those from ENT, the division of ear, nose and throat. I don’t know but they used to make their R1 who were new to the department drink. They were forced to drink to the point of losing consciousness and even ended up in having a foley catheter inserted.
Interviewer: At a welcome party?
3: ENT. Yeah, Possibly.
2: At the emergency department, I had met some PGY, from obstetrics and gynecology of Keelung, who drank till they dropped and were sent to the emergency room. They had both fecal and urinary incontinence. They would remove [the catheters] and left without anyone noticing the next morning. It happened for every one or two months, [a doctor] is sent to the emergency room by their colleague and ends up in having an intravenous drip till the next morning.
Interviewer: 1had just talked about being forced to drink, where did this happen?
1: When you are invited to a dinner by your supervisors, the supervisors will do that.
Interviewer: Did that also count as a divisional gathering?
1: Like 2 said, it still depends on whether your supervisor likes to drink or not. During a divisional gathering, if your bosses don’t like to drink, there is probably no such atmosphere that makes you feel like drinking.
Interviewer: What if the supervisors do like to drink?
1: Dr. Lin the superintendent is probably the only one who will drink from the pediatric division, others are often quite shy.
3: But he doesn’t really force you to drink that often, does he?
1: Of course, he won’t.
3: [They] think some people can drink. Like his boss, the doctor, Mao-Sun Liu who can hold his liquor well but he probably won’t force others to drink. There are some who can drink a little but still unable to hold their drink well; there are those who don’t even drink much but keep forcing others to drink, it’s like kicking someone when they’re down, or people just feel happy in general when there are others drinking.
Interviewer: You have seen things like this?
Interviewer: Is it pretty common? A divisional gathering?
3: Yes.
1: I had experienced twice and nothing else, the only two times when Dr. Yeh, the associate chief physician responsible for teaching, was involved.
3: Not really. Guo-wei Yeh and Ging-Ron Huang will do it too.
1: It’s like so for your pediatric division. I haven’t experienced anything like that but the only two times when Dr. Yeh, the associate chief physician was newly assigned to the teaching department.
4: Did you say they can’t really drink but will force you to drink instead?

3: It’s just that we find someone whom will be urged to drink by everyone, everyone will try to tease this person and make a toast to him. This person probably drinks less than you do or he does.

Interviewer: Is it a subordinate or supervisor?

3: A supervisor for sure!

Interviewer: It’s the supervisor who will ask the subordinates [to drink]?

3: No. Let’s say I am your supervisor and all I do is to keep making a toast to you, get it?

2: I think sometimes there are people who really like to drink. Take my wife as an example, her chief physician is a person who enjoys drinking and prepares alcohol [for events]. I can’t drink much and have to drive so I don’t drink, but she can drink more. There are also some people who happen to enjoy the [drinking]atmosphere where alcohol or urging others to drink is a must. They can’t help it in that atmosphere they enjoy.

Interviewer: We seemed to just talk about a [drinking]behavior that would only be seen in a relationship between the supervisor and subordinate.

3: Or it could be between colleagues. Well, probably you can’t put it that way. Let’s say our chief physician, Guo-wei Yeh, who won’t definitely help me out if we meet [at a drinking event]. He definitely will say something like “Come on, just drink and drink! You already made a toast to the superintendent!” But I think I would also probably do what he did, if I was aiming for a position like his. (laughs)

Interviewer: You meant you would also force them to drink?

3: We all felt it was fine when I didn’t drink but he did.

Interviewer: Will doctors ask their subordinates to drink more?

3: We won’t do anything as bad as what you just said. It’s only that sometimes you feel awkward. It’s not as far-fetched as something like you just drink as told, or like things I heard before about drinking 3 or even several glasses [of alcohol] in response to one, making a toast to one after another, no, nothing like these would happen. I think it has got to do with the culture more or less. It means there is no excuse for you once your boss had drunk. It’s like something related to culture. Like for some people who don’t like to drink but they will still get forced to drink by their bosses anyway, right? Once they have become the bosses, they will also force their subordinates to drink. They won’t really force you to drink a lot. This is like they want you to have one or 2 glasses instead of getting drunk. The least you can do is [to drink] for showing your respect to them, you should at least show as you have every intention of doing it, I think this is what it’s all about.

2: Like 3 said, alcohol itself is not the point any more, if you change it to bitter tea or anything that you can’t drink too much at once, it doesn’t matter what you drink as long as everyone can get excited, it’s just that you can’t drink most of the alcohol too much at once, [alcohol] is only for adding the excitement.

Interviewer: Has any of you been in that kind of situation before, like being forced to drink by the supervisor, anything like this before?

3: Sure, there were always people who forced others to drink but I would try my best to hang in there.

Interviewer: Does that mean you drank as well?

3: I will try to avoid drinking it until I can't. Say other chief physician or superintendent makes a toast to me, I will only drink a little even if they drink up. If the boss has got something to say then yes, it's a shame on us, what you gonna do about it? We are more disobedient, that's right. We shouldn't aim high without considering our abilities. We would try everything to avoid drinking.

1: Basically, you are not so into drinking either.

Interviewer: It's to say that you won't make yourself to be forced to drink.

3: But it depends. The occasion we just talked about was when everyone knew each other well. Sometimes an occasion like that is more formal, where there are still people whom you don't know that well and in this case you might have to consider taking the alcohol while you can. So it depends.

Interviewer: Why do you want to drink?

3: It's actually kind like being forced instead of feeling like drinking it. You feel you are forced [to drink]. Saying no [to drink or not] in this case seems to be a serious…

4: [a serious thing related to] manners.

3: [you] Don't want to be rude.

1: Just drink it out of courtesy.

Interviewer: What kind of formal occasion is this?

3: This is pretty hard to say, whether it's formal or not.

2: What is your relation to those who sit at the same table as you do?

Interviewer: Others of you have been in this kind of situation?

3: She/he doesn't seem to drink when you asked 4. On which occasion will you drink up?

4: I never drink up.

3: It means you are able to avoid [drinking] no matter how others keep forcing you to drink, right? Let's say Dr. Yeh and everyone else altogether, 5 of them all drink up except for you.

4: [At that time,] I took a sip of the wine I took with me and that's when I started to feel dizzy, and they would also see me looking weird. (ha ha)

4: Asking me whether I am fine or not and of course they didn't keep asking me to drink. For me, I personally knew it was bad for me because I really didn't feel well after drinking. So I won't force myself to drink. Sometimes I will taste the alcohol, which I know to be with a good quality and provided by them. Like Dr. Liu the chief physician, he/shes prepares some very good wine and explains what flavor it is for which wine, fruity smell or something. There are 5 levels in wine-tasting.

3: Smoked flavor.

Interviewer: [Was it with] colleagues?

4: The chief physician, of course. He has that kind of taste and special skill so that he sometimes wants to find out what sort of alcohol we like to drink. He won't tell me to drink up nor force everyone to drink up. It's only because he

personally enjoys drinking and usually there is his drinking companion, XX…. (ha ha)

1: Dr. Liu is considered retired. Next year, it'll be Dr. Yeh the associate chief physician, either this year or next year.

Interviewer: 2, have you ever been to a year-end party or a divisional gathering?

2: I have been to a year-end party. Those from my division don't really drink. Once at our year-end party, a senior, Dr. Chen who knows much about wine was asked to provide some wine for everyone to try since our chief physician didn't attend that year-end party at that time. There was indeed a lot of wine prepared and lots of good wine were provided at the year-end party of that year. There is always a fixed budget for a year-end party. If you spend the money on alcohol it means you will have less budget for the presents. [At the end], Everyone blamed [Dr. Chen] because they'd rather spend the money on [presents for the] prize draw. Our year-end party seems ok, where some red wine but not of very good quality will be provided, and no one will force others to drink. I think most of the people there will either drink tea or juice; the point is not about the alcohol any more.

Interviewer: Do the supervisors drink a toast to each other?

2: The ones we have currently don't. Few from the middle generation like to drink. Those who like to drink will sit together and more wine will be provided at that table too.

Interviewer: You won't force each other to drink because you all know each other well.

2: No, definitely no. It's fine because you meet them every day and get to know who loves to drink and who doesn't. People will take their kids to year-end parties or some will drive there.

Interviewer: When you drink with supervisors, teachers or colleagues, do you think drinking at the gathering will affect the way you see them normally, say drinking with your teachers or supervisors who normally look serious at work, will drinking at an event have any effect?

4: You meant whether knowing your supervisor who drinks at a gathering will affect the way you see them normally at work, is it?

Interviewer: Say whether what you have seen from someone drinking at a dinner gathering will affect the way you see them if you know how they always look serious at work?

3: Did you mean the impression of this supervisor …but is it going to be the first time to have a drink with them and whether this would change anything afterwards?

2: I think there will be no change unless [they] have gone too far with drinking. Something way out of line like getting too drunk or throwing up.

3: Unless they always turn nasty after drinking.

1: Last time, the time we went to a Japanese restaurant with Dr.Yeh and Dr. Liu the chief physician, Dr. Yeh was too drunk to stand up from the dining bar. We sat on the tatami the Japanese mat with a hole of space to put our feet in and he wasn't able to get up. If it wasn't for Dr.Hsieh and I who lifted him he couldn't stand up at all. We carried him to Dr. Hsieh's car. When they got

into the car, Dr. Hsieh asked Dr. Yeh where he should drive and where his place was, Dr. Yeh replied that his place was nearby and close enough to walk, there was no need to drive. Dr. Liu fell asleep on the chair after taking a piss. They had really gone too far with drinking, but it was ok, it was ok for us.

3: [Feeling] it was fun.

1: It didn't change our views about them or anything [if what they behaved was] different from that at work.

2: Drinking doesn't affect work.

Interviewer: What's your personal view about them?

1: It should be ok because we know how to make a clear separation, don't we? We don't drink at work.

3: If they drink during work our views about them might be affected. We won't have any problem if they don't drink during work and they can show up for work in good shape. We will just know one of their interests is drinking.

1: It should be ok for a girl. We haven't seen anything out of line or improper talk [from girls] so it's not for us to comment.

3: Have you done anything out of line?

1: No. I never really drink too much.

Interviewer: Any unforgettable events you have seen or experienced?

1: That about Dr.Yeh and Dr. Liu we just talked about, when we were about to leave [the restaurant], Dr.Hsieh asked them if they needed us to accompany them to walk. Limping and swaying from side to side, they walked off while saying "No, there is no need" to Dr. Hsieh, but we felt terrible seeing them like this. (Says this & laughs) We were like thinking it was unbelievable they got themselves too drunk to even remember where they lived. They were used to drink like that though.

Interviewer: So this doesn't affect the way you see about them.

1: Never. Dr. Yeh no longer invites us to have a drink any more. I think the biggest reason is because we don't drink.

3: It's no fun to drink with us, or it is a waste of money in some way. The wine he has is very expensive and we can't tell its worth by drinking it.

1: [He] doesn't come to us anymore for having a drink or meal with us. There is fun in drinking but we can't drink.

Interviewer: Have others of you seen or heard anything like the situation where their views about their teachers or colleagues are affected?

3: I think there won't be a direct [influence] but I think I won't feel good about it. What I meant is that if this person really can't drink, say I am the one who can't drink, if I know someone who is being forced to drink, I won't do anything like kicking them when they are down, this is not what I will do. But if someone who can really drink, I will probably do something as kicking them when they are down and use the same way [of forcing them to drink] to make them feel what it is like. However, I think it[drinking] is not a good habit at all in some way. This is hard to explain, but personally I think it's not an acceptable behavior, if you force others to drink and still keep trying to make them drink despite they have turned you down politely. This is how I feel. About what you just asked, someone doesn't assume others to be in the

same way as them even if they can drink and say they can hold their liquor well, in some way I think this person is considered a nice fellow because they won’t make those who can’t drink uncomfortable.

Interviewer: Some of you talked about East and West, there is a cultural difference. Despite westerners love to drink they don’t have such culture of forcing others to drink. This is the difference between East and West.

3: They drink beer all the time, don’t they? They drink beer as if it’s water.

4: They drink with every meal.

Interviewer: A cultural difference, they don’t force others to drink like Easterners do.

3: I remember Lynn can drink and hold her liquor well. See if all of you together can outdrink her, you’d better see for yourself and give it a try one day!

Interviewer: How did you know?

2: She likes whiskey.

3: The time they had strong alcohol to drink and it was prepared by Dr. Yeh.

2: She drank half of the bottle.

3: She took it home and she probably wouldn’t force me to drink. They drank on their own.

Interviewer: Westerners are not like that [forcing others to drink]. Has any of you had the experience of work-related drinking affecting your work normally?

3: [I]Don’t really get what you meant by that. Whether drinking at a gathering affects work or not, what was your question?

Interviewer: Say having a drink at a gathering, will it affect medical practice the next day?

4: Those whom I know, the chief physician and Dr. Yeh, I don’t think I feel anything different about them the next day

3: There might be someone who is like that but it’s not easy to find someone like that among us at such place where we are.

4: If it’s for someone who has a habit of drinking, I don’t know if he/she is personally affected.

3: Or there might be an effect but we just don’t see it.

2: Well, first you have to know this person has had too much to drink the night before and next day you happen to work on the same day as he does, so you get the opportunity to observe him closely, but chances like this are rare.

Interviewer: You have never heard any[anything like it], say the dinner gathering after work.

3: It’s more likely to be seen overseas, binge drinking until you work is affected, this is considered out of line. I wouldn’t call it drinking but alcohol poisoning instead or …well, I have no idea.

1: We haven’t met any among us and I think it’s because we don’t like to drink.

3: You should interview Dr. Liu the chief physician and Dr. Liu so that you can get different points of view. They are artists, artists who drink. Or they are like De-lung Wu, you know what that is? The highest consultant for the wine-tasting society of red wine, he is a master of the highest rank and has a lot of students. The deputy superintendent is also his student and that’s how you look at it.

Interviewer: It’s likely to be forced to drink because of the culture and this might have an effect on work or professional image.

3: We should put it this way, here we don’t have much of the situation where others are badly forced into drinking. Basically, there is always this custom of making a toast at social events but we don’t know much about the definition of being forced to drink.
Interviewer: It’s like asking others to drink.
3: The point of forcing others to drink is to make them drunk, is it?
2: This is probably mostly seen at weddings nowadays but not at other events.
3: What is mostly seen?
2: To see people forcing others to drink, that is.
3: How do they do it?
2: It’s like you must drink as told and they will say, “I’ll pour you more drink. Drink more, drink! You are the bridegroom!”.
3: Did you get forced to drink?
2: A little but it was ok. (laughs)
4: I think at most of the weddings or dinner gatherings, they won’t force you to drink if you have made it clear that you don’t drink. You won’t feel like being threatened because it’s only an occasion where you take a sip or drink a glass to make a toast out of courtesy, and something like this. In this case, it doesn’t really count as being threatened.
Interviewer: About the drinking behavior we just talked about, what do you think the motive is? Is it done out of courtesy?
4: I think for an occasion like that you do it out of politeness. Say my chief physician was about to get retired and he invited us to dinner, everyone was happy at that time. He prepared good wine hoping to share it with us. We would take a sip or two out of politeness. I think even if I didn’t drink he wouldn’t feel that I was being rude.
3: What she [the interviewer] meant is that why do these people make a toast or force others to drink.
2: I think this is a tradition at our society in Taiwan. We always have to drink up whenever there is alcohol left in our cups between two of us. It’s a way of showing your sincerity and respect. A custom naturally arose because of this.
3: A custom that wasn’t made but arose. The person may not know how to show their respect and drinking helps him….
Interviewer: As a way of showing respect.
2: Gratitude and respect.
3: But then the custom gets corrupted.
2: Yup, it does and it should be worse in the central and south parts [of Taiwan].
4: I think if we are not talking about the medical field, drinking is necessary for some work where people do jobs related to sales. It’s probably nothing serious about drinking for doctors like us.
1: Our subject is about medical personnel, right?
Interviewer: Yup, doctors.
1: Actually, not everyone from our generation drinks like that, nor those from the older generation like that of Dr. Yeh’s.
2: I think the prevalence of drinking has declined for both generations.

3: If you go and ask some senior and experienced doctors, you might get different answers or thoughts.

2: I think those surgeons in their fifties or sixties of that generation, [they drink] probably to reduce stress.

Interviewer: We will focus on this generation. 1, What do you think? What's the motive of this drinking behavior?

1: Perhaps it's about being polite. I think someone who takes it as being polite is also a person who likes to drink.

3: I think it has nothing to do with being polite. It's a custom but it's changing to be a better one gradually. Things like from showing a strip dance at a wedding banquet to offering you a cigarette or the betel nut, are something what a person does to show their hospitality and generosity, a way to show they have every intention of inviting you [to an event or do something].

1: There is still strip dance to be seen at some wedding banquets.

3: It will get less and less likely to see that. It's also not that easy for this kind of thing to be gone forever. We haven't found a better alternative way to show our hospitality. It's likely that Easterners are more introverted but it's quite often they will try to show their generosity at events like those by doing things like what was mentioned earlier.

Interviewer: A way of showing your hospitality.

2: There is this group of people who enjoy wine-tasting. They are really alcohol lovers but they don't do binge drinking. They like to taste [the alcohol]. Like the brewing or wine-tasting culture which has been maintained for over thousands of years, people of all sorts of different groups are all doing this wine tasting all over the world, there has got to be something deeper behind [this culture], something you can get an insight into or have as your own taste.

3: What 2 said is something similar to saying someone who likes drinking coffee.

2: It's also like what we talked about at the start, the case about my wife and those from her division, or something about the way you enjoy having gourmet food.

3: Enjoy listening to music or drinking coffee.

2: It's like art, something wonderful in life that you try to get an insight into or develop it as your own taste.

3: At that time, Dr. Yeh accidentally bought a bottle that cost 900 grand or more.

2: Think about it, it's a huge market for a lot of people to spend a lot of money, it is impossible to buy a Coke for 100 grand or something.

4: I think it [drinking] also has possibly got something to do with your family background.

3: Usually if the parents drink, their kids will too.

4: Take it as a subject to study. As a kid, parents drank some red wine when they were in a good mood or they would take us to visit a winery.

3: It's likely that what he said is very important. It's likely that the culture leads to this [drinking behavior]. In ancient times, it was not easy to have alcohol all the time. It felt like a reward to have alcohol or things like such provided at special events or celebrations. It was also a way of building up a closer relationship and similar to something like offering a giant male pig [for

slaughter] or something else. But [this custom] has gone bad as economies grow.
4: If you drink red wine like the way you drink water, [probably your senses of] taste and smell are gone.
2: Wine and beer are two different things. You taste wine with every intention.

Interviewer: It sounds like most of you have studied and have some understanding of wine. Will you provide alcohol if you have to hold a dinner gathering or any similar activity?
4: An event of wine-tasting for introduction to red wine was held at the division of internal medicine once.
3: The question was about whether we will prepare alcohol if we have to hold an event, right?
Interviewer: Yes, whether there will be any drinking activity arranged at a dinner gathering held [by you].
3: Arranging a drinking activity or preparing alcohol? They are different.
Interviewer: Arrange drinking events I shall say, like for year-end parties or dinner gatherings.
3: Alcohol as an additional thing [provided] or…?
4: Or we shall say the alcohol there is the same as other beverages.
3: Are we talking about preparing alcohol for those who want to drink or you were actually talking the activities?
2: Alcohol will be the focus if it's for a drinking event.
2: Year-end parties are just year-end parties where alcohol provided is only considered one of the many others kinds of beverages there.
3: There is probably some courtesy you have to consider. You feel like drinking when there is a bottle of wine on this table.
2: There are also tea and juice.
4: Usually wine is provided at events like that and you won't care whether it's tasty or not.
Interviewer: Is it to say that you will prepare alcohol?
3: Yes, it's a courtesy to prepare alcohol regardless of whether there is anyone drinking.
4: It's like when my chief physician invited people to his retirement dinner where he prepared alcohol to share with everyone, and so did Dr. Yeh, they prepared alcohol for everyone because they both liked to drink.
Interviewer: So you will prepare alcohol and will you keep it this way when you have become a supervisor? Or will you change?
2: Preparing alcohol?
Interviewer: Yeah.
4: Those who like [to drink] will and those who don't, won't.
2: Alcohol should be prepared but there should also be other beverages for those who don't drink.
3: We don't seem to understand what you asked. Let's put it this way, will you prepare alcohol for our table at your wedding, you possibly will, right? It seems a little weird if you won't.

Interviewer: It’s work-related gathering we are talking about.

3: For work-related dinner gatherings or social events I will provide [alcohol] as one of the options [for drinks]. It will be available for anyone who wants to drink but I won’t intentionally prepare a lot for everyone to drink. If I am to hold the event I will do it [prepare alcohol] out of courtesy despite I personally hate drinking very much. I don’t do it for myself, for forcing others to drink, nor for carrying on some sort of [drinking] culture. Say there are 6 of us for a dinner gathering, if I know it well that, I totally know that all of us don’t drink then we won’t prepare any [alcohol]. We don’t prepare [alcohol] without any specific intention. This is what I think.

1: It all depends on whether you actually like to drink or not.

2: This is not a very important [factor] for decision [on preparing alcohol]. It’s not a key [factor]for deciding [whether to prepare alcohol or not].

Interviewer: What do you actually mean?

3: Say you like to go to a karaoke bar then we will take you there; You like stinky tofu then we will go and eat stinky tofu together; You like music then we will go to a concert or performance. Basically, it’s doing something which everyone is comfortable with. If you like badminton then badminton is what we will play.

4: Sometimes it’s that the alcohol is always provided there at the gathering place and not prepared intentionally, this is somewhat different.

Interviewer: Does this means that it also depends on whom you drink with?

4: No. I was saying that a place we have picked for dinner gathering which could be a restaurant where alcohol is or isn’t provided. Those who want to drink will prepare their own alcohol if they find out that there is no alcohol provided at that restaurant. Those who don’t drink won’t order alcohol even if it’s available at the restaurant.

4: Like our chief physician who likes to drink…drink wine of very good quality.

3: [I] surely will prepare alcohol!

4: If he [the chief physician]holds a divisional gathering making a special invitation, [he] is definitely going to prepare alcohol, which is something he likes. I think no alcohol will be prepared at a gathering held by another chief physician who doesn’t like to drink.

Interviewer: It depends on whether your supervisor likes to drink or not.

4: If this is what the current chief physician does, it doesn’t necessarily mean the next [chief physician] has to continue doing so, it’s not a must, no.

3: No.

Interviewer: Lastly, anything you want to add or anything you feel like missing?

1: We are the group of people who don’t really drink. Perhaps you would want to do another interview with those who can drink.

Interviewer: It’s not that we only interview those who can drink.

2: This [drinking situation] is not commonly seen nowadays in the medical field. There are few and few of those [doctors]who drink unlike several years ago.

3: Let’s put it this way, drinking is really not the way for you to prove anything at current society. If you don’t need to prove anything then you won’t have to

specifically drink for this particular reason. Now it’s more like you can drink whenever you feel like to.

1: Drinking was always [a way]for proving something in XX’s generation.

3: Yup. Few decades ago, this [drinking situation] was commonly seen in hospitals administered by medical affairs bureau. At that time, some interns of veterans general hospital, who were not much older than me, had to make a toast to their chief physicians at year-end parties under the influence of that drinking culture. They had to drink even if they couldn’t hold their liquor well. In that case, it was inevitable to drink considering it would be more likely for them to be the residents the following year by accepting the drinking offer. This is the same case as in if you are serving in the military. Will you not drink if drinking is the only way that determines whether you will become a company commander the next day. That’s what’s happening and it is really how it was like despite it was few decades ago. This senior of mine [I just talked about] couldn’t drink at all but he still had to drink up no matter what.

Interviewer: Was it two decades ago at work?

3: Say he never drank…..then when he did…..

Interviewer: [Was he] asked by the seniors to drink?

3: No, it was the chief physician.

2: There was more influence from the supervisors.

3: It was like to please the supervisor without making the situation awkward, or else you would be the one to blame.

Interviewer: Buttering up the supervisor.

3: It was like this and more commonly seen before. Things have changed over these years, it’s more like begging the student to come to be the resident now. The chief physician says something like “Come on, let me drink a toast to you, kid” to one of the juniors.

Interviewer: Thank you, everyone. Anything else to add?

Group 9

Physicians (n=4)

Group 9

Interviewer: Let’s get to know each other first. Please introduce yourself.

2: I used to be Ching-Yi’s colleague during internship before.

Interviewer: Any interests or favorite food?

2: I like to read, but not medical related books, mostly comics. The food I like….I am ok with any food that goes with alcohol. I like congee quite a lot these days. It’s like my favorite. Every morning, I look for places that sell congee or anything related.

4: I thought you cook it yourself.

3: Throw in [some of that from] Ai-chi-wei [can of pickled food for congee].

2: It’s not likely for me to cook, there are these side dishes or pickled side dishes to worry about.

4: Buy them and put [them] in fridge. Lots of pickled stuff are very yummy.

2: Congee and the side dishes.

3: I am a physiatrist. Meat and veges are the types of food I like. I like food cooked at home. I like hand-made [food].

4: I am a radiologist. I am not a vegetarian so I am probably ok with everything except for vegetarian dishes.

2: Do you eat veges at all?

4: Sure, I do. I just don’t like anything that’s pure vegetarian.

3: The meat has got to be a chunk of meat.

4: Mince and shredded meat don’t count as meat, it has to be something you can take a big bite of.

Interviewer: It seems like there is a huge difference in what you all eat. Do you drink a little alcohol when you eat? Do you have alcohol to go with your meals?

2: It depends on the type of food I eat. I will definitely order sake if I am going out to a Japanese restaurant with friends, because these days I have my days off. For eating at other restaurants, [I] will order red wine, white wine or sparkling wine.

Interviewer: How often do you drink?

2: Every day. There is always a bottle of wine when eating together with my classmate’s mom at lunch everyday.

Interviewer: Is this at a restaurant?

2: We start[drinking] at noon, [we] also drink a bit at night or in the afternoon. A drunk, basically.

Interviewer: Is this all at a restaurant outside?

2: Yes, restaurants. A small size bottle of around 300cc for lunch and we drink half, like around 150cc.

Interviewer: Is it a different variety [of alcohol] for each day?

2: Various [types of]alcohol for everyday. There is sake, Japanese wine for lunch, champagne for dinner and we just keep drinking and drinking.

Interviewer: Do others of you have the habit of drinking?

3: Most of us are old friends. We are long-term colleagues who will gather together for a drink. [I] only drink occasionally at home. I have some beer, red wine or sparkling wine at night after dinner. For the drinks at our

gatherings, it depends on what the colleagues have provided, [they are] mostly red and white wine and there is also champagne.

Interviewer: Either at home or a restaurant you said, how often do you drink?

3: We usually have a gathering at a friend's place and this gathering is probably about once or twice a month.

Interviewer: Once or twice a month. 4, what about you?

4: I don't usually drink alcohol and have meals together at the same time.

Interviewer: It doesn't matter. It doesn't have to be the time when you have your meals, any time every day.

4: Well, I do drink at home before I go to bed.

2: I suppose it's something strong, taking strong [alcohol] before [you] go to bed helps sleep, it can't be beer which makes you go to the toilet in the middle of the night.

4: Not really, beer also helps but whiskey helps the most.

Interviewer: How many times do you drink a month?

4: I probably drink most of the time except when I am on duty. About 20 times.

Interviewer: That means 5 times a week.

4: Yeah, pretty much. Erm..No, 3 times or 4 times.

1: I had been to wine-tasting classes, I would drink about once a week [at that time]. Then I realize, after the classes I find out that I am actually not so into drinking. In the end, I try to avoid drinking as much as possible. Only when there is, say, my friend's wedding, I will have a little to drink at a wedding banquet like this, or a cooking party at my friend's place where there is alcohol, but I try not to drink whenever possible.

Interviewer: At a friend's place and a wedding banquet. How often is this?

1: It depends. The point is trying not to drink.

4: Why makes you think you don't like drinking?

1: It's all because I feel dizzy whenever I drink too much but not to the point of being drunk, plus I don't want to harm my liver, so I try not to drink.

2: Does drinking have any effect at all on skin?

1: It's probably the red wine which has polyphenols with antioxidant effects. Say the time when I was in Korea, the people there like drinking very much, I went to do my fellowship in Korea. They drink something like Gaoliang, Soju, and they have mixed drinks too. Some combination of Sprite with Korean soju and some other strong alcohol, 3 of these are mixed together and shaken before they ask me to drink it.

2: A drink like that gets you drunk easily.

1: It's scary, mixed alcohol is often very strong.

Interviewer: Has any of you been to a divisional gathering, year-end party or spring reception and things like these? Could you please share this work-related drinking experience?

1: It depends on what style your chief physician has. Our previous chief physician who liked to drink very much and he forced us to drink until we all got drunk, regardless of whether a male or female. The one before the

previous one I shall say. Our previous and current chief physicians both don’t like drinking and they never force others to drink.

Interviewer: It depends on which division you are from then.

3: As well as your supervisor.

1: Yes, exactly. It’s supposed that we don’t like drinking for doctors like us from the dermatology division but it all depends on the supervisor.

2: Almost everyone from the surgery division likes drinking, they love it. Some are into the study of wine, red wine. Both CVS and CV love all of these.

4: The previous chairperson had started this drinking trend.

2: Almost every division has people who like to drink. Now I have kids to take care of, I drink red wine mostly instead of strong alcohol. I will prepare 5SGRS for young doctors if there is any event like the welcome party.

Interviewer: What occasion is that?

2: There is always alcohol, mostly red wine for welcome parties, year-end parties or dinner gatherings of the surgery or other divisions. Strong alcohol is only provided for activities.

Interviewer: Whom do you drink with?

2: It’s mostly the doctors.

Interviewer: Colleagues or teachers?

2: Both. There are always those who are more experienced seniors as well as those of being less-experienced at an occasion like that. Seniors, juniors, chief physicians, superintendents or teachers.

Interviewer: Could you describe what it was like at that time, what was the atmosphere like?

2: The atmosphere eh…. Everyone was definitely shy at the start but we all started chatting freely after having few drinks of alcohol, which helped to ease the tension, and ended up in saying things that we were not supposed to say. (ha ha)

Interviewer: Did you make a toast to each other like what 1 said?

2: Yeah, we definitely had to do it. At the time, I was younger and had to take the alcohol to make a toast to teachers. It was always unfair in terms of the the amount of alcohol we took. The teacher took one sip but you had to drink up one glass, that’s how it was done for most of the time.

Interviewer: Was it like to drink till you get drunk like someone had said earlier?

2: There was always someone who had got to binge drink first, someone who had got to drink till they dropped before drinking stopped.

4: Whoever drops first.

Interviewer: It sounds like the teachers will force the subordinates to drink?

2: Teachers will ask the subordinates to drink more but they won’t make them get too terribly drunk. The situation is mostly that [they are] drunk to the point of being unable to drive but not to the point of lying there and ending up in the emergency room. Well, yes, actually there was once, I was like that.

Interviewer: How do they make people end up like that/

2: It’s like a tradition in Taiwan. They don’t really force you directly but they make a toast to you instead. It’s like you have to drink up this glass. You have

to drink many glasses if every teacher makes a toast to you because you always have to finish one glass for every teacher.

1: Teachers take a sip.

2: Yes, one sip is all that the teachers take but one glass is what you have to drink and it is often like this.

Interviewer: Is it always like this regardless of any occasion? For divisional gatherings, year-end parties, or spring receptions?

2: That’s how you [have to]drink as long as there are teachers and elders. There is even this rule about having 3 glasses for being late [to arrive at an event]. (ha ha)

2: Yeah, that’s right. You have to take 3 glasses if you are late, 3 glasses as a punishment, 3 glasses upon entering.

Interviewer: Only for your division?

2: For all the surgery divisions.

Interviewer: What about the rest of you? Anything like what they just said?

3: I am from the rehabilitation division where there are mostly doctors who are mild. Our supervisor before was female. As far as I know, having a female supervisor means you won’t get to drink that often. It’s usually those young male doctors of the middle generation who would make a toast to each other in private or have an interaction with the young residents by toasting. There wasn’t too much drinking going on because we used to have a female supervisor before at our division. We have this tradition where the younger doctors have to line up to make a toast to the elders for showing respect and gratitude for taking care of them for the whole year. We only do it for the sake of this tradition. Apart from showing our gratitude as a group, the younger doctors like us only have to take a sip because it is the intention that matters.

2: [People] from surgery division will always go together [to make a toast]and drink up one glass together. The elders take a sip while the rest finishes a whole glass. They will have a full glass before they continue to drink and make a toast to the next teacher.

4: There should be tricks for avoiding drinking, or else what will happen to those who get drunk easily from only drinking a little or who can’t hold their liquor well?

2: It’s ok for those who are in the same year as you. [You can] stand at the back. Those who can drink can stand in the front.

4: So [you can avoid] drinking up without people noticing when standing in the back.

2: Yes, [you] avoid drinking up without people noticing and this is what some people will do. Some people’s faces turn red [after drinking]. Once their faces turn red after drinking, they will rest their head on the table pretending they are drunk.

4: A trick of having oneself tortured to win the confidence of the enemy! (To gain their empathy by acting vulnerable)

2: You are still far from being drunk when your face turns red. A face turning red doesn’t mean being drunk because my husband’s face turns red instantly once he drinks.

4: My face turns red at once by [taking]only one sip.

Interviewer: The part about being forced to drink as we just talked about, were there teachers involved?

2: It’s ok for those in the same year as you, we do make a toast to each other but we are not likely to ask others to drink up or something.

Interviewer: Does drinking with someone at a gathering affect the way you see this person? Say, your teacher?

2: Not really. You attend these activities where everyone has fun drinking and gets relaxed to chat. You may see someone being less cautious than they normally show at work but you will still show your respect for them. You will just think a teacher is fun to be with or something once they have started drinking like there is no limit. You tend to be less scared of them. This is the situation seen at the surgery division.

Interviewer: What about the rest of you? If you have met a teacher or colleague who asks you to drink, will it affect the way you see them?

3: I think it’s ok because we don’t really drink massively at our division. Instead it’s probably they have fun drinking with themselves, possibly the doctors themselves from the middle generation. (haha)

2: Lots of stress [are there], they try to make themselves drunk.

Interviewer: The way [they] work normally.

3: The way [they show] normally at work…. It’s a different thing if the doctor also drinks during work, but if there is a clear separation and he only drinks occasionally at gatherings, then I think everyone is ok with it.

2: The teachers are very experienced [in drinking] and they won’t drink until they make a fool of themselves.

3: Unless they are urged to drink a lot because this happens at some other hospitals.

Interviewer: Have others of you met anything like it?

4: No. As said before, it’s got something to do with how your supervisor is like, it should be.

2: You people don’t drink?

3: All of you don’t drink at all?

4: We don’t have anything like being urged to drink.

Interviewer: So how do you make a toast to each other?

4: It only happens at welcome parties.

2: Do [they] lead the young ones to make a toast?

4: Yup, that’s right.

3: Do they urge young doctors to drink at welcome parties?

4: No, not really. This might have something to do with the supervisor, otherwise there should be no difference[whether there is being urged to drink or not].

3: I think for those, for some supervisors, who binge drink are also those under a lot of pressure. (ha ha ha)

2: It’s pretty much the same for everyone having a lot of pressure at surgery division.

Interviewer: 1 talked about how teachers urge others to drink, will this affect the way you see them?

1: I think it might. For those who urge me to drink, I will also think of them as someone who likes to force others. I think of my senior as a good fellow who helped me avoid drinking when I was in Korea. (ha ha ha)

Interviewer: Does everyone think drinking at gatherings will affect your work? [Is your work] affected? Sometimes you have to drink at a gathering for work. Will this have an effect on work?

1: I think it probably depends on how often [you drink]. There is probably no effect for people like us who don’t really drink.

2: It’s not really often for [me to] have work-related gatherings. How often [it is]to have a gathering with your friends in private, a personal gathering, is different for everyone. [I] have work-related gatherings for about 3 times a year, the welcome party, year-end party and spring reception.

4: Yup.

Interviewer: [What about] with colleagues?

2: We arrange our dinner gathering in private. For colleagues from surgery division, it’s hard to arrange the best time for dinner gatherings. We manage to have a dinner gathering for only once every two months on average. We don’t often have a drink with our colleagues or in private and neither do we drink often at work-related gatherings.

Interviewer: What about the situation as you mentioned where you are forced to drink by others?

2: With colleagues from work or in private?

Interviewer: We talked about being urged to drink, is this drinking culture going to….

2: You drink when you want. It’s not like you don’t care too much for your friendship if you don’t drink, no, nothing like this. It’s often you get urged to drink at work-related gatherings.

Interviewer: In the case where you always get urged to drink, [do you think] it will affect work?

2: No, it won’t for sure. I don’t get drunk that easily. I never refuse and I will drink if I am urged to drink. For some people who get drunk easily, the teacher has no problem with them if they refuse to drink. The teachers probably only have a laugh about how they can’t drink or hold their liquor well, but will not question their work ability or think they don’t show their respect.

Interviewer: 1, do you think being urged to drink will have an effect on work?

1: The only occasions for doctors at our division to get urged to drink are only during welcome or year-end parties, even with the one before our previous chief physician who really liked to urge others to drink, there was actually no effect on our work. We are not like those from the surgery division who usually go out for a drink; we ain’t like that, no. (hahaha)

4: Do you often have dinner gatherings?

2: It was quite often when I was a resident.

Interviewer: Do others of you think [drinking] will affect medical practice?

3: There are not many times[for us to have a drink]. We only drink at major events like year-end or welcome parties, or drink with our own friends in private.
Interviewer: You drink with your friends in private. Does this will have an effect on work or your daily medical practice?
4: when we drink in private or…?
Interviewer: Since you don’t have many gatherings, what about when you drink in private with your friends?
3: It’s ok. We only drink on weekends and there won’t be any effect at all.
2: Everyday is a weekend, everyday is a workday, it’s tiring! It’s tiring for moms.
Interviewer: 4, do you think drinking at a work-related gathering will [affect] work?
4: Whether it will affect….I think someone who is affected easily then they will definitely get affected. They are said to be those without a good control of themselves. This is similar to talk about someone who smokes or something. Some people do really show behavior out of line after drinking but this has got nothing to do with our work-related gathering, they are just meant to turn into another person wherever they are.

Interviewer: Will you arrange drinking occasion if you hold your own dinner gathering?
1: I will never want to drink if I hold my own gathering.
2: I will prepare 3 to 4 bottles of wine if I hold my own [gathering]. I definitely will prepare. It’s [The wine prepared] for drinking at the beginning [of the event]. Absolutely, red wine, white wine and then sweet wine.
Interviewer: 2 will prepare alcohol, what about the rest of you?
3: Usually they are very close friends [whom we have gatherings with]. Everyone takes turn to prepare [alcohol]. My husband prepares [alcohol] because we have home gatherings mostly.
Interviewer: At a divisional gathering or..?
3: The hotels will usually provide alcohol for sure, if it’s for a divisional gathering. We usually ask [if there is anyone who drinks] and make the order [for alcohol] part of the entire cost of the dinner for the table.
Interviewer: That means you will also prepare alcohol.
3: I won’t prepare any strong alcoholic drinks, but probably red wine or something.
Interviewer: 4, will you [prepare alcohol]?
4: Usually for us…actually it depends on the locations we pick instead. There is no alcohol for some places where no alcohol is provided then [we] won’t specifically ask for alcohol. There is no alcohol provided for places like the all-you-can-eat restaurant. We will order alcohol at a restaurant where there is alcohol provided.
Interviewer: So it depends.
3: There is one buffet [all-you-can-eat restaurant] where alcohol is provided! Tsun something Tien (trying to recall the name of the restaurant).
4: Da Tsun Chiu He.
3: All-you-can-drink.
1: There is an extra fee for drinking.

3: Not many buffets have this.
4: [I] have been to one buffet where there is no extra fee for alcohol. You can help yourself at the beer dispenser
2: Only beer [is there].

Interviewer: We just talked about making a toast to each other. What do you think the motive is for this type of drinking behavior when [you] have to drink at a gathering for work? What is your personal view about this?
2: For me, I think it’s probably to show being friendly.
(Everyone laughs out loud)
3: It’s only because you like to drink.
2: This is not bad! “Take one more sip and one more sip” as a way of being friendly and hoping everyone to relax and be less cautious. Everyone can chat freely but certainly not to chat about what’s on their mind. It’s like hoping everyone can relax while going out to eat. There’s also an entertaining effect.
Interviewer: You think it[drinking] helps relax.
2: [It] helps relax and builds up a closer relationship. Everyone becomes closer [to each other] and it’s easy to talk about things when they get more relaxed [after drinking].
Interviewer: 1, what do you think?
1: I also have the same view about this. I think it is related to culture. Like when I was in Japan or Korea, the people who were normally cautious became quite relaxed after drinking and it was easier to talk about things.
2: What a huge difference! Scary.
1: It was really different from [what they were] at work. In the Japanese or Korean culture, you can see how they normally show pretty deep respect to their teachers, but you can get to see the different drunken behavior of everyone after drinking there, it’s pretty funny.
Interviewer: Anything unforgettable?
1: Disinhibition.
1: Like the senior I had at that time, he started to feed me fried chicken [after he got drunk].
(Everyone laughs)
Interviewer: It’s different after drinking.
1: At that time, we were like, “Wow, what a drinker as being the one from our dermatology division.” This is what we said during the day.
Interviewer: Did this affect the way you see him? In a better way?
1: It was funny. I just thought it was funny at that time. I had teased him about this for few days [at that time].
2: After that incidence, it was easier to get along with him [for you]. You probably had less pressure of spending time together with him after knowing what he was like when he got drunk.
3: No one from our division has been drunk. It was only during internship where there were people drunk. I think there are always people who like to drink, so it’s ok for everyone [to drink] at a gathering. It’s normal to drink alcohol with meals anyway. One thing in particular to mention is that when the supervisor

gets drunk after dinner, he will make a phone call to everyone in the division at midnight around 11pm or 12am, it's hilarious and you will get his call at midnight.
(hahaha)
3: He talks crap to you and he's already drunk. You know he's drunk!
4: Did [you] get his phone call at home?
3: Probably he had so much fun drinking with everyone and he also got drunk. He would make calls to those who didn't go [to the event] asking, "How come you didn't come?"
Interviewer: Will this affect how you see him?
3: But [we] have known him for many years. Everyone in the division had received the phone call.
4: Has he been like this for many years?
3: No, [it happened]probably only once every few years. After it had only happened for the first time, everyone started talking about it and the rumor spread. The number of people involved was too great because phone calls were made according to the list of phone numbers for the whole division. Everyone in the division got the phone call.
2: It was so funny.
(hahaha)

Interviewer: Has any of you seen anything ridiculous like this?
2: A teacher who normally seemed serious kept adding songs [to the karaoke list] at a karaoke bar or kept singing even when the song was finished. He was, he seemed to be a little hyper.
Interviewer: 4, for the motive of drinking at work-related gathering, what do you think? We talked about how it helps relax and builds a closer relationship.
4: I shall say work-related gathering is no different from the one with friends; there is no motive or anything involved.
Interviewer: We talked about making a toast to each other and things like this.
2: Let's put it this way, if we have to talk about it, it's nothing like we really want or we want to do especially, to have a motive.
3: Drinking is [a] natural [thing].
4: There is no motive in particular.
2: Drinking sometimes is not the main focus at a work-related gathering. Sometimes it's like stealing someone's thunder for [anyone] drinking too much. The purpose for a gathering is to build up relationships and use up the budget allocated to the division; this is what it is all about.
Interviewer: It helps build a closer relationship.
2: Pretty much.

Interviewer: Will you continue to do so, to prepare alcohol that is, if you become the supervisor for your department?
1: For me, I won't because I don't drink. It depends on the personal interest. I will, for my wedding. As a supervisor for the department, I will take the type

of the restaurant into consideration, I won’t prepare [any alcohol] if the alcohol [provided there] is expensive.

Interviewer: Anything more you’d like to add?

2: What’s interesting at a formal dinner gathering for work is that doctors tend to get together with doctors instead of nurses. But few doctors might go to a dinner gathering and have a drink together with some nurses in private. At formal [events] like spring receptions or year-end parties, doctors and nurses don’t drink together because there seems to be some distance between them. The doctors drink with doctors and nurses drink with nurses, this is a situation more commonly seen and it’s something strange. If say in private, after operation, few doctors or nurses go together to have some beer at a fried-dishes serving restaurant where everyone drinks together, this is more fun.

Interviewer: Anything else?

Group 10

Physicians (n=5)

Group 10

Interviewer: Let’s get to know each other by starting with a self-introduction. Shall we begin with your favorite types of food, anything from Japanese to Western style of food?
5: Hi everyone, it’s my first year at this department, a new comer who likes Chinese stir-fry dishes.
4: I am pretty much into all sorts of cuisine. I have Japanese food quite a lot, the hot pot comes second.
2: Japanese food is what I usually eat the most compared to the rest.
Interviewer: It seems that doctors like Japanese food quite a lot. Would any of you have the habit of taking alcohol with meals?
4: Occasionally. I drink when my wife is not around. I won’t be allowed to drink when my wife is around.
Interviewer: Where do you drink?
4: [I drink] when I am outside. I will drink when I don’t have to drive! I won’t if I have to drive.
Interviewer: How often do you drink?
4: I don’t know. It depends on my wife’s mood.
Interviewer: …roughly how many times a month?
4: about once or twice [a month].
Interviewer: So it is once to twice a month. What about the rest of you [other doctors]?
2: None. [I will drink] only when it’s compulsory such as at a gathering.
Interviewer: This means normally you don’t take any alcohol regardless of having meals or not.
2: That’s right!
5: [I drink] before sleep or after meals. [It would be]anything between 3 to 5 days per week. I drink at gatherings, with meals or even without meals.
Interviewer: For what occasions?
5: No, [it is]not necessarily[for any occasion]. It could be anywhere from restaurants to homes.
Interviewer: How frequent?
5: 3 to 5 days a week.
Interviewer: You seem to drink more often [than the rest]. Red wine?
5: I am like an alcoholic. Red wine, beer, whisky, white wine and vodka are all in my list. There is no exception.

Interviewer: Would everyone share the situations where drinking is work related? Such as year-end parties or spring receptions.
2: Yes, exactly!
5: Indeed! You said it.
Interviewer: I meant to say to describe what it is like at the year-end parties. I haven’t been to one personally.
5: The male seniors would lead us forward.
4: Year-end parties. Alcohol is seldom provided [as I recall] because the majority of us from our division doesn’t enjoy drinking. It is more like to use those soft drinks instead of real alcohol for making a toast.
Interviewer: At year-end parties or spring receptions, there is always….[drinking events going on]

5: Not most of the time. The frequency of drinking in neurosurgery division is low. Drinking occasion is mostly seen during residency or at spring receptions and year-end parties held by the surgery division.
Interviewer: For the drinking events at the year-end parties, could you describe to us more in details?
5: The seniors would lead you to make a toast then you would have to follow what he does.[a toast] to the teachers, yes, that’s right.
Interviewer: The seniors would take you to make a toast to the teachers.
5: This is politeness.
Interviewer: And the teacher will also…How is it like to make a toast to one another?
4: [Start by saying something like]Hi, teacher or Hi, Doctor with their name. You start making a toast to the person of the highest rank, Chief Physician with their name. Everyone would have some chat, a toast is done to everyone according to the ranking order, then we would switch to another table. We sometimes make a toast to the nurse practitioners of the ward or the female seniors would take turns of making a toast to one another. By doing so, we are showing our gratefulness to them for handling matter for us at work on a daily basis, an appreciation to everyone.
2: Thanks to everyone for taking care of us.
Interviewer: All of you would show your gratitude for the seniors and the teachers, does this happen at the year-end parties?
5: [It happens] Either at the spring receptions or the year-end parties, basically any large scale social events.
4: Teacher appreciation banquet. The teacher appreciation banquet that takes place once a year. This is also when the chief residents are promoted.
2: Ya-Jui Lin didn’t drink at that time last time[of the last teacher appreciation banquet].
4: Hardly any [drink]!
Interviewer: Could you talk about the drinking occasion?
4: relating to work?
Interviewer: any gathering within your own division is also fine.
5: We don’t drink at gatherings held by our own division.
2: Yeah, we drink juice instead.
4: Alcohol is replaced by tea.
Interviewer: We didn’t talk much on drinking at the year-end parties. Is there any other drinking experiences, like with your colleagues or..?
5: Colleagues’ wedding!
4: Colleagues’ wedding
Interviewer: Do you have any unforgettable experience? I believe there is, more or less…
5: What do you mean by “an unforgettable experience”? Things like getting too drunk and losing it, and beating up the teachers? Or..
Interviewer: Anything will do.
5: Yes, there was once a welcoming party for R1 at the division of surgery. Ming-Che Hsin was about to throw up in my car and he did. That was some unforgettable scene for sure.
Interviewer:3, we are discussing about whether or not you have the habit of drinking, do you normally have your meals together with alcohol?
3: Not usually! It is more likely when I am at a social gathering.
Interviewer: Could you give us an example?

3: [I will drink] such as during the welcoming parties or year-end banquet, when I don’t have to drive. We have fruit wine or something like that at home, we don’t have it every day but only on gathering occasions.
Interviewer: you keep and drink fruit wine at home.
3: When going out for gatherings, those mentioned earlier.
Interviewer: Any other doctor with unforgettable experience to share is also fine to mention anything earlier in the past or from other division.
4: I remember there was one gathering for all the national residents of neurosurgery. There were two days after the main presentation day. On the first evening of dinner gathering with all these other hospitals , it was really like drinking to the point of throwing up because you were being forced to drink.
Interviewer: You were being forced to drink. Being forced by..
4: the teachers from other hospitals, you would try to build a bond with them because they might become your future interviewers, you will drink as they ask.
Interviewer: it’s like being compelled to drink by the higher ranks. How would they do this?
4: when they raised their glasses and you were obliged to drink. How would you skip your drink if they have finished theirs?
Interviewer: So it is like making a toast continuously and consistently.
4: there were like more than 10 teachers around. Every teacher had their own turn to make a toast and it ended up a lot of toasting made after one round.
Interviewer: Every teacher was like so?
4: almost every one of them.
5: yes, most of them.
Interviewer: Had the rest of you met something like this?
2: same thing happened in the society of my division.
5: It happens most of the time.
Interviewer: Would this have an effect on your views towards your teachers? Thinking that this has an influence on his professional image?
5: They are like that as what we thought of them personally.
2: It’s ok if it only happens occasionally.
Interviewer: Being occasionally is ok. What about others who think their views towards their teachers have been affected?
4: It is often something that occurs once in a while but not frequently.
2: Once a year.
Interviewer: It’s to say everyone is ok with it?

Interviewer: Are you thinking the same thing, 3?
3: yes, similar. Drinking is more likely on the occasions of year-end banquet and society gatherings as mentioned earlier. I try to avoid myself from it, and it usually works if I say I have to drive.
Interviewer: whom you are drinking with?
3: Teachers or colleagues. Colleagues mostly at year-end parties. I haven’t had any alcohol to drink on the occasion of any year-end banquet. Doctors were more likely to drink when the parties were used to be held nearby in the past and they would attend easily by walking. If the parties these days have been held at somewhere far away and doctors are less likely to drink as they have to drive to the parties.

Interviewer: There were more stories like this to share from the past. It is fine to describe more from the past to us.

3: Similar situation was seen. In the past, physicians would come to ask you to drink with them or you had to make a toast to your teachers. It was hard to avoid. This is different comparing to the present where more consideration is seen among colleagues. Different era presents people with different perspectives. In the present era, there are a lot of negative reactions to drunk-driving as compared to the thought of alcohol having no influence on driving in the past.

Interviewer: when we were talking about being forced by teachers to drink. In that case… would any of you doctors ask them, I meant your colleagues or teachers, to drink?

2: “We “ask others to drink?! No, we wouldn’t dare. It is too difficult!

4: When they make a toast to us and we would then drink in response so we won’t ask them to do s.

3: Making a toast to one another. If you mention that you have to drive, you would drink with them using juice instead of alcohol, and this is how it is.

5: No, I won’t.

Interviewer: Would drinking at gatherings have an effect on your general views towards your colleagues and teachers? About the way you see them normally, would drinking at gatherings make you think …..?

5: It is ok.

3: There is not much of a difference! However, we don’t know how the teachers view towards us or what the teachers think of us. We talked about the present era when we tend to avoid drinking with the excuse of driving despite the teachers making a toast to us. We wonder what would the elderly teachers think as we didn’t drink in response to their toasting. We will never know.

Interviewer: I would like to know what you think personally.

3: Say if I make a toast to them and they don’t drink in response. It wouldn’t matter to me.

2: There is no big deal because we are colleagues.

Interviewer: The teachers are more likely to compel others to drink. In this case, would drinking at gatherings have an effect on medical practice? In other words, would your regular work be affected by it?

2: There is not many gatherings going on at our division. We won’t attend if it’s the day on duty.

3: unless you were really being forced to drink and too drunk to wake up the next day but this hasn’t happened.

2: It was rare. You wouldn’t go when you are on duty. I don’t know about the next day, I think it’s not affected.

3: I haven’t heard of it so far. Drinking affects your work the next day, no, I haven’t heard of it.

Interviewer: what about the rest of you?

4: Same. There is not much chance of drinking here at our division. Not many gatherings to start with.

Interviewer: Rumor has it that people of the surgery division totally nail it when it comes to drinking.

4: Yup! We are from the same division. This is what our culture like. I don’t know about other divisions. There are not many gathering events. We rarely drink that much alcohol even if we are at gatherings, not to mention an even lower chance of drinking after work.

2: We don’t drink privately either.

5: Same. We are all of the same division.

(Everyone laughs)

Interviewer: [how about drinking] with other divisions?

3: Not very often.

2: It depends.

3: If you drink and you are more likely to meet the one who also does. There is not much drinking going on between our division and the others. What I know is some people having other relations such as Dr. Chang, he has, not doctor-patient relationship, but this relationship with other friends, and these people don’t have to be medical coworkers.

Interviewer: Pardon me. What did you say it was? A doctor-patient relationship?

3: Not doctor-patient relationship. His colleagues. His own social circle and he has abundant friends, making him more likely to drink.

Interviewer: What do you think the motives are for the drinking behavior, having to drink for a work-related gathering? What’s your point of view? Like the behavior we mentioned earlier on being urged to drink.

3: To build up a better relationship. Having meals together is pretty much the same as meeting and greeting to each other on a daily basis! Perhaps drinking helps you talk more than you normally would, being able to say something you wouldn’t dare to say normally.

2: Some people might simply enjoy drinking alcohol and they could use it as a good opportunity to drink.

Interviewer: So you think the doctors could get to drink the alcohol as they have wanted.

2: Yes, it is to drink only on certain occasions. It is an opportunity to drink alcohol. Mao-Yu Chen seems to be very capable of drinking.

4: He seems to buy alcohol quite often

3: She drinks. Ching-Yi Lee buys fruit wine.

4: They do drink. Making order for others.

3: Dr. Chang drinks with others. I think it is a doctor-patient relationship. It is ok if this drinking relationship occurs among colleagues. Some patients, his patients might be his good friends. You never know if there is any kind of this drinking relationship going on.

Interviewer: 1, do you keep a habit of drinking normally? Care to share experiences such as those of gatherings from the division or year-end banquet.

1: Wine tasting is often seen at our division. Our director drinks red wine and enjoys wine tasting but never drinks in excess. He never has too much to drink.

Interviewer: We shall say that your director is a great drinker.

1: Our director drinks red wine. He has a wine rack at home. There has to be a theme for wine-tasting every time we go there each year. Like the theme for last year was Burgundy red wine, lots of red wine. The theme for next year is New Age Red wine. We would have to listen to stories about his personal interests at the year-end banquet every year.

Interviewer: this was during the year-end parties….

Group 10

1: There is tape recording going on, is it? It’s kinda scary. Will Ching-Yi Lee beat me up?
Interviewer: No, you don’t have to worry. The information recoded is confidential.
1: Will she beat me up for not telling enough? I might be picked on and obviously vulnerable in the field out of neurosurgery in the near future.
Interviewer: As I said we won’t make our information public. It [Interviewee] will be presented as numbers of 1234 and so on.
Interviewer: Back to earlier [topic], was it only year-end banquet?
1: He would wish to “preach” at us when he invited us to his house every 3 to 4 months. There was often wine involved. The type of wine [given to you] differs depending on whether or not you were born in the 70’s, being a resident or an intern. Take me as an example I did poorly this year. He would give me the wine of the lowest quality. He would offer you [drink from]a bottle of [$]5000 if you have the best performance this year.
2: It is only through wine tasting to know how well you have performed. People like us who don’t know much about wine-tasting won’t feel the difference.
1: If any resident feels like to poison themselves to death, they should be given the industrial alcohol.
Interviewer: This happened at his home?
1: yes, we go to his place.
Interviewer: How often do you drink normally?
1: This somehow has become my own exclusive interview!
5: We had been asked and through the same thing.
1: As for myself, I drink red wine.
Interviewer: Where do you drink?
1: At home. Not a drinking club. I am a good father.
(Everyone laughs)
1: A happy and wonderful family. I drink at home with my wife.
Interviewer: May I know, if it’s possible to be at a restaurant, then how often in a month will you drink?
1: about once or twice a week.
Interviewer: Having talked about the year-end banquets, would you care to talk about gatherings within the division or things like spring receptions.
1: Sales people from the companies often offer free meals. [Talking about this secret] The word of this might have reached the chairman of committee.
(Everyone laughs)
1: It’s so scary! Will I get fired? It’s terrible! No, it’s not like so. Only very little [was sponsored]
Interviewer: Would you describe what it was like relating to work?
1: This would be the last time in my life.
2: Don’t stop eating till you’re full.
(Everyone laughs)
1: There is only my wife, myself and my two daughters in my family.
Interview: It’s not you and other doctors with the sales people from the companies at your place instead?
1: We can’t be saying things randomly out of anything.
3: There has been hardly any [free meals] these years.
2: Coffee is mostly offered by those companies.

Group 10

4: Coffee

Interviewer: So 1, you and the companies….?

(Everyone laughs)

1: We had some social functions at the academic events sponsored by the companies. We had to drink at those social functions held after the academic events. Everything complied with the hospital regulations.

Interviewer: [meal gathering] with the companies. What about gathering within the division?

1: yes, yes, there is. Occasionally.

Interviewer: Whom are you with [in the gathering]?

1: Teachers, colleagues and male juniors.

Interviewer: How often do you do this gathering within the division?

1: There is not much socializing [in our division]. About half a year to one year.

Interviewer: Please tell us more, things like year-end banquet, spring receptions or welcoming parties. Any unforgettable experiences? Any welcome parties?

1: No welcome parties. Nothing much. We are boring people. We shared the history, origin, and label reading of wine. Wine tasting was an academic event to us. We had a wine taster. We would invite a professional sommelier who served us wine and introduced wine to us.

Interviewer: during the year-end banquet

(Everyone laughs)

1: I think this interview was not carefully designed.

2: You are being recorded.

Interviewer: As some other doctors mentioned earlier about being urged to drink by teachers, did this happen to you too?

1: No, never. We personally enjoy drinking. We drink on our own. We don’t have to be forced [to drink] because all the wine we have is very tasty.

Interviewer: That means you will drink it no matter what without being forced to do so.

5: We urge ourselves to drink.

1: [We]Can’t leave without signing for the last interview.

(ha ha)

Interviewer: We talked about the people involved [in the gathering] were colleagues or teachers. Did you probably get to see a different side of them possibly during the drinking events of the gathering? Would this affect how you think of them normally?

1: Yes. They would seem to be more talkative, speaking in a more academic way, more open-minded and free to speak their mind. Would this do anything to your report? Such as if someone holds a grudge against a certain person, they would start scolding this person after drinking.

(bursts into laughter)

Interviewer: Being more talkative and including scolding?

4: [It’s] more like a greeting.

1: It’s showing expectations for them, hoping that they would do better.

(Others laugh out loud)

Interviewer: Meaning to have a better impression of him.

(ha ha ha)

1: I am the only one being asked.

2: There is punishment for being late.

Interviewer: Other doctors are free to add anything more you like.
4: We had been through the same thing. We share similar viewpoints.
1: There is no standard reply to look up. I tend to shoot my mouth off.
(Everyone laughs)
Interviewer: It doesn't have to be the same as those of the others. It depends on personal point of view.

Interviewer: Would any of you personally prepare wine for the year-end banquet or divisional gathering? Meal gatherings are held by your own division.
5: We haven't had any meal gatherings in our entire division.
3: It depends on the people who attend, see if there is any drinker in there. Someone who enjoys drinking would bring their own [alcohol]. But this is unlikely in the case for some nursing departments with family members attending. It depends on the department or division.
Interviewer: 3, you prepare your own alcohol?
3: If invited, I will drink depending on the people involved. If I feel like drinking, I would prepare alcohol for them at meals. I would not prepare it If attending something like the one held privately by the intensive care unit, where there would be nurses, their spouses or kids.
Interviewer: What about the others? Would you [prepare the alcohol]?
5: Depending on the people involved.
3: I suppose you normally do.
5: I did prepare white wine for that teacher appreciation banquet the other time. Dr. Lee loves to drink white wine. Both Hung-Yi Lai and I prepared.
Interviewer: 1, How about you? Do you prepare alcohol for the divisional gathering or year-end banquet?
1: Yes, I do. I will buy it from Costco. Were you asking about where to get it? I prepare both red and white wine, as well as some liqueur. The order of drinking alcohol is to have champagne as the aperitif, champagne with the dessert; white wine with the salad as the appetizer; expensive red wine with steak then comes after. We to classes for this [wine tasting course]. We had learnt a set of course [about this]. We take wine-drinking very seriously.
2: You have a wine rack at your house?
1: NO!
5: You are free to put them in my house if you wouldn't mind.
Interviewer: So you doctors do prepare alcohol.

Interviewer: Would drinking at work-related gathering have any influence on your work?
2,4,5: We all had answered to that.
Interviewer: Everyone could add anything more to it. It's ok.
1: Drinking will affect the next day so I don't drink while on duty. Definitely no drinking on the day being on duty. It does affect work, is that what you were trying to ask? (laughs)
Interviewer: I don't mean to drink while on duty but being at a meal gathering for the sake of work.
1: If it will affect the work the next day we will only drink on the weekends, when we find free time to relive stress on Saturday or Friday

Interviewer: We talked about being urged to drink or urging others to drink, and making a toast to one another. What do you think the motives are for this drinking behavior? What's your personal point of view?
2: I had made my point before. It's your turn.
1: the motive for urging others to drink?
Interviewer: this kind of drinking behavior.
1: This is deep!
4: It depends and is different for everyone. Some people are into social drinking while some enjoy watching others being drunk. Everyone has a different motive.
1: For us, we just like to build a closer relationship or better friendship. You tend to be more open-minded after drinking.
(ha ha)
Interviewer:1, you have a similar motive to 5's. 4 preferred to say [motives are for] different intentions.
4: We don't know exactly what their intentions are. Perhaps they would like to build a closer relationship or..
Interviewer: to see them making a fool of themselves. What is your personal view of this?
Interviewer: There were few doctors saying that, 1, you prepare alcohol for events and activities, would you still do the same once you become the director of your department? Will you still prepare alcohol or you will make a change?
1: I will only know when I really get to that position! I would have to try it first.
(ha ha ha)
1: At the moment, I can't tell. It's too far from now.
2: As for myself, I don't drink. It is highly unlikely but I am not against anyone who does drink. As for me, I won't prepare alcohol because I don't know anything about it.
Interviewer: you won't prepare alcohol for any events.
2: Yeah. But I am ok if anyone who likes to bring alcohol along.
Interviewer: Lastly, I heard most of you talking about urging others to drink or being urged to drink, is there any more situations like this to talk about? Would it affect professional image if any of you has seen it happening at any drinking event?
4: Making drunken faux pas?
Interviewer: Yes. Something like continuously urging others to drink.
5: For someone who normally appears decent but acts like a scum after drinking?
Interviewer: Not that serious. Would it affect a doctor's professional image?
4: Are you saying the behavior of someone urging us to drink?
Interviewer: Yes, or anything like this you have seen.
1: The reason for Dr. Lee inviting me to this is to see me making drunken faux pas after drinking. You may ask her about what she thinks of me making drunken faux pas after drinking.
(ha ha)
1: Yes, it will have an effect. If I am not familiar with that person, it would be my first impression of them after seeing that kind of situation.
Interviewer: Doctors will urge others to drink. This would have an effect on their professional image. What are your views of this?
1: I think we have to speed up. He didn't get the information he wants. What other influences are there, did you say?

Interviewer: Your personal view.

1: Make sure you don’t make drunken faux pas, try to control yourself. Under the influence of alcohol, you may speak your mind unintentionally with your colleagues. You don’t mean to but people will remember what you say to them. A serious drunken faux pas will alter the relationship among colleagues and have some effect on your professional image. It is therefore important to drink moderately. I made a pretty good answer, didn’t I. I have a meeting at 12. I have this research assistant who is in pain.

Interviewer: What about the rest of you?

4: Ditto

5: Ditto

(ha ha ha)

Interviewer: Any other personal views?

3: The way they perform their clinical practice will still be observed. To consider making a clear boundary between private lifestyle and work. You can drink whatever way you like but make sure you handle it well, nothing like drinking in excess or alcohol abuse would happen. As for myself, I only hold one single attitude [towards this issue]. If I occasionally bump into some doctor who seems having too much to drink earlier at the outpatient unit, there is no room for discussion, I would ask him to control himself straightaway. There are two sides of viewing this issue. We will think less of him if drinking has affected his work and professional image.

Interviewer: It is to say that you all believe there is an influence of it more or less. Lastly, would anyone like to add anything?

Group 11

Physicians (n=4)

Group 11

Interviewer: Please introduce yourself with your position title. Anything you like to say about the type of food you love to eat. Something like this.
4: Meat
Interviewer: Other doctors?
1: Everything is good. Vege, meat, fruit, nothing in particular.
Interviewer: Japanese cuisine…
1: Both Chinese and American dishes are ok. [I am]Not particularly fond of Japanese cuisine.
3: I like anything that is delicious. No particular regular food.
Interviewer: You are not picky about what to eat. Do you drink normally with your meals? Or even without meals.
4: No. It’s mainly for socializing. I don’t drink for nothing specific but mainly for socializing, is there such an answer?
Interviewer: You’re saying that you don’t usually drink.
4: I won’t drink for nothing in particular. It’s only for social purpose. Drink only when necessary.
Interviewer: How often is this?
4: It depends. About once every 2-3 weeks.
Interviewer: About once every 2-3 weeks.
4: Yes. To be quantitatively. This study needs to be quantitative instead of qualitative. Being qualitative means yes or no. For being quantitative, you know, something like once every 2 weeks; 300 c.c. at a time of some beer or what and things like that. I have a feeling that this is not included in your questionnaire. So let’s keep it this way. You didn’t design this questionnaire, did you?
Interviewer: 3, what about you?
3: I am from the control group. Nil. I don’t drink. All the neurosurgeons, all the doctors of this specialty don’t drink. I make a toast with juice instead of alcohol. I am in charge of “control”.
(ha ha ha)

Interviewer: Every one of you who has attended year-end parties or things like spring receptions. Would you please share what it is like at those events?
3: Alcohol. I felt embarrassed. I was the only one being sober. Everyone was drinking. I didn’t make a toast to the seniors and teachers with alcohol but juice instead. I felt embarrassed but I really can’t drink.
Interviewer: The rest of you are better handling [drinking situations like this]?
1: I wish I could be in the control group too. I was pushed every time and ended up drinking at least some alcohol. I wonder how 3 could be the control, who has been in the control group since a younger age.
3: Try to suck it up. This is like during the time serving in military when something is up.
1: You totally nailed it to be able to hold on to the very last moment.
Interviewer: Whom did you drink with? Who are those people you usually drink with?
1: Those who are elder or of higher ranks. Seniors, bosses and teachers. You would drink as what they tell you to do.

Interviewer: Any colleagues [involved]?
1: The colleagues won’t make you drink. It’s less likely for the peers to do so. We are classmates and we don’t urge them to drink.
Interviewer: But those teachers or seniors would. How is it like to make a toast to each other?
1: Simply raising a glass to make a toast to them.
Interviewer: Would they ask [you to do so] consistently?
1: They are holding a drink but you are not. Sometimes they would pour you drink if you want to have some.
Interviewer: That means you would keep on drinking even if you don’t really feel like it.
1: It feels better to do so. It’s what I think because all the others are drinking.
Interviewer: Even the teachers too.
3: There would be differences as times have changed. During the time when I was a resident [as compared to], the present day where democracy encourages people to respect personal rights and interests of others. Not many residents [nowadays] will be willing to drink, the times are changing. Drinking behavior, drinking courtesy and drinking with the most powerful person in Taiwan. As for myself, I don’t drink either. I might be kicked out [if this happened] in the past time (ha ha ha). There is a difference as times are changing. You could hardly see anyone being forced to drink in the current society, or at least in the profession like doctors nowadays. I am not sure about the working class but it is the case for doctors regardless of the relationship being between the peers or 2 different ranks.
Interviewer: What about 4?
4: We respect each other. Our bosses would only do it for the sake of it! To be honest, you just drink as you feel like it. They would pretend to demand that you should drink but in fact, we won’t drink. This is how it is like. Just my opinion. Even the bosses know about this. He won’t really force you.

Interviewer: 2, we are talking about the drinking experiences from things like year-end parties and spring receptions. Asking whether you usually drink or not, or you would like to share experiences from work-related gatherings.
2: Yes, I do. I do drink a little.
Interviewer: you “usually” drink a little? How often is this? About how often a month..?
2: Something like every week.
Interviewer: On what occasions?
2: I definitely drink at gatherings. It’s a must.
Interviewer: At restaurants? With whom do you drink?
2: Yes. I drink more or less but not too much during meal gatherings with colleagues and friends. I also drink a bit at night at home.
Interviewer: Colleagues and friends. At restaurants or home. A lot of you mentioned the divisional gatherings as the work-related gatherings, is there any for the year-end parties? Would you care to tell more about what it is like?
2: We don’t drink at year-end parties. We don’t drink if Dr. Lee doesn’t.
2: Basically at our year-end parties, we will drink only if the bottles are opened. Even if there is no alcohol, we will not think about ordering it. We will not

drink and drive, and things like that. We will walk or go by taxi to drink and eat at the parties if they aren't too far. We will drink if the bottles are opened and we won't particularly order alcohol to drink.

Interviewer: Could you provide us an example of your drinking experience or anything similar? What was it like before to have a divisional gathering or year-end party?

4: Everyone got along happily and well together and something like this.

Interviewer: Yes.

2: Colleagues and teachers or seniors from other tables came to make a toast, we would do the same in response to their toast and we felt happy.

Interviewer: with the elders and seniors.

2: Some of them are also colleagues. We would go to the others, make a toast to each other and send our blessings to them.

Interviewer: How did you make a toast to each other?

2: "Hey, doc, let's drink or take a sip, and happy new year everyone" like this.

Interviewer: Would the elders ask their subordinates to drink?

2: They wouldn't. They wouldn't ask someone to do so if this person has to drive.

Interviewer: What about the rest of you?

4: We would respect each other. You are also free to drink more.

Interviewer: 1 mentioned that some people will urge others to drink.

1: Yeah, they would try once or twice [to urge you to drink]. I think it's ok If you would be able to avoid yourself from drinking.

Interviewer: Would you ask other colleagues to drink?

1: I wouldn't deliberately ask others to drink. I wouldn't ask others to do so if I don't even drink that much. If others insist that they won't drink then I am ok with it.

Interviewer: You won't deliberately ask others to drink.

Interviewer: Do you think you will change your views towards your seniors or colleagues if you have experienced drinking with them like we mentioned earlier?

4: Our views meaning…?

3: Making a fool of himself or herself if there is any. Would this affect the way a person sees them.?

Interviewer: Would you think differently after [seeing them] drinking at a gathering, or change your views on their work performance?

4: with or without seeing them making a fool of himself/herself?

Interviewer: anything will do.

4: Whether or not his drinking behavior would affect the way I see about him even without seeing him making a fool of himself.

Interviewer: Would 2 have more experiences like this?

2: It's usually a fun occasion. You would occasionally bump into some people who have too much to drink and get a bit hyper. Everyone knows and feels happy. It's ok once you are sober. I haven't seen any drunk brawl. After drinking, everyone feels happier but never gets drunk or talks crap. Despite being more talkative they are still conscious.

4: Talking too much leads to improper doctor-patient relationship.

Interviewer: Drinking has no effect on your views towards them.

Interviewer: 1, you talked about how the seniors demanded that their subordinates should drink, would this affect your views towards them? It doesn't have to do with them making a fool of themselves.

1: I think not. Technically, I wouldn't use the word "demand" here. They might say something to you like "Come on, drink! Take some! It's ok!". There is a difference as times have changed, like 3 had mentioned. I would say it was more like a "demand" during my time of R1. I haven't felt the pressure of being demanded in such situations these years. The situation wouldn't turn as bad as before even if you still take no drink after being asked to.

Interviewer: Turning bad? Demand…

1: As 3 had mentioned, you felt that you might get kicked out if you didn't drink. Now it's all different. Most of the time, people would think it's ok to drink half of the glass or take a sip if they don't have to drive. They would then drink in response if I make a toast to them. It's also fine if they don't drink.

Interviewer: Has the rest of you been like this?

3: Actually, it would be less likely to see something like this if you happen to drink with staff from the Chang-Gung Memorial Hospital. You might see differently at other hospitals. This has something got to do with the hospitals the doctors are from. Doctors of Chang-Gung Memorial Hospital are somewhat more reserved. Those from other hospitals are different.

Interviewer: So you have heard….

3: There is no such thing! As for me, I would feel shy. I feel shy to act wild after drinking. You should follow the crowd who are mostly drunk and it is not ok to remain sober alone. Doctors of other hospitals really tend to drink more wildly. Doctors from Chang-Gung Memorial Hospital are well-refined people. This has got something to do with the type of people you are drinking with. It wouldn't be too wild if you are drinking with doctors from Chang-Gung Memorial Hospital. You may meet doctors from other hospitals at the events held by certain medical society or associations.

4: It would be terrible.

Interviewer: Together with the doctors from other hospitals.

3: Chang Gung has relatively more boring doctors. You can put it this way. We would come up with excuses when it comes to drinking alcohol. Those from other hospitals aren't like this but more open-minded and wild.
(ha ha ha ha)

Interviewer: Any unforgettable experiences?

3: You should go to Ching-Yi or doctors from other hospitals. They drink differently from us.

4: During the events held by medical societies, this is more interesting to have the influence of different hospitals. It would be an interesting idea to publish [in the paper], the differences between south and north [parts of Taiwan].

3: There will be differences due to doctors being from either a medical center or clinic; or from the north or south.

Interviewer: Drinking experiences with departments?

1: It's mainly about socializing. You can think for yourself. The point is not to drink seriously.

Interviewer: Do you normally eat out with your colleagues.

3: I sure do! But generally, it depends on the type of colleagues you are going with. Basically, you would drink more if you are eating out with one or two people; you wouldn't have any alcohol to drink when eating out with 4 or 5 people.

Interviewer: 2, did you have any drinking experience from medical society events? How to make a toast to each other.

2: There are always teachers with high ranks at those medical society events. We must drink. If the elders or teachers ask you to drink then we will do so. This is to show some basic respect to your teachers. It's not because you drink. They come to you with excitement and you get to meet them.

1: Out of respect.

Interviewer: Will the elders urge people to drink?

2: They will be somewhat excited. "Come on, everyone, let's drink." It's ok if you don't really drink it down but at least show to your teachers that you have attempted to move the glass closer to your lips.

Interviewer: What are your views on the work-related drinking behavior associated with the drinking experience at gatherings. What are the motives? Does it mean anything to anyone or to you?

3: Why do we have to drink? Why do people drink at social gatherings? As for me, a key point is that disinhibition definitely means euphoria. This is the point, disinhibition, alcohol makes this happen in people. If others follow the crowd to also make a fool of themselves. An unpleasant experience. This is crucial. Everyone would have this kind of mindset, a mindset of complicity structure. This mindset helps stabilize our friendship. When all the kids decide to smoke something, this attempt leads to a closer relationship among them. The social significance behind the drinking culture. The reason to why I would feel embarrassed is because everyone is complicit except for myself. I feel embarrassed if I don't drink. I feel bad if I am the only one who doesn't drink. Everyone is drunk except for me but I drink. People drink for euphoria. Disinhibition means a type of suppression [meant to say free from suppression]. When there is alcohol together with delicious food, you eat something to relax. When everyone is drinking together, you would also drink. This is the behavior of complicity structure. The drinking behavior of men among peers is considered mostly important as the culture of visiting prostitutes in the society. Men go together to visit prostitutes or they can do many things together instead of really going to prostitutes. Some certain drinking culture is like so but now there isn't. In the drinking culture, we become best mates under this drinking culture influence. You may ask what this really means to a man's career or Asian culture. What it means personally or in terms of a group. Different social behavior. It's not hard to realize this…

Interviewer: There is a difference between the Western and Asian Cultures. In the West, it's less likely to see the behavior of doctors making a toast or being urged to drink.

3: It depends on the regions.
4: [Doing it] out of respect, we have to show respect.
3: There is a huge difference in the drinking culture of Westerners. It's quite different that we usually drink with the association to death. What we do is purely for drinking or taking strong alcoholic drinks. Their most important drinking culture is [about] disinhibition, one-night stand, all the stuff that young people do.
1: You had a deep insight into this.
(ha ha ha)
3: The drinking culture of a bar. I had been there [to a bar]. There is this "teammate" spirit in the drinking culture making it similar to our drinking culture. They would not drink at home alone when they are feeling down but would go to a bar instead. If there happens to be another girl feeling down and drinking alone [at the bar], chances are that they will get together. This is rare in Taiwan.
Interviewer: Any other doctors with their views towards drinking at work-related gatherings? What's the meaning behind all this?
2: You can look at this in 2 ways. One is purely for showing up and meeting your colleagues and elders in order to build a closer relationship at those spring receptions or medical society functions. You drink when your boss or elders ask you to drink and everyone is happy. The second one is to drink along while you are eating out with 2 or 3 of your friends. Chatting and drinking make people more relaxed. Everyone has some chats or gets a little tipsy. It's always better to have some alcohol along with meals. People like us having a lot of work stress will feel a little relaxed after eating and taking some alcohol. These are 2 different situations. One is to drink with bottoms up while the other is to drink slowly and chat at the same time.
Interviewer: You just talked about a great deal of your work stress, where is this stress coming from?
2: It's what it is like for being a doctor. Anyone could have work stress.
Interviewer: What's your view about the meaning of this kind of drinking behavior to the rest of you?
4: The same as what others had said, nothing special. You can take it as a tool. You do it out of respect or social etiquette. You might happen to like alcohol and you would give it a try. It's nothing but considered to be a tool. A tool for making yourself relaxed and it is no different from the food or water you eat or drink. It's a tool.
Interviewer: You feel like it's [something to do with] socializing or engaging in business-related social interactions. Everyone, each person has their own views [about this]. 2, When you talked about drinking at work-related gathering, do you think it will have an effect on your work?
4: This is to ask about any improper doctor-patient relationship.
3: Doctors who stay here [in this hospital] are quite self-disciplined and reserved. Those who come back with alcohol and a hangover are said to be alcoholic.
Interviewer: You ever heard about things like this before.

3: I had seen some young people from outside, those who enjoy drinking instead of drinking for social purposes. They love to have drinks along with gourmet food.
Interviewer: Was the doctor's work performance being affected?
3: I wouldn't know as they are from other hospitals.
Interviewer: What does the rest of you think? Does it [drinking] do anything to your daily medical practice?
2: I only take it socially. I would know where my limits are. I would only drink if there is no work the next day, or only drink a little if there is no major operations or nothing requires to be focused on the next day. I haven't had been influenced by alcohol in any way the next day. I don't think I have.

Interviewer: You reckon it has no influence. Would you personally prepare alcohol for any divisional gathering or year-end parties?
3: It's for showing manners. You would prepare gifts of any kind but it doesn't have to be alcohol. However, it's the most convenient for doctors to prepare alcohol. Patients might give you alcohol as a gift. Sometimes I feel embarrassed if this happens. I am not sure about a girl giving a strong alcohol as a gift.
Interviewer: Do other doctors arrange a drinking event if they are to hold some gathering activities?
4: What do you mean?
3: Wine-tasting event?
Interviewer: No, to prepare alcohol of any kind. Wouldn't you prepare alcohol for events like year-end parties or divisional gatherings?
1: If I hold such events.
Interviewer: Yes.
3: Yes, I will. I sure will prepare alcohol if I am the host.
1: For me, it depends on the guests. Say if I am holding a year-end party at my lab where there are mostly youngsters, I probably wouldn't [prepare any alcohol].
Interviewer: What if they are doctors.
1: If those are doctors, I will if I have ever become a chief physician.
Interviewer: Why?
1: Like 3 mentioned earlier, I would take available alcohol from home to see if anyone is up for it. This is a way of manners. I won't force anyone who doesn't want to drink. It's no harm to leave the alcohol untouched.
2: It depends on the people you have meals with. If those people do drink we would prepare some good wine for them to have a taste. Even if they don't necessarily drink and there is some regular alcohol available at the restaurant, we would all still drink a little for the sake of gathering together as friends or colleagues. For any special or memorial purpose such as year-end parties where there will be adults, children, men, women, teachers, new residents, interns and clerks, it is most likely that I will prepare [alcohol] if I am hosting it. Whether to drink or not depends on if there is anyone who opens the bottle.
Interviewer: How about the rest of you?

4: A tool. This is how it is.

Interviewer: Why?

4: A tool when needed. You leave the tool when you don't feel like drinking. When everyone needs it to make a toast to each other and meet others, this ritual is needed, and it's called a tool.

Interviewer: A ritual you said?

4: A ritual, like 3 mentioned, becomes a complicity structure. You definitely get to meet each other through making a toast! Nowadays, you wouldn't develop acquaintances through offering cigarettes to people, but you do this by making a toast instead.

Interviewer: A tool used to make a toast to one another.

4: A tool for knowing each other. An excuse.

Interviewer: It sounds like all you doctors would prepare alcohol for holding activities.

Interviewer: Ok. Lastly, does anyone have anything to add or think of anything you might miss out to mention?

4: You should be able to classify your population by now. About the kind of alcohol, the simplest way is to go to a reference that should contain [information about] strong or weaker alcohol, or things like the classification of quantifying. I reckon I don't know much about the studies they do in human nutrition.

3: How much you would drink, approximately how much. This study design is defined based on social drinking or drinking as a hobby. People like those two there who would drink normally are [thought as] social drinking. This one here who drinks none at all is control. An improper doctor-patient relationship has to be defined. A topic. Doctors wouldn't drink with their patients. Basically, it's rare for doctors to drink with their patients. It's highly unlikely..about this doctor-patient relationship. This kind of person, who would drink alcohol to the extent that their doctor-patient relationship is affected, is unable to stay long in the Chang-Gung Memorial Hospital. For people like me who don't take alcohol, there is zero chance of making an improper doctor-patient relationship. Even if someone who is a heavy drinker knows how to avoid the hangover from previous night causing an improper doctor-patient relationship the next day. Normally a doctor has a hangover the previous night, when they wake up the next morning, they would call in sick by saying something like having the runs so they wouldn't be able to show up. Just imagine yourself looking completely drunk in front of peers, your subordinates would look down on you if you happen to be their chief physician. If you are just a lower rank doctor your supervisor will be breathing down your neck. Right? It's impossible to find a case like this in reality. You should make Ching-Yi Lee to ask doctors from public hospitals under ministry of health. To obtain cases of improper doctor-patient relationship in doctors from Chang Gung is highly unlikely to happen in Chang Gung hospitals. I have never seen it or heard anything like it in Chang Gung hospitals. Unless you are able to go to a public hospital anonymously, it's impossible to collect such cases. This is really how it is! Improper doctor-

patient relationship, a mistake in the operation or saying something that offends others.

Interviewer: The problem you talked about just now is not so… there is no direct relative relations.

4: It’s a wrong subject. Medical professionals should be divided. The quantity of drinking amount should be classified. And improper doctor-patient relationship should be defined.

3: These are what we should talk about in our own time and there is no need to be this precise.

Group 12

Residents (n=5)

Group 12

Interviewer: Let's get to know each other. Please introduce yourself with your position title. Any particular type of food you like to eat?
5: I am a resident now and I have no idea about the type of food I like to eat. I like to try and eat anything yummy. I also like to try anything amazing. It should be ok. I am unable to name the food I dislike. I like to eat green pepper, carrots. I don't know about things that are tasteless. That's all.
1: Resident. Seafood is what I like! I like ice wine if we are talking about alcohol. I only like ice wine. I like alcohol with sweet taste and beer wouldn't be my favorite.
2: The type of food I like is more like veges. Only meat of high quality is acceptable to me. It melts as it goes into the mouth. Beef tongue! Grilled beef tongue of high quality or something like that. It's not limited to any particular type. Something like this. I am a R2 wandering around in the division of surgery.
3: Resident. I eat everything, no food I like in particular.
4: I am a neurosurgery R2. The type of food [I like] is Japanese cuisine.

Interviewer: Everyone is into all different kinds of food. Do you usually drink alcohol during a meal? Do you usually drink?
1: As when we normally go to or get off work every day?
Interviewer: Not every day but do you usually drink?
2: Although I have announced that I am trying to quit I still drink.
5: How incredible a R2 is! This interview is all yours. The five of us altogether may still not drink as much as you do.
2: I don't drink much.
Interviewer: Where do you drink?
2: I would buy beer or alcohol of sweet taste when I drink at home. Work is tiring. I would buy some fried chicken to go with alcohol on the day I get too frustrated from work. I drink to relax myself or when I go to restaurants or karaoke with my friends.
Interviewer: At home, at gatherings in a restaurant or karaoke. How often would this be? How many times a month?
2: The number of times you drink or get drunk?
(laughs)
Interviewer: How about we talk about the number of times you drink first.
2: Well, it's about once to twice a month if it's for gatherings. We eat and drink at gatherings or drink at karaoke.
Interviewer: What about at home?
2: It depends. [Once] every week or two weeks. It totally depends on my mood and there is no certain frequency.
Interviewer: About once every week or every two weeks. How about the others?
3: I would drink some beer with braised dishes.
Interviewer: Where? At home?
3: If I take food home I drink beer along with anything of strong taste.
Interviewer: How often do you drink?
3: It depends on when I buy it.

5: I am not so into drinking. I only like drinking beer.
(ha ha)
Interviewer: How many times do you drink a week?
3: It depends.
Interviewer: It depends, how about the number of times you drink per month?
3: About 10 times a month.
Interviewer: Do you eat out with your friends?
3: We don’t usually drink at gatherings because we either ride [motorbikes] or drive.
Interviewer: What about the rest of you?
5: I don’t drink when I am alone. Yeah. I wouldn’t drink unless someone else is with me. Basically, I prefer wine with a sweet taste. I can’t stand purely beer or red wine alone.
Interviewer: At a restaurant?
2: Yes. I will also drink along when my family members feel like drinking at meals, but I definitely won’t drink alone.
Interviewer: About how often you would drink?
5: It depends on how many gatherings are there in that month. There won’t be more than 5 days a month.
Interviewer: 3 to 4 times a month.
5: Something like that. I will only drink when invited to gatherings by others.
Interviewer: 4, what about you?
4: Only drink mostly on weekends. About once or twice a month, it depends. I will drink craft beer when going to a bar or beer bar.
Interviewer: About once to twice a month at a bar, is it with your friends or colleagues?
4: It depends.
Interviewer: Friends or colleagues.
1: Only at gathering occasions, I seldom drink alone. However, I do buy ice wine home to drink with my mom because she loves to drink. I don’t usually drink by myself. I also drink at birthday celebrations or with friends at karaoke if they have prepared drinks.
Interviewer: How often is this?
1: Not very often. Once a month approximately.

Interviewer: You all have attended divisional gatherings or year-end parties, haven’t you? Would you care to share the experience of going to spring receptions or welcome parties?
2: There wasn’t too much drinking occasion. We had red wine at welcome parties.
Interviewer: I heard that at welcome parties, everyone would be asked to drink….
2: No.
Interviewer: It depends on which division you are from?
2: Yes. In surgery where there was mostly the elderly who left early wouldn’t take too much to drink. The remaining youngsters stayed behind to get hyper. It’s not too bad as no one was drunk when they left [the parties].

4: It’s those chief physicians or teachers who would urge others to drink or ask others to make a toast. It depends on the culture that exists in that particular division. I have heard about what happened in some other divisions where they would drink to the point of ending up in emergency room.

Interviewer: Which division?

4: ENT, URO.

2: Drink until you have IBS.

4: A wheelchair was ready any time when needed on that day. Every year is like so. About other divisions, there might be hardly any alcohol at other divisions. It’s like at our division, we hardly drink any alcohol or at the year-end parties.

Interviewer: No drinking at all at the year-end or welcome parties?

4: There might be some alcohol at the year-end or welcome parties but we won’t keep on making a toast and drinking until we drop.

2: We would briefly make a toast out of courtesy and wrap it up shortly afterwards.

4: It depends on the type of tradition they own in their division.

Interviewer: Do these other divisions refer to other colleagues?

4: I have heard about some ridiculous drinking events going on at some divisions. It depends on the hospitals. It might be different for hospitals of northern, central or southern regions. Beer is common for the hospitals from the south. Several six-packs of beer are around, the newcomers will be asked to finish the six-pack first before the event officially begins.

2: Rumor has it that in the division of obstetrics and gynecology, there is a senior visiting staff making a rule of drinking 2 glasses of alcohol in response to a R2 resident, with the idea of R2 meaning 2 glasses, but for himself being R25, the R2 resident would have to drink 25 glasses in return.

Interviewer: In which department exactly did this happen?

2: When I made the rounds at the wards of obstetrics and gynecology, it was many years ago. It’s rare nowadays.

Interviewer: At Chang Gung [hospital] and many years ago. Have other doctors heard anything like this? 3, have you?

3: No. Like what 4 had said about what happened in the ENT where most doctors were forced to drink and ended up in emergency room. This happened at Linkou branch as well as Mackay Hospital.

Interviewer: Could you talk about your personal experience? What was it like in a situation like that?

2: I read about it on the fb post of a colleague from ENT.

Interviewer: Had you personally attended this kind of gathering? Any divisional gathering?

3: I haven’t attended any year-end parties. I don’t have any divisional gathering. PGYs don’t have any divisional gatherings.

Interviewer: What about the rest of you?

1: We drink a little but never seen anything like completely smashed from drinking. When I was an intern during the internal medicine rounds and there happened to be a gathering where the whole division of internal medicine was involved, we managed to take alcohol moderately.

Interviewer: Drinking moderately did you say?

1: It was like you should go and grab whatever alcohol is available over there and pour yourself a drink! Your supervisors would complain about you drinking too little and it would be a waste if you don't drink till you drop. I was an intern during the gathering held by the division of internal medicine.

Interviewer: Did you make a toast to each other?

1: The chief physician did come to our table. It was Ji-Tseng Fang who came to chat with us at our table and talk about how his intern year was like. We briefly proposed a toast to each other.

Interviewer: Did the chief physician kept on making a toast to people?

1: No. Doctors of internal medicine tend to be shy and they won't pressure others.

Interviewer: Have you heard about experiences like that? Like what 4 just said, any experience of going to a drinking event?

5: Okay! Actually, I do.

1: What were you thinking to say that you don't have such experiences?! Just joking.

2: You should say it earlier!

(ha ha ha)

5: It's more fun to say it when everyone is finished with their story. As a medical student, each one of us has a mentor. Basically, a mentor would hold a mentor meeting once every month or every 3 months. Our mentor happens to enjoy drinking. He is Ming-Yang Chang, a doctor of obstetrics and gynecology. He would take a bottle of wine to the gathering every time. Once he even deliberately turned a mentor gathering into a wine tasting event!

1: Did he specifically sign up all of you, everyone was signed up for the event?

5: Yes. We were all in the wine-tasting event where you could see a lot of glasses there. One glass for a certain type of wine. There were heaps. We started wine-tasting with the cheapest first. Finally, you would end up drinking a bottle of wine of a very high quality, something costs like more than $5,000. It does taste good. At that time, I was drinking until I had a severe headache. It was a terrible headache and I could barely walk. Drinking red wine until throwing up at the mentor gathering.

Interviewer: The mentor took you to drink?

5: Yes, to have a wine-tasting.

Interviewer: Did you have to make a toast?

5: Yes. We had to do it but we wouldn't attempt any games as to outdrink others. No one would do this with red wine. You had to hold a goblet of wine and made a toast to your mentor. You only had to take a few sips. It was rare to try outdrinking others, except when you were in the military. This wouldn't happen in the hospitals. You are not allowed to drink alcohol when serving in the military but there is always people who managed to smuggle in alcohol. Anything from sorghum liquor to beer. I wasn't involved that much in drinking. Basically, if they asked you to go "bottoms up" you would definitely have to drink up and finish that glass on your hand. Those who came from Kinmen were really extreme. They would take our 100cc beer cup with the brand label of Taiwan Beer and fill it with the sorghum liquor. They would try to outdrink others. I can't imagine myself doing that and I was totally

shocked. This only happens in the military. It is just more classy to drink red wine at hospitals.

Interviewer: This was the experience from serving in the military. Have you got other work-related drinking experience? Haven't you been in this kind of social events before?

1: No. We would only drink out of courtesy instead of outdrinking at gatherings. Yes, we had been to that kind of events.

Interviewer: You had been there.

1: Something like the year-end parties at the division of internal medicine.

5: We would drink a little.

Interviewer: Any unforgettable experience or interactions with the teachers?

1: We were all pretty well mannered, didn't see any doctors…

5: Gentle.

1: We were not like some business men making some bets.

5: I haven't seen anything like it. Only in the military is where you would see the most ridiculous thing going on.

Interviewer: 4 had mentioned about a similar situation seen in another division?

4: It depends on the custom of the division or the hospital.

Interviewer: Have you personally been to one of those?

4: I had been to one welcome party held in Mackay Memorial Hospital. People were so drunk and had to be carried by others. It is often to see that people drink till they drop mostly at welcome parties. It's not meant for picking on the newcomers but being a newcomer is more like you would hardly dare to speak up. A newcomer is more likely to be completely smashed by being forced into drinking. The most extreme drinking occasion would definitely not be found at year-end parties where participants are mostly gentler and required to make some performance. It is more likely to see newcomers being pressured to drink and getting completely smashed at those welcome parties where you can find the most extreme drinking occasion.

Interviewer: This was something happened at the welcome party of another division.

4: Yeah, the year-end parties are ok and drinking isn't so much of a focus.

Interviewer: Have you known any from other divisions, the experience of drinking at gatherings?

2: I have seen something like E1, V1 and F1 posted on facebook.

Interviewer: Your previous experience?

2: It's just something I have heard.

5: It wasn't long since we have entered the workforce.

Interviewer: It was your teacher whom you were drinking with?

4: Teachers are people you have the most interactions with at this workplace. This is what it is like at Chang Gung, we won't have too much going on with sales people from companies but with colleagues or teachers instead.

Interviewer: I heard about the interactions with sales people…in previous interviews this was mentioned.

1: It's not so much these days but only in the past time.

2: It's normal for the pharmaceutical sales rep to treat the doctors to dinner in order to promote their drug or introduce a paper. They would also hold some

education-related banquet where doctors are invited to make a presentation. It’s more academic rather than just a drinking event.

Interviewer: You all have experience of drinking with your colleagues or teachers. Would it change the way you usually see them? Such as your views on seeing them being so decent at work but acting differently at a drinking event.
2: We would drink with the people whom we have known quite well and in fact they are also best friends in private. We wouldn’t drink with those unfamiliar to us. For me, it doesn’t seem to make too much difference in the way they behave.
5: Well, I certainly have met someone who has shown a difference in the way he behaves. He is also an emergency physician currently.
1: How did you get to see him acting differently?
3: You do have a lot of experience [in this].
2: We should try and make him say it.
3: Who are the doctors at the emergency department?
2: Who. Let’s say the names together.
5: It’s pretty obvious! Everyone likes to push him to drink and you will see it after everyone is done forcing him into drinking. It has been like this since college and we are in the same year. It’s too obvious. He is normally pretty decent at work and hardly expressing his opinions about anything. Once he has taken alcohol he would go rant about anything, literally anything around him and make comments on everything. A completely different person he has become.
Interviewer: Would this affect the way you see him?
5: No. It was fun to watch because he is fine when he gets sober. He just turns to a different person under the influence of alcohol.
Interviewer: A different person he has become, he might seem pretty ridiculous?
5: Yes. A person who is normally decent at work and everyone finds it interesting to invite someone like him to gatherings, so they can push him to drink to see him turning into a different person.
1: Did you tell him afterwards?
5: Yeah, we did. He doesn’t seem to care so it’s ok.
Interviewer: Would this affect your views about him?
5: It’s only about someone being like this under the influence of alcohol but this doesn’t affect his work performance.
Interviewer: You would specifically ask him to drink then.
5: No. It’s just fun to watch him turning into another person. It’s ok to drink at events where appropriate. He is just more likely to be pushed to drink a lot and everyone likes to see him “perform”. This has totally got nothing to do with his performance at work because normally he won’t drink right before or after work.
Interviewer: Doctors will make him drink?
2: Would you set him up if you are out with him?

5: We wouldn't drink together is there is only 2 of us. He is only getting pushed to drink when there is a group of people and that is more fun. It is boring to make him drink when you are the only one doing so.
Interviewer: Has any of you heard about this too?
1: Mostly something like myself falling asleep when drunk.
Interviewer: Being forced into drinking by other colleagues, anything like this you have met?
1: No, not so much at the workplace.
5: Most of our colleagues are also our classmates.
2: We would drink with those whom we have known well and met from college.

Interviewer: Do you think drinking at a gathering would have an effect on the way you perform at work?
2: Don't drink till you drop and it wouldn't cause any problem.
5: Never have I been once being too drunk with a hangover at work. Never drink to end up with a hangover.
2: Having some extreme headache.
5: The extreme headache you get is just the same as that you get from having no sleep at all on the day when you are on duty.
Interviewer: 3, what is your view on this?
3: I would usually drink on the night before, like 2 had said, it gets fine the next day. It's no big deal to have a headache and work performance won't be affected.
Interviewer: Work is not affected by the headache?
3: The mind is still clear despite the headache.
Interviewer: Do you think it will affect your medical practice?
2: You still just show up for work. It won't affect decision-making or anything when it comes to handling matter. This is like going to work when sick and there won't be any major influence.
Interviewer: You would only feel unwell but this won't affect anything.
2: Yup, that is right.
Interviewer: The rest of you?
5: A terrible headache but your mind is pretty clear. It totally has no effect or whatsoever on your judgement. Besides, you would only drink at home or when you get off work.
2: It's usually on the day before the next day when you are free.
5: Yeah. If I have to make rounds about 5am early next morning and stay on call in the evening, I wouldn't be stupid to take alcohol or drink till I drop the night before this. No, I wouldn't.
Interviewer: What does the rest of you think? This won't have an effect on the interaction with your patients.
4: I would choose to drink on weekends.
2: I try to avoid drinking on workdays and friends whom we drink with are also colleagues. We wouldn't pick the day when everyone has to work the next day.
Interviewer: How often do you attend such drinking event?

1: As I had said, it was about once a month. Drink when invited. We would arrange a time on weekends or when most of our colleagues are off-duty. People would automatically refuse to attend anyway if they have to be on duty.

Interviewer: About what 4 had talked about on urging each other to drink, what do you think the motive is for this work-related drinking behavior?

4: Motives? A welcome party is like a ritual. To push him to drink in excess is like a way of showing him who the boss is! It’s easy to pick on the lower rank and young ones. Nobody dares to push the director to drink. Only the directors or chief physicians can do so to their subordinates. It’s impossible for a person of a lower rank to urge their supervisors to drink. It’s like a ritual, a welcoming ritual.

Interviewer: You think the drinking behavior is like a welcoming ritual?

4 Yes, theoretically it won’t have an effect on your later work performance. You often started without any work experience. You may tend to talk more after being pushed to drink and this may help you open up a better relationship with others.

Interviewer: As for the others, what is the motive behind this work-related drinking behavior?

1: The definition of work-related drinking events is like..

Interviewer: Something like the drinking behavior, the meaning behind it as we just talked about.

2: I think it’s the idea of showing respect to each other during regular gatherings where you make a toast in general. If it’s to push others to drink it would be a fast way to get to know people at something like a welcome party where you don’t know anyone. You are pushed to drink until you are drunk and caught off-guard. Your seniors get to see you in the worst predicament and this might make you feel easy to get over anything that is worse to come. If it’s about year-end parties, I have no idea, I haven’t met any.

Interviewer: 5, what do you think?

5: I hadn’t been in any of those events. I don’t know.

3: There is one coming up for you this year.

5: There was an evening event of spring reception when I was an intern. However, I couldn’t drink at all as I was the host for the evening. There wasn’t anything of pushing people to drink or things like that at that particular event. People had a chat and no one was trying to outdrink others. There was no such thing as that I must drink more than you or push any particular person to drink, nothing like this to be seen there.

Interviewer: This is what you saw when you were hosting that event?

1: Yup, something I saw in a general evening event and also in a big event.

Interviewer: 3?

3: Same as what 5 had said, it is to drink to socialize or create a bustling atmosphere. Nothing much to say. For spring receptions, most people were students at that time during spring receptions for interns. Everyone attended for the food provided there. You won’t be judged for how much you drink. As a student at that time, you probably wouldn’t do anything other than having the meals there.

Interviewer: Did the teachers go to make a toast to you when you were an intern?
5: They would come to us, but you only had to drink one glass in return. A representative director from some division or teaching division of some sort made a toast to everyone and we would drink this very first glass of alcohol in response to them. Just this first one and the rest has got nothing to do with us.
Interviewer: Making a toast in a round?
5: No, no.
1: They would say to the whole table, “Come, let’s drink, you guys had done well, great job!” and have some drink before going to the next table.
5: What I meant to say is that when the director came as a representative of their whole group, we would only have to drink this very first glass in return then it’s finished.
Interviewer: Were there a lot of teachers and mentors?
5: A table of them.
3: One table of them is the most. There were 20-30 tables of students.
Interviewer: People reckon it was a good chance to create a bustling atmosphere and make friends.
1: It’s also to show your manners. It wouldn’t look nice to take out the juice. Drinking wine and proposing a toast is not only out of politeness but also to show your manners.
5: Social etiquette.
Interviewer: You had never experienced being forced to drink then?

Interviewer: Would you arrange a drinking event if you have to hold a dinner gathering or activities like this?
5: It’s hard to avoid. If there is 3 of us having a dinner gathering and there is also alcohol provided at that restaurant, we might have some to drink but not too much. It also depends on the type of gathering.
Interviewer: If it’s a gathering held by the division or a year-end party.
2: Drinking alcohol is never our main purpose at a dinner gathering. People who like to drink will make their order. The amount you would take depends on how the dinner gathering goes. You are less likely to get drunk, say if people you are eating with there ain’t so hyper, otherwise it would be weird if you are the only one getting so eager to drink.
Interviewer: It depends then.
1: It’s not a must for alcohol to be provided at dinner gatherings or you go to a gathering only when alcohol is provided.
Interviewer: What if you personally hold such an event?
2: You meant a dinner gathering with friends or the year-end parties?
Interviewer: I meant something like the year-end parties held in the division.
2: If it’s not some big event a drinking occasion is less likely to happen.
1: The divisional gathering is not as frequent as you might think. It’s only once a year.
Interviewer: Have you ever been to any medical society events?
1: That’s also like once a year. I hadn’t been to many of them.

Interviewer: Mostly dinner gatherings with colleagues.

4: For the medical society events, it also depends. Every time there is a lot of fresh R1s in the neurosurgery society, they would be extensively pushed to drink at those events, and only the youngest ones would experience this.

Interviewer: You meant neurosurgery?

4: Yes, this is exactly what would happen at our neurosurgery society events. It also depends on the custom of a hospital. They drink beer, Taiwan Beer and whiskey is a must at Cheng Kung University Hospital. Red wine is for Taiwan University Hospital while juice is for Chang Gung Hospital.

(Everyone laughs)

4: It depends on the hospitals. You can see extensive drinking events going on at Taipei Medical University Hospital, Wanfang Hospital and Shuang Ho Hospital.

2: I think it's more extensive at Mackay Hospital.

1: We drink in moderation at Chang Gung. Did you interview doctors from other hospitals?

Interviewer: No, we didn't.

4: Comparing to others, we don't have much extensive drinking at Chang Gung. It is pretty obvious at our society events. There is this dinner gathering once a year through the years from R1 to R6 and there is at least a 6 of them in total every year. There is one dinner banquet held by our society once every two months. There is a huge difference that you can see easily. Doctors from those 3 hospitals mentioned earlier would drink and mix every drink they have got. Beer and whiskey for the Cheng Kung University Hospital. Those from Taiwan University Hospital and Chang Gung Hospital would hardly drink any and they would usually hide to avoid drinking.

1: Those from Taiwan University Hospital are good mates.

Interviewer: Have you known others from other hospitals?

4: Yes, they would have some extreme drinking going on at other hospitals.

Interviewer: If they are the colleagues you know, would you think differently about them?

4: No. It would have got something to do with the custom of that division, workload and your daily routine. Everyone from your division who gets off work at 6 or 7pm is always tired as hell and eager to rest at home after work. They would like to relax a bit on weekends. At other hospitals, there is usually not many important events most of the time except for a major operation occasionally. Everyone would be excited and happy after the operation is done, and they can go to drink till they drop if there is nothing going on the next day. I think there is a difference, plus, that we don't accept invitations to free meals by pharmaceutical companies at our hospital, this would also make a difference. There are always sponsors for the monthly gathering event which they have every month at the hospitals of northern region. Unlike other hospitals, it's usually those chief physicians who would personally arrange funding for divisional gathering at our hospital. The CR at other hospitals would tell their sponsor to pay all the expenses. This is a huge difference!

Interviewer: You have seen some extensive drinking at other hospitals or division?

4: Whether or not this would have an effect on.. or whether it’s good or bad… It might be either good or bad for people doing this. Work performance being affected or not… it looks pretty much the same to me. It’s a medical center after all.
Interviewer: Would the professional image be [affected]…?
4: No. Everyone is a good friend. There are only about 20 new residents admitted to neurosurgery department every year. There isn’t many of them. I am only a R2 with my number being 500 something and it’s best to help each other out. Drinking together doesn’t harm the relationship. Unlike some people who would get into a drunken brawl, there is no such thing for us as getting into a fight after drinking. It’s likely that drinking may enhance the relationship as you tend to tell the truth after having some alcohol to drink.
Interviewer: You hadn’t personally run into any of these?
4: I have heard about this has an influence on a person choosing a specialty in the hospital. What I heard is that doctors from the last generation of surgery division are into drinking very much, and this particular doctor who loves to perform surgeries chooses his specialty in gastroenterology or otolaryngology instead of surgery to avoid the drinking custom that exists in the surgery division.
5: These 2 divisions seem to be more notorious [for its drinking custom] than surgery.

Interviewer: You wouldn’t necessarily provide alcohol at the events you hold personally. Would you keep the same custom at these activities if one day you have become a supervisor?
5: It’s ok to have some alcohol to drink and we won’t push others to drink. If they don’t want to drink leave them be. What’s the point of forcing others?
4: We are doctors after all.
5: Yup, it doesn’t mean you are better if you are able to urge others to drink. There is also a result of headache from drinking that you have to consider.
Interviewer: What about you, 3?
3: It depends on the custom of the division.
Interviewer: Which means?
3: We don’t have a division.
5: They were assuming the time when you will become a supervisor.
3: Well, I just said it.
(ha ha ha)
Interviewer: Ok, anyone got anything to add?
1: This custom is not popular at our hospital.
Interviewer: It’s less likely to have such experiences.
1: We have student-looking clerks who are extremely young attending our mentor gathering. It’s lucky that things like making a fool of yourself when drunk or consistently pushing others to drink never happen. There is never something such as forcing others to drink.
Interviewer: Anything else?

4: I am already in the specific specialty. There should be a sense of belonging among colleagues. Doctors from surgery would want to form a bond with the PGY interns by having a drink with them sometimes. I would usually feel bad to refuse [drink offers] before coming into this specialty. Is it to get a sense of belonging after drinking or to drink because of a sense of belonging?

Interviewer: We will end our interview here.
